# Chetomin, a SARS-CoV-2 3C-like Protease (3CL^pro^) Inhibitor: *In Silico* Screening, Enzyme Docking, Molecular Dynamics and Pharmacokinetics Analysis

**DOI:** 10.3390/v15010250

**Published:** 2023-01-15

**Authors:** Mahmoud A. A. Ibrahim, Alaa H. M. Abdelrahman, Dina E. M. Mohamed, Khlood A. A. Abdeljawaad, Mohamed Ahmed Naeem, Gamal A. Gabr, Ahmed M. Shawky, Mahmoud E. S. Soliman, Peter A. Sidhom, Paul W. Paré, Mohamed-Elamir F. Hegazy

**Affiliations:** 1 Computational Chemistry Laboratory, Chemistry Department, Faculty of Science, Minia University, Minia 61519, Egypt; 2 School of Health Sciences, University of KwaZulu-Natal, Westville, Durban 4000, South Africa; 3 Ain Shams University Specialized Hospital, Ain Shams University, Cairo 11588, Egypt; 4 Department of Pharmacology and Toxicology, College of Pharmacy, Prince Sattam Bin Abdulaziz University, Al-Kharj 11942, Saudi Arabia; 5 Agricultural Genetic Engineering Research Institute (AGERI), Agricultural Research Center, Giza 12619, Egypt; 6 Science and Technology Unit (STU), Umm Al-Qura University, Makkah 21955, Saudi Arabia; 7 Molecular Modelling and Drug Design Research Group, School of Health Sciences, University of KwaZulu-Natal, Westville, Durban 4000, South Africa; 8 Department of Pharmaceutical Chemistry, Faculty of Pharmacy, Tanta University, Tanta 31527, Egypt; 9 Department of Chemistry & Biochemistry, Texas Tech University, Lubbock, TX 79409, USA; 10 Chemistry of Medicinal Plants Department, National Research Centre, 33 El-Bohouth St., Dokki, Giza 12622, Egypt

**Keywords:** marine natural products (MNPs), SARS-CoV-2 3CL^pro^, docking computations, MD simulation, ADMET study

## Abstract

The emergence of the Coronavirus Disease 2019 (COVID-19) pandemic caused by severe acute respiratory syndrome coronavirus-2 (SARS-CoV-2) has led to over 6 million deaths. The 3C-like protease (3CL^pro^) enzyme of the SARS-CoV-2 virus is an attractive druggable target for exploring therapeutic drug candidates to combat COVID-19 due to its key function in viral replication. Marine natural products (MNPs) have attracted considerable attention as alternative sources of antiviral drug candidates. In looking for potential 3CL^pro^ inhibitors, the MNP database (>14,000 molecules) was virtually screened against 3CL^pro^ with the assistance of molecular docking computations. The performance of AutoDock and OEDocking software in anticipating the ligand-3CL^pro^ binding mode was first validated according to the available experimental data. Based on the docking scores, the most potent MNPs were further subjected to molecular dynamics (MD) simulations, and the binding affinities of those molecules were computed using the MM-GBSA approach. According to MM-GBSA//200 ns MD simulations, chetomin (UMHMNP1403367) exhibited a higher binding affinity against 3CL^pro^ than XF7, with Δ*G*_binding_ values of −55.5 and −43.7 kcal/mol, respectively. The steadiness and tightness of chetomin with 3CL^pro^ were evaluated, revealing the high stabilization of chetomin (UMHMNP1403367) inside the binding pocket of 3CL^pro^ throughout 200 ns MD simulations. The physicochemical and pharmacokinetic features of chetomin were also predicted, and the oral bioavailability of chetomin was demonstrated. Furthermore, the potentiality of chetomin analogues –namely, chetomin A-D– as 3CL^pro^ inhibitors was investigated. These results warrant further *in vivo* and *in vitro* assays of chetomin (UMHMNP1403367) as a promising anti-COVID-19 drug candidate.

## 1. Introduction

COVID-19 is caused by severe acute respiratory syndrome coronavirus-2 (SARS-CoV-2), which has produced a global health emergency [1,2]. In December 2019, SARS-CoV-2 was first identified in Wuhan, China, and quickly spread around the world [3,4,5]. As of October 2022, over 623 million COVID-19 cases were confirmed, resulting in over 6.5 million deaths. COVID-19 has exposed the fragility of public health and healthcare systems in many nations [6]. Despite the predictions of viral abatement due to widespread vaccine production using next-generation technologies, rapidly evolving variants combined with vaccine hesitancy and limited vaccine availability have thwarted plans for viral control [7]. Indeed, now that the SARS-CoV-2 pandemic has been raging for two-and-a-half years, most people have either been vaccinated against and/or infected by the virus. The threats of the appearance of more genetic variations with greater virulence and the elevated rate of viral impedance to authorized therapeutics are driving an uninterrupted necessity for exploring efficient anti-COVID-19 therapies.

SARS-CoV-2 3C-like protease (3CL^pro^/M^pro^/NSP5) is a vital enzyme in viral gene replication, transcription, and expression [8,9,10,11]. Since the outbreak of COVID-19, several experimental and computational trials have been conducted to repurpose various drugs to inhibit 3CL^pro^ and combat this disease [12,13]. Among the repurposed drugs, remdesivir, lopinavir, umifenovir, ritonavir, and favipiravir, have been promoted for clinical investigation as COVID-19 medications [14,15]. Very recently, the FDA (US Food and Drug Administration) granted a contingency utilizing permission for a combination of nirmatrelvir and ritonavir as therapeutics for COVID-19 treatment.

Plant-based natural products (NPs) have been considered a substantial source of curative agents. Indeed, NPs have robust antiviral activity and have demonstrated inhibitory efficiency against numerous viral enzymes [16,17]; exploratory antiviral drugs include cyclosporine and cyanovirin-N [18,19].

An alternate source of natural products is marine secondary metabolites [20,21]. The marine environment is a rich source of chemically diverse and biologically active natural molecules. It has been reported that MNPs demonstrate antimalarial, antifungal, antibacterial, and antiviral activities [22,23]. The antiviral activity of MNPs has been investigated for dengue, influenza, SARS, and other viruses [24,25]. For instance, polyphenol and coumarin derivatives from MNP sources demonstrated high *in vitro* activities toward 3CL^pro^ [26,27]. However, the potentiality of MNPs as 3CL^pro^ inhibitors has yet to be examined extensively. Here, we explore MNPs as 3CL^pro^ inhibitors by screening a database containing >14,000 compounds using *in Silico* molecular docking techniques. The most effective MNPs were submitted for molecular dynamics simulations. MNPs-3CL^pro^ binding affinities were calculated utilizing the MM-GBSA approach. These *in Silico* calculations in combination with predicted physicochemical and pharmacokinetics characteristics, identified multiple drug candidates for COVID-19 treatment.

## 2. Materials and Methods

### 2.1. 3CL^pro^ Preparation

The 3D structure of 3CL^pro^ complexed with a noncovalent inhibitor ((5*S*)-5-(3-{3-chloro-5-[(2-chlorophenyl)methoxy]phenyl}-2-oxo [2H-[1,3’-bipyridine]]-5-yl)pyrimidine-2,4(3H,5H)-dione; XF7) was retrieved from the PDB database (PDB ID: 7L13 [28], resolution: 2.17 Å) and employed as a template for the current study. All heteroatoms, ligands, ions, and crystallographic water molecules were removed. The H++ server was utilized to identify residues’ protonation states, and missing hydrogen atoms were added [29].

### 2.2. Database Preparation

**Figure 1:**
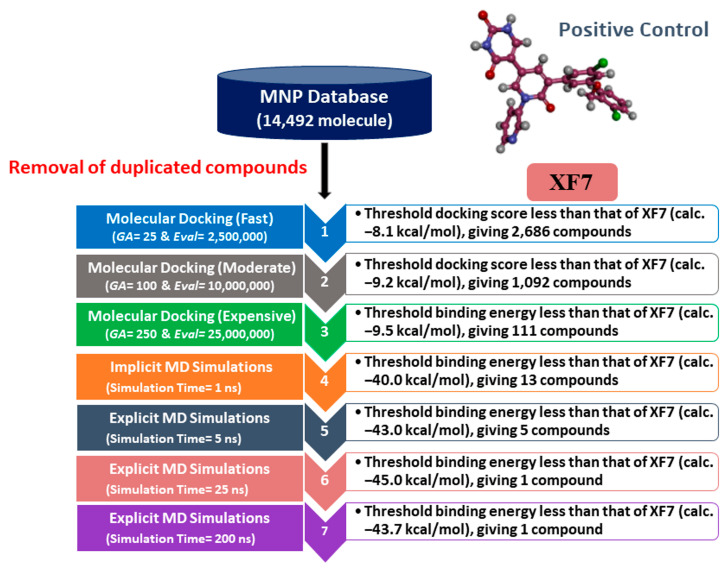
A schematic diagram of the utilized *in Silico* computations in the filtration process of marine natural products (MNP) database.

The marine natural product (MNP) database was downloaded in an SDF format from Prof. Encinar’s website (http://docking.umh.es/downloaddb (accessed on 11 February 2022)). The 3D chemical structures were generated using Omega2 software with a maximum of 200 conformers generated within a 10 kcal/mol energy window [30,31]; the lowest energy conformer was selected for further studies. The protonation state of each MNP was investigated using fixpka within QUACPAC software [32]. The Gasteiger–Marsili method was used to appoint the MNP atomic charges [33]. Based on the InChIKey (IUPAC international chemical identifier keys), duplicated molecules were eliminated [34]. The prepared MNP library is accessible via www.compchem.net/ccdb. The schematic diagram of the utilized *in Silico* computations for the filtration process of the MNP library is represented in Figure 1.

### 2.3. Molecular Docking

The initial molecular docking computations were performed using AutoDock4.2.6 [35] and OEDocking3.0.1 [36,37] software. For the AutoDock4.2.6 calculations, the pdbqt file of 3CL^pro^ was created in accordance with the AutoDock protocol with the assistance of the molecular graphics laboratory (MGL) tools 1.5.7 [38]. The internal conformational search was executed by the LGA (Lamarckian Genetic Algorithm) [39]. The LGA population size and the maximum number of generations were 300 and 27,000, respectively. Three accuracy levels of docking computations were utilized, namely, fast, moderate, and expensive (i.e., more computational cost and time) calculations. The *GA* (number of genetic algorithms) run variables were 25, 100, and 250 for fast, moderate, and expensive molecular docking computations, respectively. In addition, the *eval* (maximum number of energy evaluations) was 2,500,000, 10,000,000, and 25,000,000 for fast, moderate, and expensive molecular docking computations, respectively. The grid dimensions (60 × 60 × 60 Å^3^) were able to embrace the complete binding pocket of 3CL^pro^. The coordinates of the grid center were x = −13.069, y = 9.740, z = 64.83. The employed grid is depicted in Figure S1. The predicted poses were clusterized using the internal conformational cluster engine with an RMSD tolerance value of 1.0 Å. The representative docking mode was chosen from the largest cluster with the lowest docking score. For OEDocking3.0.1 software, the resolution of the exhaustive search was set to high, and the best scoring pose was retained. Based on the complex of the co-crystallized XF7 ligand with 3CL^pro^, the active site was determined. XF7 was found to be one of the most potent noncovalent inhibitors toward 3CL^pro^ with an IC_50_ value of 0.018 μM [28].

### 2.4. MD Simulations

MD (molecular dynamics) simulations were performed for the most potent MNPs complexed with 3CL^pro^ with the assistance of AMBER16 software [40]. The details of the utilized protocol for the MD simulations are described elsewhere [41,42,43,44,45]. Briefly, the investigated MNPs were parameterized by a general AMBER force field (GAFF2) [46]. 3CL^pro^ was characterized using the AMBER force field of 14SB [47]. In the current study, implicit and explicit water solvent MD simulations were performed.

In the context of implicit water solvent MD simulation, the investigated MNPs were optimized at MMFF94S, and the atomic charges of the MNP were assigned using the AM1-BCC approach [48]. For the nonbonded interactions, the cutoff value was adjusted to 999 Å. Additionally, non-periodic boundary conditions were utilized. Moreover, the solvent model (implicit generalized born (igb) = 1) was employed to estimate the solvation impact [49]. The docked MNPs complexed with 3CL^pro^ were initially minimized based on 500 cycles. The minimized complexes were slowly heated from 0 to 310 K throughout 10 ps in six steps. After that, the investigated systems were equilibrated over a 50 ps simulation. Ultimately, the equilibrated systems underwent a production stage of 1 ns.

In the context of explicit water solvent MD, the investigated MNPs were optimized at HF/6-31G* level of theory by Gaussian09 software [50]. The RESP (restrained electrostatic potential) approach was subsequently applied to compute the charges of the MNPs [51]. All of the MNP-3CL^pro^ complexes were solvated utilizing TIP3P water molecules in an octahedral box with a minimal distance of 12 Å. Using the tleap tool implemented inside AMBER16 software, the counter-ions (52 Na^+^ and/or 48 Cl^−^) were inserted to attain 0.15 M NaCl concentration. Afterward, minimization was conducted using combined steepest descent and conjugate gradient algorithms for 5000 cycles to eliminate any unsuitable geometries or steric clashes. The minimized systems were progressively heated up to 310 K over 50 ps. Thereafter, the investigated systems were equilibrated for 10 ns. Eventually, the equilibrated systems were subjected to production runs over 5, 25, and 200 ns MD. For electrostatics, the Particle-Mesh Ewald (PME) algorithm was used [52]. An atomic cutoff distance of 12 Å was utilized for short-range nonbonded interactions [52]. All of the bonds that included hydrogen atoms were constrained using a SHAKE algorithm [53]. All of the MD simulations were conducted utilizing a GPU-accelerated MD engine in AMBER16 (pmemd.cuda). The CompChem GPU/CPU hybrid cluster was used to perform all *in Silico* computations. All graphical representations were visualized utilizing BIOVIA Discovery Studio Visualizer 2020 [54].

### 2.5. Binding Affinity Computations

The molecular mechanical-generalized Born surface area (MM-GBSA) approach within the AMBER16 software was used to compute the binding affinities for the most potent MNPs complexed with 3CL^pro^ [55]. For MM-GBSA calculations, the frames were collected every 10 ps and the Δ*G*_binding_ was estimated according to the following equation:

$$\Delta G_{\mathrm{binding}}=G_{\mathrm{Complex}}-\left(G_{\mathrm{MNP}}+G_{3CL^{pro}}\right)$$

where the *G* term is:

$$G=G_{\mathrm{GB}}+E_{\mathrm{ele}}+G_{\mathrm{SA}}+E_{\mathrm{vdw}}$$

*G*_GB_ stands for electrostatic solvation-free energy. *E*_ele_ is electrostatic energy. *G*_SA_ refers to the nonpolar solvation-free energy that is evaluated from the SASA (solvent-accessible surface area) with the assistance of an LCPO algorithm [56]. *E*_vdw_ is van der Waals energy. Because of its greater computational costs, the computation of entropy (*S*) was overlooked [57,58].

### 2.6. Physicochemical Features

The physicochemical characteristics of the most potent MNP were estimated utilizing the SwissADME server [59]. Under the framework of the Lipinski’s rule, five characteristics were considered, including the molecular weight (MWt ≤ 500 g/mol), hydrogen bond donors (HBD ≤ 5), log *p* value (log *p*o/w ≤ 5), demonstrating good oral and intestinal absorption, topological polar surface area (TPSA ≤ 140 Å^2^), revealing that the compounds have eminent oral absorption or membrane permeability, and hydrogen bond acceptors (HBA ≤ 10). Passing these characteristics indicates that a given MNP is orally bioavailable.

### 2.7. Pharmacokinetic Characteristics

An online pkCSM tool was employed to anticipate the ADMET properties of the most potent MNPs [60]. Absorption (A) involves skin permeability, Caco2 permeability, HIA (human intestinal absorption), skin permeability, and P-glycoprotein substrate and inhibitor. Distribution (D) includes fraction unbound, CNS (central nervous system) permeability, VDss (steady-state volume of distribution), and BBB (blood–brain barrier) permeability. The metabolism (M) depends on factors such as CYP2D6/CYP3A4 substrate and cytochrome P450 inhibitors. The excretion (E) is estimated via inhibitor total clearance. The toxicity (T) is anticipated via AMES toxicity and skin sensitization.

## 3. Results and Discussion

The COVID-19 epidemic and its emerging variants underline the necessity to develop alternative treatments, as vaccines alone do not provide complete protection against illness. The 3C-like protease (3CL^pro^) has a vital function in viral replication and, as such, is deemed an effective druggable target. Moreover, marine natural products (MNPs) include several metabolites with antiviral properties and, as such, are potential drug candidates for COVID-19 treatment [61]. Here, we utilized *in silico* approaches to screen a chemical library containing > 14,000 MNP metabolites as potential SARS-CoV-2 3CL^pro^ inhibitors.

### 3.1. Docking Assessment

**Figure 2:**
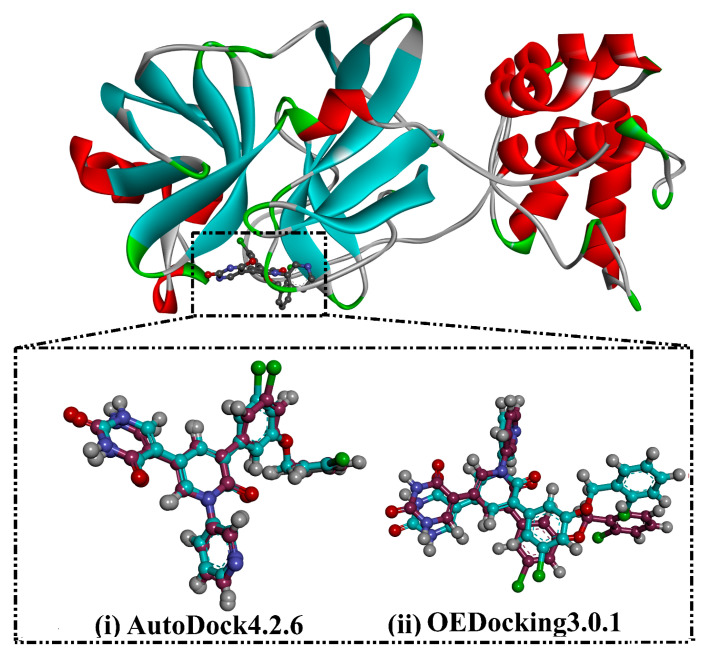
3D molecular interaction of the experimental structure (in dark red) and the portended docking pose (in cyan) of XF7 in complex with the 3CL^pro^ using (**i**) AutoDock and (**ii**) OEDocking software, respectively.

Two molecular docking engines, AutoDock and OEDocking, were used to validate the inhibitor-3CL^pro^ binding mode. Initially, the binding mode of the XF7, a co-crystallized ligand in the 3CL^pro^ binding pocket, was predicted and compared with the resolved native structure (PDB ID: 7L13 [28]) (Figure 2). The predicted docking pose was almost identical to the native binding mode with RMSD values of 0.20 and 0.43 Å for AutoDock and OEDocking, respectively (Figure 2). Comparing the RMSD values of AutoDock and OEDocking, it can be seen that AutoDock predicted the native inhibitor-3CL^pro^ binding mode better than OEDocking. As a result, AutoDock was selected in the filtration process of the MNP database.

### 3.2. MNP Database Screening

Initially, the MNP database was screened against 3CL^pro^ using fast docking computation. On the basis of the anticipated docking scores, 2686 MNPs displayed docking scores lower than that of the XF7 ligand, with a value of −8.1 kcal/mol against 3CL^pro^. Consequently, those 2686 MNPs were subjected to moderate docking computations. The computed docking scores for the top 2686 MNPs are summarized in Table S1. According to the estimated moderate docking scores, 1092 MNPs showed docking scores less than that of the XF7 ligand, with a value of −9.2 kcal/mol. Thus, the 1092 MNPs were submitted to expensive docking computations, and the evaluated docking scores are listed in Table S2. Interestingly, about 10% of the selected MNPs (i.e., 111 MNPs) displayed lower docking scores than XF7 (calc. −9.5 kcal/mol). The predicted docking scores, binding features, and 2D chemical structures for the thirteen most potent MNPs for 3CL^pro^ are shown in Table 1. The 2D docking poses for these selected MNPs are depicted in Figure S2. These MNPs were selected based on binding affinities over 1 ns implicit water solvent MD simulations, as described in Section 3.3. Generally, the 2D representations demonstrated the hydrogen bonding of those MNPs with GLN189, GLY143, and GLU166 residues in the 3CL^pro^ binding pocket (Figure S2). π-based, hydrophobic, and vdW interactions were also monitored between the identified MNPs and key residues in the 3CL^pro^ binding pocket (Figure S2).

**Figure 3:**
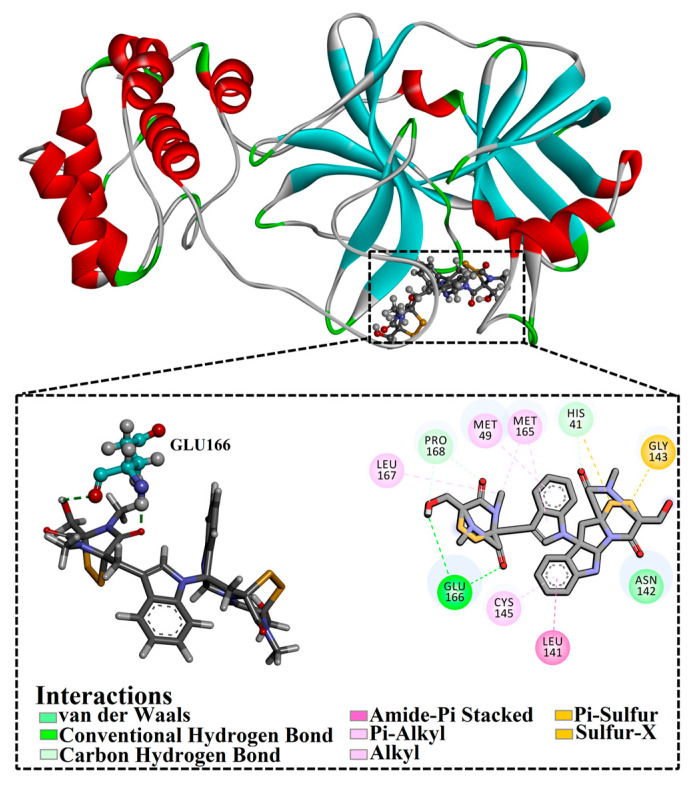
Three- and two-dimensional molecular interaction patterns of the binding mode of chetomin (UMHMNP1403367) with SARS-CoV-2 3CL^pro^.

Chetomin (UMHMNP1403367), an organic heteropentacyclic compound isolated from *chaetomium globosum* and *farrowia seminuda*, demonstrated the lowest docking score against 3CL^pro^ with a value of −13.4 kcal/mol. Investigating the docking pose of UMHMNP1403367 within the 3CL^pro^’s active site disclosed that the CO and OH of the (1*S*,4*S*)-1-(hydroxymethyl)-2,3-dithia-5,7-diazabicyclo[2.2.2]octane-6,8-dione ring form two hydrogen bonds with the NH and CO of GLU166 with distances of 1.99 and 2.79 Å, respectively (Figure 3).

Compared to chetomin (UMHMNP1403367), XF7 complexed with 3CL^pro^ demonstrated a competitive docking score with a value of −9.5 kcal/mol (Table 1). The robust binding of XF7 with 3CL^pro^ is ascribed to the ability to form hydrogen bonds with THR26 (2.28, 2.49 Å), GLY143 (2.07 Å), SER144 (3.29 Å), HIS163 (1.91 Å), and GLU166 (1.81 Å) (Table 1).

### 3.3. MD Simulations

**Figure 4:**
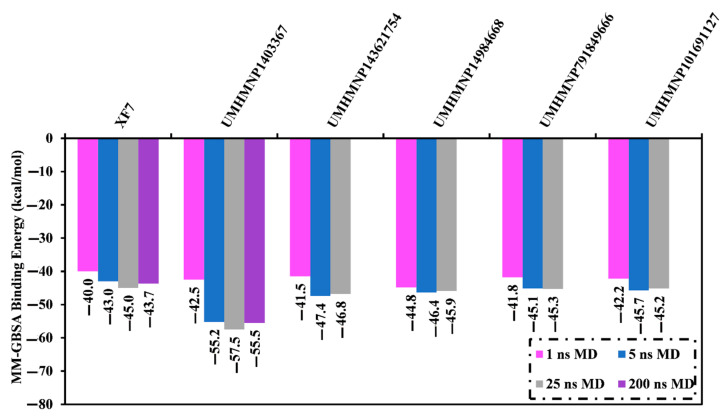
Estimated binding affinities for the five most potent MNPs and the co-crystallized XF7 ligand in complex with 3CL^pro^ over 1 ns in an implicit water solvent and 5, 25, and 200 ns in an explicit water solvent MD.

MD simulations are utilized to establish the stabilization of the ligand–target complex, structural specifics, conformational elasticities, and trustworthiness of ligand–target binding energy [62,63]. The most promising MNPs (111 molecules with docking scores <−9.5 kcal/mol) complexed with 3CL^pro^ were submitted to MD simulations and pursued by binding energy computations. To diminish the time and computational costs, the simulations were executed for 1 ns MD in an implicit water solvent. The corresponding binding energies were evaluated, and thirteen MNPs manifested lower Δ*G*_binding_ compared to the native XF7 ligand (calc. −40.0 kcal/mol) (Table S3). These MNPs in complex with 3CL^pro^ were then submitted to 5 ns MD in an explicit water solvent to gain more reliable binding energies (Table S4). Based on the estimated MM-GBSA binding energies over 5 ns MD simulations, only five MNPs unveiled lower Δ*G*_binding_ compared to the native XF7 ligand (calc. −43.0 kcal/mol) (Figure 4 and Table S4). These potent MNPs were then subjected to a 25 ns MD simulation, and the corresponding MM-GBSA binding energies were estimated (Figure 4). Interestingly, out of the five identified MNPs as potential 3CL^pro^ inhibitors, only chetomin (UMHMNP1403367) displayed stationary binding energies with Δ*G*_binding_ values of −55.2 and −57.5 kcal/mol throughout 5 ns and 25 ns MD simulations. While UMHMNP143621754, UMHMNP14984668, UMHMNP791849666, and UMHMNP101691127 manifested a tenuous rise in binding energy (Δ*G*_binding_) over the 25 ns MD simulation. This elucidates the importance of long MD simulation to foretell MNP-3CL^pro^ binding energy. As well, the relative binding energy (ΔΔ*G*_binding_) values for the five identified MNPs compounds with respect to the XF7 were computed (Table S5). From Table S5, UMHMNP1403367 demonstrated a promising ΔΔ*G*_binding_ value of −12.5 kcal/mol, while the other four MNPs showed insignificant ΔΔ*G*_binding_ values of ≤−1.8 kcal/mol. Therefore, MD simulation for chetomin (UMHMNP1403367) complexed with 3CL^pro^ was protracted to 200 ns. In addition, the corresponding binding affinity was computed (Figure 4).

**Figure 5:**
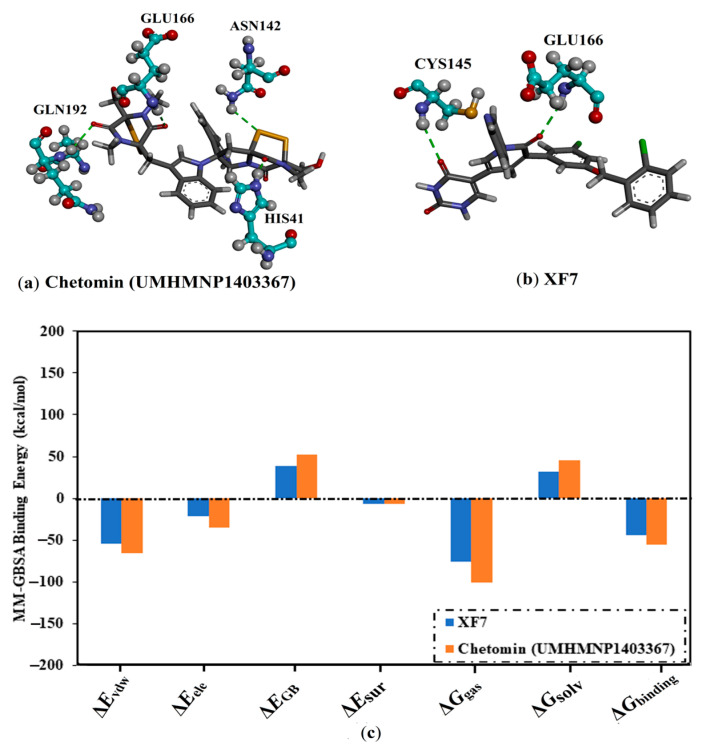
3D molecular interaction pattern of binding modes of (**a**) chetomin and (**b**) XF7 complexed with 3CL^pro^ based on the average structure, and (**c**) components of the MM-GBSA binding energies throughout the MD course of 200 ns.

The perceptible disproportion between the estimated binding energies for the chetomin-3CL^pro^ complex over the 25 ns and 200 ns MD simulations was not observed. Compared with the native XF7 ligand (calc. −43.7 kcal/mol), chetomin was revealed to have a lower binding energy against 3CL^pro^ throughout a 200 ns MD, with an average Δ*G*_binding_ of −55.5 kcal/mol (Figure 4). The 3D and 2D molecular interactions of average the structures of chetomin and XF7 inside the 3CL^pro^ binding pocket over 200 ns are illustrated in Figure 5 and Figure S3, respectively. Inspecting the binding mode of chetomin inside the binding pocket of the 3CL^pro^ showed that chetomin preserved its hydrogen bond with GLU166 and formed hydrogen bonds with HIS41, ASN142, and GLN192 residues (Figure 5 and Figure S3). Notably, those hydrogen bonds were absent in the docked pose of chetomin (Figure 3), demonstrating the importance of conducting MD simulation. More exactly, the two CO groups and OH of (1*S*,4*S*)-1-(hydroxymethyl)-2,3-dithia-5,7-diazabicyclo[2.2.2]octane-6,8-dione ring exhibit three hydrogen bonds with the CO and NH of GLU166 and NH of GLN192 with distances of 1.92, 2.67, and 2.59 Å, respectively (Figure 5). Moreover, the sulfur atom and CO of (1*R*,4*R*)-2,3-dithia-5,7-diazabicyclo[2.2.2]octane-6,8-dione ring demonstrated two hydrogen bonds with NH_2_ of ASN142 and NH of the imidazole ring of HIS41 with distances of 2.81 and 1.91 Å, respectively (Figure 5 and Figure S3).

For XF7 complexed with 3CL^pro^, the CO of pyridin-2(1H)-one and nitrogen of the pyridine ring interact with NH of GLU166 and SH of CYS145 by hydrogen bonds with lengths of 1.85 and 1.90 Å, respectively (Figure 5 and Figure S3).

The computed binding affinities were decomposed into individual components to explore the main driving forces in the binding of chetomin and XF7 with SARS-CoV-2 3CL^pro^ (Figure 5). As shown in Figure 5, *E*_ele_ was a favorable contributor in the binding affinities of chetomin and XF7 with 3CL^pro^, with values of −34.5 and −21.7 kcal/mol, respectively (Figure 5). The binding affinity of chetomin and XF7 with 3CL^pro^ were dominated by *E*_vdw_ interactions with an average value of −55.5 and −54.5 kcal/mol, respectively (Figure 5). Notably, *E*_vdw_ is about one and a half fold stronger than *E*_ele_.

**Figure 6:**
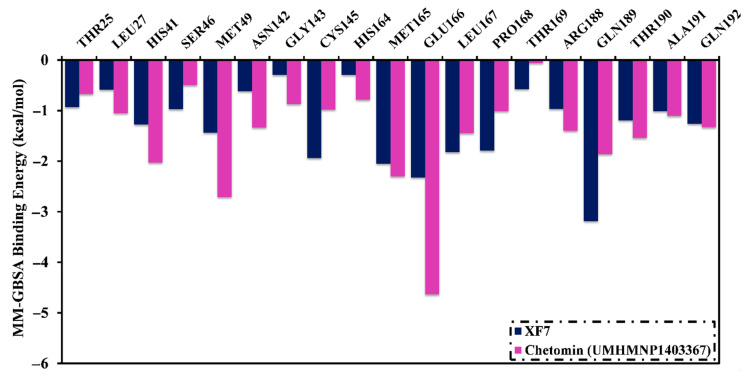
Per-residue decomposition of the binding affinity (kcal/mol) of chetomin (UMHMNP1403367) and XF7 complexed with 3CL^pro^ over 200 ns MD simulations.

To inspect the participation of essential residues in the ligand–target complexes, the total Δ*G*_binding_ values were decomposed at the per-residue level. Only amino acids with Δ*G*_binding_ < −0.50 kcal/mol were demonstrated (Figure 6). It is apparent that GLU166, ASN142, HIS163, and GLN189 residues participated in the interactions of chetomin and XF7 with 3CL^pro^. Significant participation of the GLU166 residue to the total Δ*G*_binding_ was noticed with values of −4.1 and −2.3 kcal/mol for chetomin (UMHMNP1403367)- and XF7-3CL^pro^ complexes, respectively (Figure 6).

### 3.4. Post-MD Analyses

To further investigate the structural and energetical stability of chetomin and XF7 in the complex with 3CL^pro^, post-MD analyses were performed throughout the 200 ns MD simulations.

#### 3.4.1. Binding Affinity Analysis

**Figure 7:**
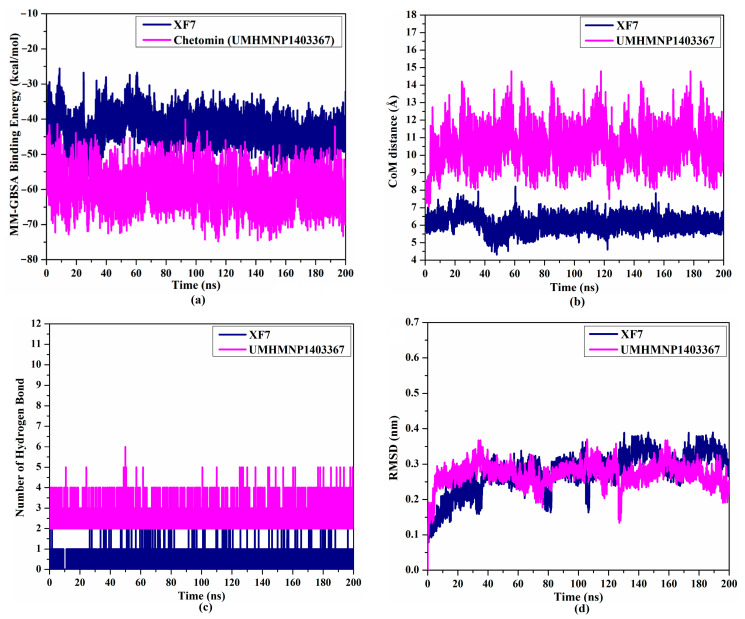
(**a**) Estimated binding energy per frame, (**b**) center-of-mass (CoM) distance, (**c**) the number of H-bonds, and (**d**) root-mean-square deviation (RMSD) of the backbone atoms from the starting structure of chetomin (pink) and XF7 (navy) towards the 3CL^pro^ throughout 200 ns MD.

The structural stability of chetomin and XF7 in a complex with 3CL^pro^ was inclusively estimated throughout the MD course of 200 ns by gauging the correlation between binding energy and time (Figure 7a). What stands out in Figure 7a is the general constancies of the binding affinities of chetomin and XF7 with 3CL^pro^, with Δ*G*_binding_ values of −55.5 ± 4.5 and −43.7 ± 3.9 kcal/mol, respectively. On the basis of the energetical analysis, all of the inspected complexes maintained stability throughout the 200 ns MD.

#### 3.4.2. CoM Distance

To gain more in-depth insight into the stability of chetomin and XF7 complexed with 3CL^pro^ over the 200 ns MD simulations, the center-of-mass (CoM) distance was evaluated between the investigated ligand and GLU166 (Figure 7b). The most exciting aspect of the CoM graph is the high steadiness of chetomin and XF7 in complex with 3CL^pro^ with average CoM distances of 10.5 and 6.1 Å, respectively (Figure 7b). These findings revealed that the chetomin binds more tightly with the 3CL^pro^ than XF7.

#### 3.4.3. H-Bond Numbers

Furthermore, the steadiness of chetomin and XF7 in a complex with 3CL^pro^ was evaluated by estimating the number of hydrogen bond interactions (H-bonds). The correlation between the number of H-bonds and simulation time was graphed in Figure 7c. As depicted in Figure 7c, the average number of H-bonds was three and one for chetomin- and XF7-3CL^pro^ complexes. Notably, XF7 demonstrated the fewest number of hydrogen bonds with the fundamental residues inside the active site of 3CL^pro^. However, the good binding affinity of XF7 with an average Δ*G*_binding_ of −43.7 kcal/mol may be ascribed to other interactions, such as vdW, π-based, and hydrophobic interactions. The superiority of the vdW interactions of XF7 with 3CL^pro^ conforms to the binding affinity decomposition results (Figure 5). The hydrogen bond analysis confirmed the presentence of considerable stability for the chetomin than XF7 complexed with 3CL^pro^.

#### 3.4.4. Root-Mean-Square Deviation

Throughout the 200 ns MD course, the RMSD (root-mean-square deviation) of the C, Cα, N, and O of the entire system was evaluated to observe the conformational change of the chetomin- and XF7-3CL^pro^ complexes (Figure 7d). As shown in Figure 7d, the investigated systems stabilized after the first 5 ns and maintained their stabilization until the end of the 200 ns simulation. The measured RMSD values with average values were 0.27 and 0.28 nm during the 200 ns MD for the chetomin- and XF7-3CL^pro^ complexes. These findings assured that chetomin is tightly bonded and does not influence the structural steadiness of 3CL^pro^, in addition to preserving the structural integrity.

### 3.5. Physicochemical Features

The SwissADME server was employed to anticipate the potentiality of utilizing chetomin and the co-crystallized XF7 ligand as drugs by estimating the physicochemical characteristics. The physicochemical features involved the topological polar surface area (Å^2^), the MWt (g/mol), HBD, HBA, and N_rotb_ (number of rotatable bonds) (Table 2). As listed in Table 2, the MWt was 710.9 and 533.4 g/mol for chetomin and XF7, respectively (Table 2). Notably, the tenuous rise in MWt will not have tremendous leverage on drug diffusion and transmission, wherever it has been demonstrated that sundry FDA-approved drugs proceeded beyond the conventional low MWt of 500 g/mol [64]. The Log *p* values of chetomin and XF7 were auspicious, with values lower than 5 [65]. In addition, the number of HBD was less than 5, and the number of HBA was less than 10. These findings indicated that chetomin is a potential anti-COVID-19 drug candidate.

### 3.6. Pharmacokinetic Characteristics

The knowledge of pharmacokinetics and toxicity features offers worthy guidelines for starting-stage drug discovery. Caco2 permeability and HIA are absorption characteristics that must be considered in any medicine exploration process [66]. The investigated compounds demonstrated perfect absorption, with HIA values of 73.4% and 93.5% for chetomin and XF7, respectively (Table 3). The inspected compounds unveiled satisfactory skin permeability, with a value of −2.7 for both compounds (Table 3). The investigated compounds also demonstrated good Caco2 permeability (less than 0.9 cm/s). One of the most fundamental ADMET characteristics is the P-glycoprotein substrate/inhibitor. Chetomin and XF7 were characterized as inhibitors/substrates for P-glycoprotein (Table 3). The BBB membrane permeability, VDss, and CNS were estimated to check drug distribution. Significant distribution volumes were noticed for chetomin and XF7 with log BB values of −2.0 and −1.1, respectively, indicating that these compounds can readily pass the blood–brain barrier (BBB permeability) (Table 3). For VDss and CNS permeability, log VDss and log PS values were −0.1 and 0.3 and −3.9 and −2.5 for chetomin and XF7, respectively (Table 3). CYP450 has a fundamental function in the metabolism of the drug. The metabolism predictions exposed that chetomin and XF7 cannot inhibit CYP2D6 enzymes and cannot act as inhibitors for CYP1A2, CYP2C19, CYP2C9, and CYP3A4 enzymes (Table 3).

The total drug clearance was 0.3 and 0.8 mL/min/kg for chetomin and XF7, respectively (Table 3). Toxicity has an extraordinary role in opting for adequate drugs. Chetomin and XF7 did not expose AMES toxicity and skin sensitization (Table 3). According to the predicted ADMET features, these findings deduced that the investigated compounds might be used as potential anti-COVID-19 drug candidates.

### 3.7. Chetomin Derivatives as Prospective Anti-COVID-19 Drug Candidates

Based on the promising potentiality of chetomin as SARS-CoV-2 3CL^pro^, the study was extended to examine the perspectivity of the chetomin analogues, namely, chetomin A-D. The chemical structures of chetomin A-D were retrieved from the PubChem database and prepared for docking computations. The docking scores, binding features, and 2D chemical structures of the chetomin derivatives are listed in Table 4. As enrolled in Table 4, all of the chetomin derivatives demonstrated similar docking poses with 3CL^pro^, exhibiting hydrogen bonds with GLU166, ASN142, and GYS145. The anticipated docking scores for chetomin derivatives ranged from −11.1 to −12.2 kcal/mol. A comparison of the docking results revealed that chetomin (UMHMNP1403367/PubChem10417379) demonstrated the lowest docking score with a value of −13.4 kcal/mol (Table 4).

## 4. Conclusions

Currently, COVID-19 is spreading quickly worldwide, causing a high mortality rate and morbidity rate. The demand for COVID-19 treatments still very much exists. By *in silico* screening and characterization of MNPs as potential 3CL^pro^ inhibitors, several candidate metabolites were identified. Included in that initial screening, chetomin was shown to have promising binding energy (Δ*G*_binding_), with a value of −55.5 kcal/mol against 3CL^pro^ over 200 ns MD simulation. Post-MD analyses throughout 200 ns MD simulations indicated the high stability of chetomin complexed with 3CL^pro^. Chetomin also showed favorable physicochemical and pharmacokinetic features. Comparing the docking scores of chetomin with its analogues, namely, chetomin A-D, demonstrated the superior binding affinity of chetomin against 3CL^pro^. These findings clearly clarify the suitability of chetomin as a promising drug candidate for further studies toward new COVID-19 treatments.

## Figures and Tables

**Table 1:** Evaluated fast, moderate, and expensive docking scores, binding features, and 2D chemical structures for most potent MNPs against 3CL^pro a^.

No.	Compound ID	Docking Score (kcal/mol)			2D Chemical Structure	Binding Feature ^b^
		Fast	Moderate	Expensive		
	XF7	−8.1	−9.2	−9.5	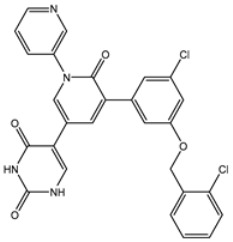	THR26 (2.28, 2.49 Å), GLY143 (2.07 Å), SER144 (3.29 Å), HIS163 (1.91 Å), GLU166 (1.81 Å)
1	UMHMNP1403367 (Chemotin)	−11.7	−12.2	−13.4	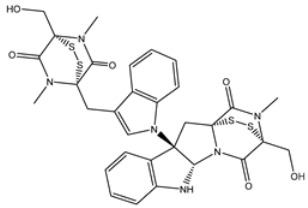	GLU166 (1.99, 2.79 Å)
2	UMHMNP101691127	−11.4	−12.1	−12.3	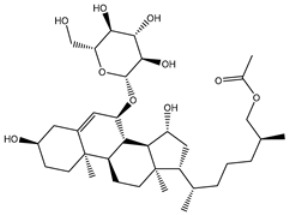	LEU141 (2.01, 2.22 Å), CYS145 (2.33 Å), GLU166 (2.01, 2.84 Å), GLN189 (1.89 Å)
3	UMHMNP791849666	−11.4	−11.9	−12.3	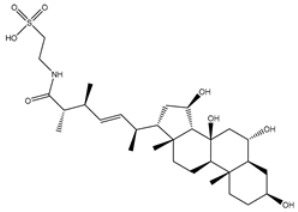	ASN142 (1.96 Å), HIS163 (1.93 Å), GLU166 (2.01, 2.09 Å), ARG188 (1.78 Å)
4	UMHMNP14984668	−11.3	−11.7	−12.2	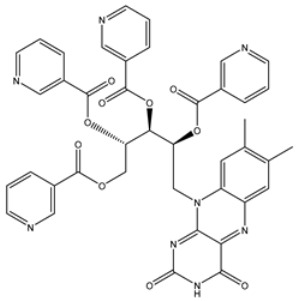	HIS41 (2.41 Å), GLY143 (2.31 Å), SER144 (2.28 Å), CYS145 (2.09, 2.17 Å), ARG188 (2.00 Å)
5	UMHMNP143621754	−11.3	−11.7	−12.2	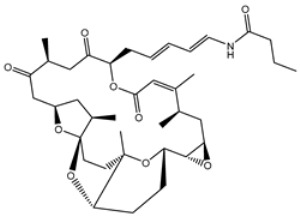	HIS41 (2.14 Å), GLY143 (2.75, 2.91 Å), CYS145 (2.45 Å), GLU166 (2.10, 2.78 Å)
6	UMHMNP148839036	−11.3	−11.6	−11.8	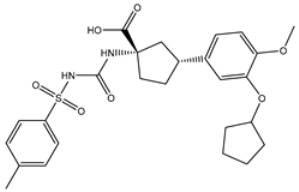	HIS41 (2.20 Å), LEU141 (2.09 Å), CYS145 (2.14, 2.27 Å)
7	UMHMNP133056072	−11.3	−11.6	−11.7	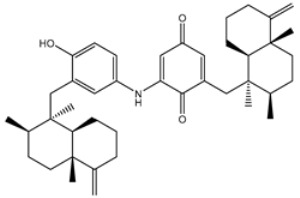	GLU166 (2.30 Å)
8	UMHMNP386274857	−11.3	−11.6	−11.7	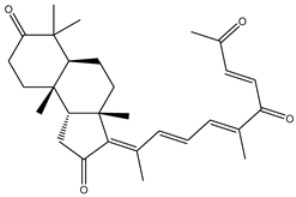	SER144 (1.91 Å), CYS145 (2.41 Å), GLN192 (2.13 Å)
9	UMHMNP133056094	−11.2	−11.5	−11.5	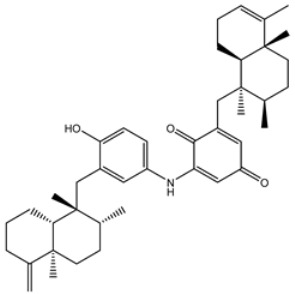	GLU166 (2.82 Å), THR190 (2.56 Å), GLN192 (2.48 Å)
10	UMHMNP26195584	−11.1	−11.5	−11.5	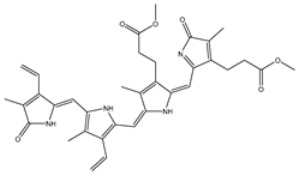	HIS28 (2.28 Å), GLY143 (2.51 Å), CYS145 (2.15, 2.72 Å), HIS163 (1.92 Å), GLN189 (2.29 Å)
11	UMHMNP874383707	−11.1	−11.5	−11.4	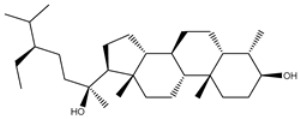	LEU141 (2.08 Å), GLY143 (2.75 Å), SER144 (2.09 Å), CYS145 (2.46 Å), GLU166 (1.75 Å)
12	UMHMNP221163300	−11.1	−11.4	−11.4	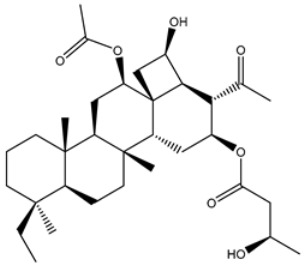	PHE140 (2.04 Å), GLY143 (2.32 Å), CYS145 (2.87 Å), HIS163 (1.92 Å)
13	UMHMNP109152387	−11.1	−11.4	−11.4	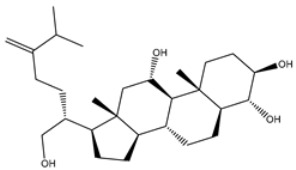	HIS41 (2.25 Å), PHE140 (1.93 Å), GLY143 (2.44 Å), CYS145 (2.35, 2.36 Å), HIS163 (2.00 Å), GLU166 (2.57 Å), HIS172 (2.23 Å)

^a^ Data ranked on the basis of the expensive docking scores. ^b^ Conventional hydrogen bond only is displayed.

**Table 2:** Anticipated physiochemical characteristics of chetomin and XF7 as 3CL^pro^ inhibitors.

Compound ID/Name	Log *p*o/w	MWt	HBD	HBA	TPSA	N_rotb_
XF7	4.25	533.4	2	5	109.8	6
Chetomin	1.05	710.9	3	6	239.9	5

**Table 3:** The ADMET anticipation for chetomin (UMHMNP1403367) and XF7 as anti-COVID-19 drug candidates.

ADMET Characteristics	Chetomin (UMHMNP1403367)	XF7
Absorption (A)		
Skin permeability (log Kp)	−2.7	−2.7
Caco2 permeability (log Papp, cm/s)	−0.05	0.5
Intestinal absorption (human) (%)	73.4%	93.5%
P-glycoprotein substrate (Yes/No)	Yes	Yes
P-glycoprotein I inhibitor (Yes/No)	Yes	Yes
P-glycoprotein II inhibitor (Yes/No)	Yes	Yes
Distribution (D)		
VDss (human) (log L/kg)	−0.1	0.3
BBB permeability (log BB)	−2.0	−1.1
CNS permeability (log PS)	−3.9	−2.5
Metabolism (M)		
CYP1A2 inhibitor (Yes/No)	No	No
CYP2C19 inhibitor (Yes/No)	No	Yes
CYP2C9 inhibitor (Yes/No)	No	Yes
CYP2D6 inhibitor (Yes/No)	No	No
CYP3A4 inhibitor (Yes/No)	No	Yes
Excretion (E)		
Total Clearance (log mL/min/kg)	0.3	0.8
Toxicity (T)		
AMES toxicity (Yes/No)	No	No
Skin Sensitization (Yes/No)	No	No

**Table 4:** Predicted docking scores, binding features, and 2D chemical structures of chetomin analogs as SARS-CoV-2 3CL^pro^ inhibitors.

No.	Compound Name/PubChem ID	Docking Score (kcal/mol)	2D Chemical Structures	Binding Features ^a^
1	PubChem10417379 (Chetomin)	−13.4	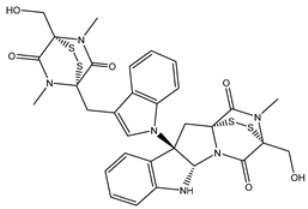	GLU166 (1.99, 2.79 Å)
1	PubChem139591137 (Chetomin B)	−12.2	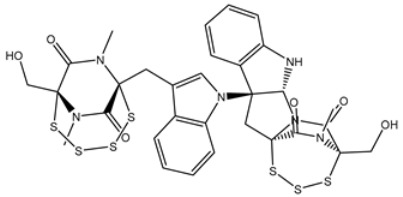	GLU166 (2.69 Å), CYS145 (2.12 Å), HIS41 (1.71 Å)
2	PubChem139591139 (Chetomin D)	−12.0	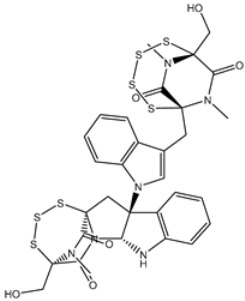	PRO168 (2.07 Å), GLU166 (1.83 Å), CYS145 (2.56, 2.95 Å), LEU141 (1.97 Å)
3	PubChem139591136 (Chetomin A)	−11.5	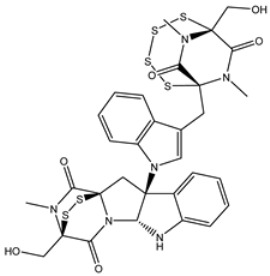	ASN142 (1.88, 2.93, 3.04 Å)
4	PubChem139591138 (Chetomin C)	−11.1	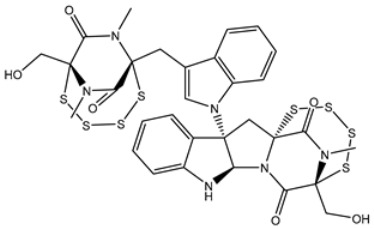	GLN192 (2.46 Å), THR190 (1.88 Å), GLU166 (3.10 Å), ASN142 (2.17 Å)

^a^ Conventional hydrogen bond only is displayed.

## Data Availability

The data presented in this study are available in the Supplementary Materials.

## Supplementary Materials

The following supporting information can be downloaded at: https://www.mdpi.com/article/10.3390/v15010250/s1, Figure S1. The employed grid box in Autodock computations; Figure S2. 2D representation of binding modes and interactions for top thirteen scoring drugs with the key amino acid residues of the 3CL^pro^ binding pocket; Figure S3. 2D molecular interaction pattern of binding modes of (a) UMHMNP1403367 and (b) XF7 complexed with 3CL^pro^ based on the average structure over a 200 ns MD simulation; Table S1. Estimated fast and moderate docking scores (in kcal/mol) for top 2686 MNPs towards 3CL^pro^; Table S2. Estimated fast, moderate, and expensive docking scores for XF7 and the top 1092 potent MNPs compounds within the 3CL^pro^ binding pocket; Table S3. Estimated fast, moderate, and expensive docking scores and MM-GBSA binding energies (in kcal/mol) over 1 ns implicit water solvent MD simulations for XF7 and the top 111 potent MNPs compounds within the 3CL^pro^ binding pocket; Table S4. Estimated fast, moderate, and expensive docking scores and MM-GBSA binding energies (in kcal/mol) over 1 ns implicit-solvent and 5 ns explicit-solvent MD simulations for XF7 and the top 13 potent MNPs compounds within the 3CL^pro^ binding pocket; Table S5. The estimated MM-GBSA binding energies (in kcal/mol) and relative binding energies (ΔΔ*G_binding_*) over 25 ns explicit water solvent MD simulations for XF7 and the top 5 potent MNPs compounds within the 3CL^pro^ binding pocket.
