# Supplementary material for: Chetomin, a SARS-CoV-2 3C-like Protease (3CLpro) Inhibitor: In Silico Screening, Enzyme Docking, Molecular Dynamics and Pharmacokinetics Analysis
Source: Viruses. 2023 Jan 15;15(1):250. doi: 10.3390/v15010250 (PMC9866112; doi:10.3390/v15010250)
Supplement: Supplementary file 1 [file viruses-15-00250-s001.zip › viruses-2070885-supplementary.pdf]

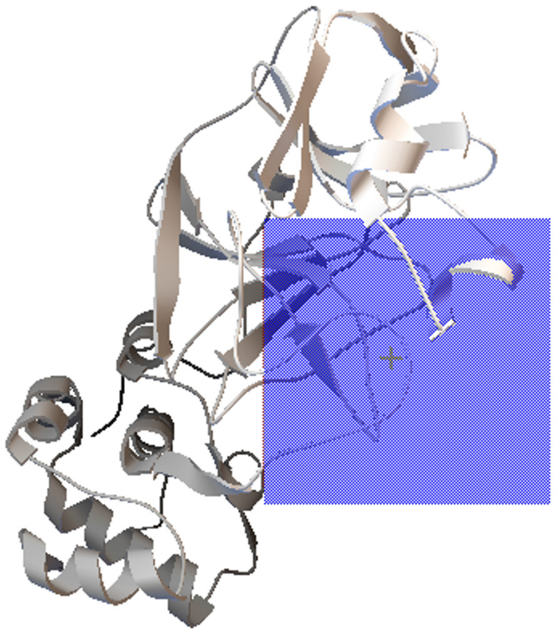

Figure S1. The employed grid box in Autodock computations.

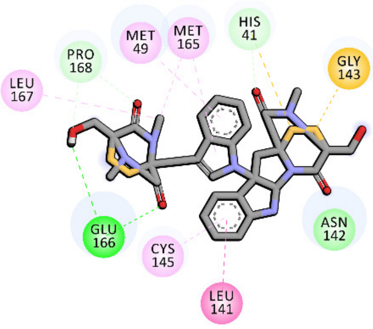

UMHMNP1403367

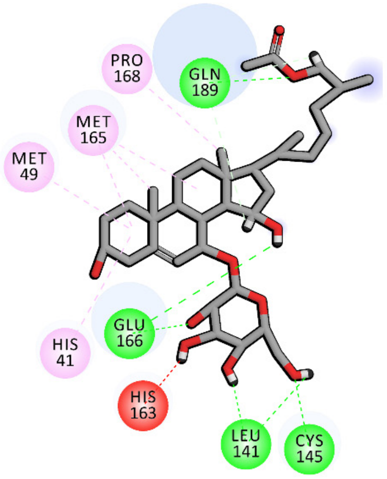

UMHMNP101691127

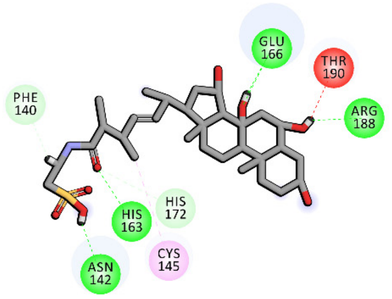

UMHMNP791849666

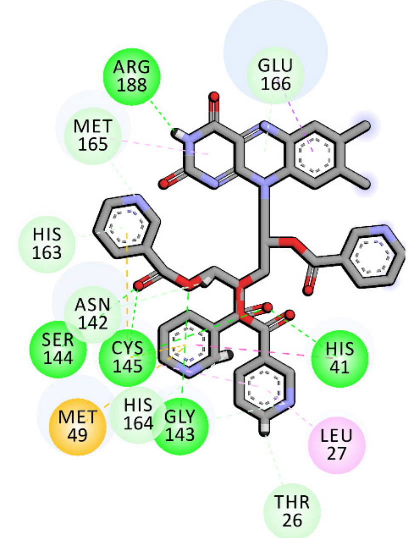

UMHMNP14984668

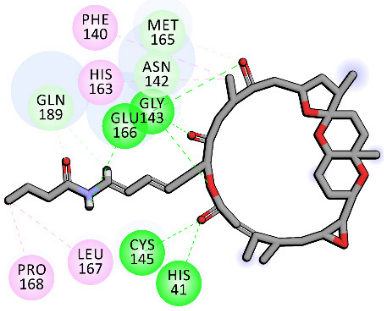

UMHMNP143621754

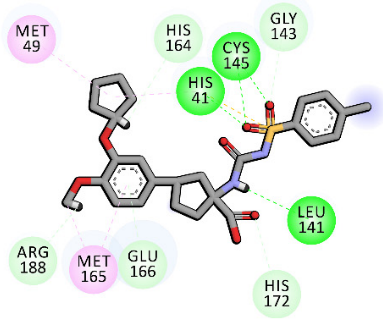

UMHMNP148839036

Figure S2. 2D representation of binding modes and interactions for top thirteen scoring drugs with the key amino acid residues of the 3CL^{pro} binding pocket.

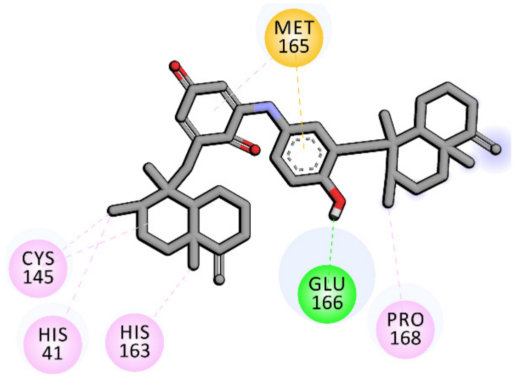

UMHMNP133056072

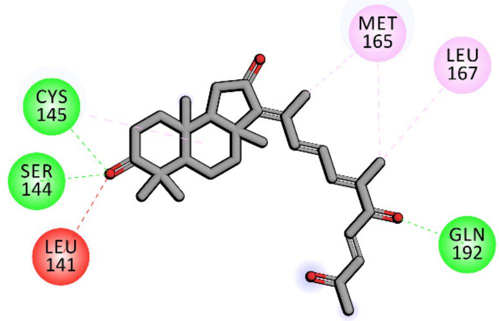

UMHMNP386274857

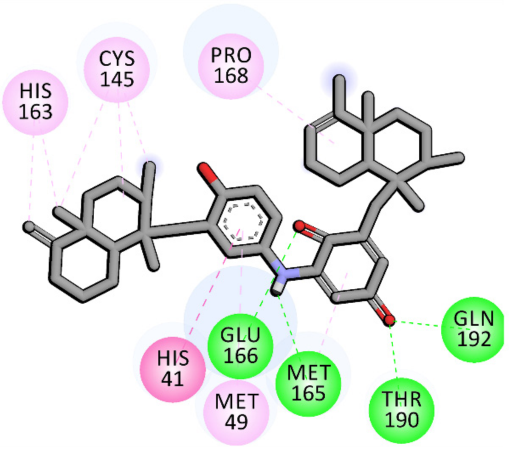

UMHMNP133056094

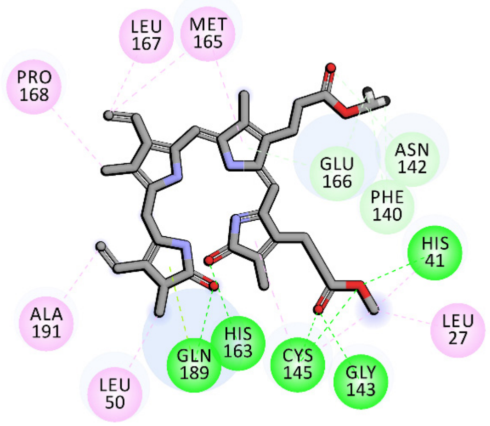

UMHMNP26195584

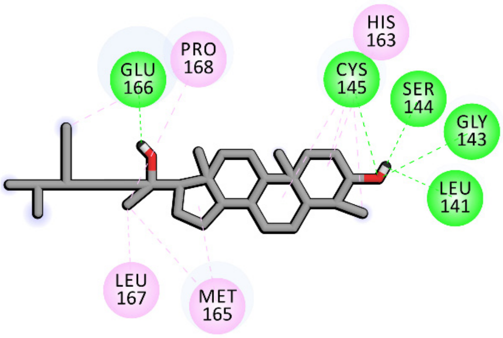

UMHMNP874383707

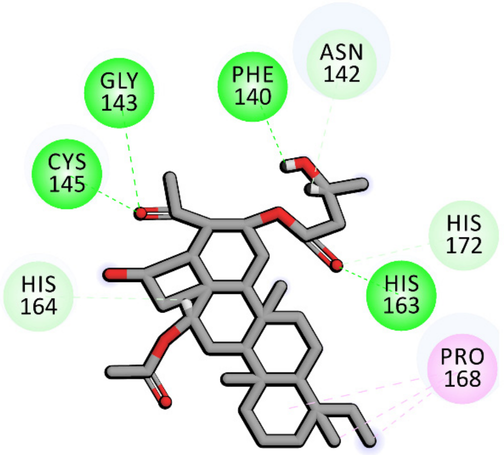

UMHMNP221163300

Figure S2. *Continued.*

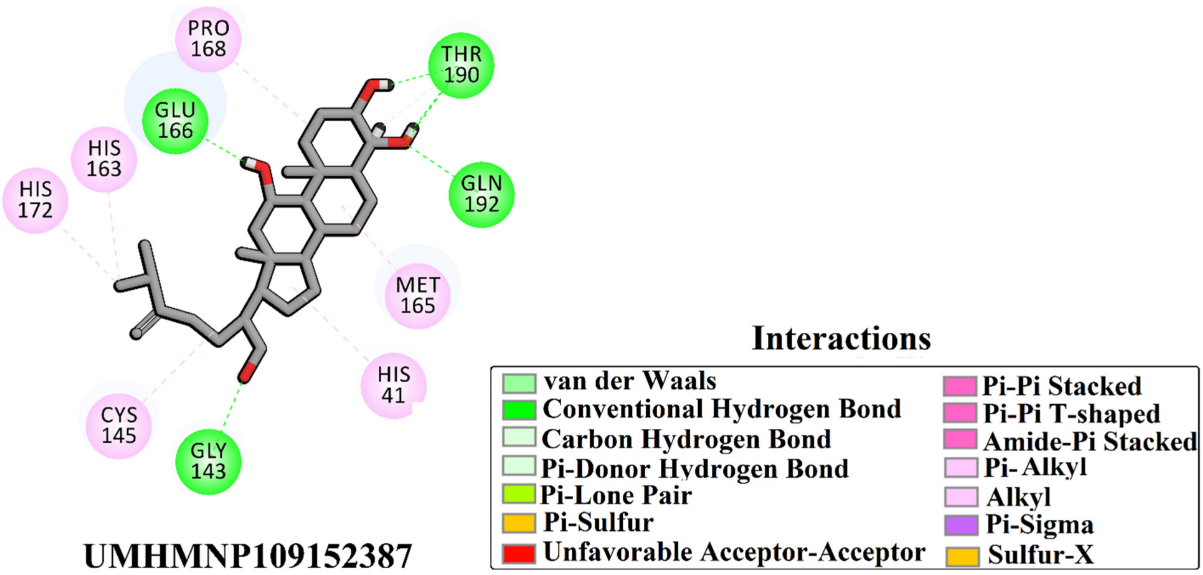

Figure S2. *Continued.*

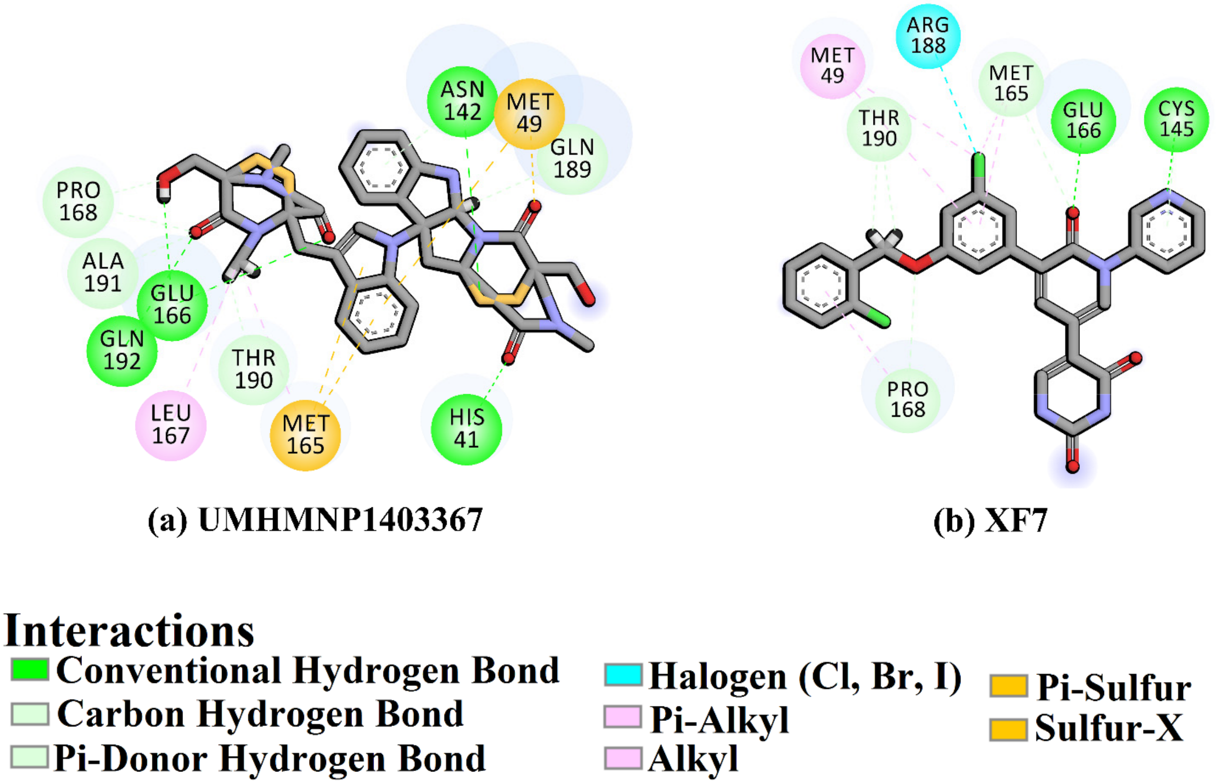

Figure S3. 2D molecular interaction pattern of binding modes of (a) UMHMNP1403367 and (b) XF7 complexed with $3CL^{pro}$ based on the average structure over a 200 ns MD simulation

Table S1. Estimated fast and moderate docking scores (in kcal/mol) for top 2686 MNPs towards 3CL^{pro}.[a]

No.	Compound Name/Code	Docking Score (kcal/mol)		No.	Compound Name/Code	Docking Score (kcal/mol)		No.	Compound Name/Code	Docking Score (kcal/mol)	
		**Fast**	**Mod.**			**Fast**	**Mod.**			**Fast**	**Mod.**
	XF7	−8.1	−9.2								
1	UMHMNP1403367	−11.7	−12.2	53	UMHMNP171674923	−8.9	−10.8	105	UMHMNP253195403	−8.6	−10.4
2	UMHMNP101691127	−11.4	−12.1	54	UMHMNP151484785	−9.2	−10.8	106	UMHMNP336825311	−9.4	−10.4
3	UMHMNP791849666	−11.4	−11.9	55	UMHMNP125035838	−9.2	−10.7	107	UMHMNP99528877	−10.4	−10.4
4	UMHMNP14984668	−11.3	−11.7	56	UMHMNP145400573	−10.7	−10.7	108	UMHMNP112663920	−10.4	−10.4
5	UMHMNP143621754	−11.3	−11.7	57	UMHMNP139975589	−9.9	−10.7	109	UMHMNP80981379	−10.4	−10.4
6	UMHMNP148839036	−11.3	−11.6	58	UMHMNP272458330	−10.6	−10.7	110	UMHMNP552839802	−8.1	−10.4
7	UMHMNP133056072	−11.3	−11.6	59	UMHMNP272458341	−8.6	−10.7	111	UMHMNP142755133	−9.8	−10.4
8	UMHMNP386274857	−11.3	−11.6	60	UMHMNP86708329	−10.6	−10.7	112	UMHMNP131727010	−10.8	−10.4
9	UMHMNP133056094	−11.2	−11.5	61	UMHMNP122143902	−8.9	−10.7	113	UMHMNP142796314	−10.3	−10.4
10	UMHMNP26195584	−11.1	−11.5	62	UMHMNP61369275	−8.3	−10.7	114	UMHMNP374819086	−8.0	−10.4
11	UMHMNP874383707	−11.1	−11.5	63	UMHMNP176666834	−9.0	−10.7	115	UMHMNP105404840	−9.2	−10.4
12	UMHMNP221163300	−11.1	−11.4	64	UMHMNP151078921	−8.8	−10.7	116	UMHMNP112953114	−10.9	−10.4
13	UMHMNP109152387	−11.1	−11.4	65	UMHMNP139765370	−8.1	−10.7	117	UMHMNP147395979	−8.1	−10.4
14	UMHMNP156953912	−11.1	−11.4	66	UMHMNP2497747	−10.7	−10.7	118	UMHMNP156953934	−9.5	−10.4
15	UMHMNP5035303	−11.0	−11.4	67	UMHMNP70139569	−10.7	−10.7	119	UMHMNP173792580	−9.9	−10.4
16	UMHMNP706784727	−10.9	−11.3	68	UMHMNP244157946	−10.6	−10.7	120	UMHMNP454476870	−9.6	−10.4
17	UMHMNP100942743	−8.8	−11.2	69	UMHMNP64421201	−9.5	−10.7	121	UMHMNP152845744	−9.5	−10.4
18	UMHMNP125398290	−8.3	−11.2	70	UMHMNP100942754	−9.1	−10.7	122	UMHMNP77944037	−10.1	−10.4
19	UMHMNP147362376	−8.4	−11.2	71	UMHMNP41410586	−8.8	−10.7	123	UMHMNP116477237	−9.2	−10.3
20	UMHMNP149444928	−8.2	−11.2	72	UMHMNP64548169	−9.9	−10.7	124	UMHMNP133613771	−9.8	−10.3
21	UMHMNP149764341	−9.7	−11.2	73	UMHMNP162830231	−8.9	−10.6	125	UMHMNP516869	−8.4	−10.3
22	UMHMNP150050138	−9.9	−11.2	74	UMHMNP351443759	−8.4	−10.6	126	UMHMNP97877695	−10.3	−10.3
23	UMHMNP151484694	−9.3	−11.2	75	UMHMNP852872925	−9.7	−10.6	127	UMHMNP623167715	−9.8	−10.3
24	UMHMNP153212852	−8.4	−11.2	76	UMHMNP152110093	−8.1	−10.6	128	UMHMNP151109545	−8.9	−10.3
25	UMHMNP157207915	−8.3	−11.2	77	UMHMNP159903670	−8.4	−10.6	129	UMHMNP152247922	−8.6	−10.3
26	UMHMNP157622601	−9.5	−11.2	78	UMHMNP74185049	−9.6	−10.6	130	UMHMNP19698669	−9.5	−10.3
27	UMHMNP167172987	−8.1	−11.2	79	UMHMNP161470231	−8.9	−10.6	131	UMHMNP6901606	−10.3	−10.3
28	UMHMNP184885890	−9.5	−11.2	80	UMHMNP165815816	−8.5	−10.6	132	UMHMNP890041675	−9.7	−10.3
29	UMHMNP22033870	−8.7	−11.2	81	UMHMNP143049165	−8.9	−10.6	133	UMHMNP116302386	−10.4	−10.3
30	UMHMNP224577348	−10.8	−11.2	82	UMHMNP124689652	−9.0	−10.5	134	UMHMNP131703156	−10.9	−10.3
31	UMHMNP5035304	−8.7	−11.2	83	UMHMNP22149695	−9.4	−10.5	135	UMHMNP117631502	−8.5	−10.3
32	UMHMNP76915247	−10.9	−11.2	84	UMHMNP129350181	−8.2	−10.5	136	UMHMNP142796303	−10.2	−10.3
33	UMHMNP823808555	−10.9	−11.2	85	UMHMNP153209111	−8.6	−10.5	137	UMHMNP175992991	−10.0	−10.3
34	UMHMNP120685112	−8.5	−11.2	86	UMHMNP110189043	−9.5	−10.5	138	UMHMNP186593839	−9.1	−10.3
35	UMHMNP162830264	−8.3	−11.2	87	UMHMNP244157957	−10.4	−10.5	139	UMHMNP21059154	−8.3	−10.3
36	UMHMNP153212863	−10.6	−11.1	88	UMHMNP77982800	−10.4	−10.5	140	UMHMNP126149904	−8.8	−10.3
37	UMHMNP154205182	−8.8	−11.1	89	UMHMNP150079951	−10.9	−10.5	141	UMHMNP140715851	−10.3	−10.3
38	UMHMNP174630070	−10.9	−11.0	90	UMHMNP175669279	−8.9	−10.5	142	UMHMNP142755144	−9.5	−10.3
39	UMHMNP94203539	−9.9	−11.0	91	UMHMNP54369118	−8.9	−10.5	143	UMHMNP161470253	−10.0	−10.3
40	UMHMNP116407160	−8.2	−11.0	92	UMHMNP566943746	−9.1	−10.5	144	UMHMNP870535129	−9.9	−10.3
41	UMHMNP106231258	−10.0	−11.0	93	UMHMNP868395884	−10.2	−10.5	145	UMHMNP97614647	−10.1	−10.3
42	UMHMNP162465807	−9.7	−11.0	94	UMHMNP221163311	−9.6	−10.5	`146	UMHMNP91283919	−10.1	−10.3
43	UMHMNP58670636	−9.5	−11.0	95	UMHMNP70329281	−10.1	−10.5	147	UMHMNP196403926	−8.2	−10.3
44	UMHMNP86748314	−10.8	−10.9	96	UMHMNP18097671	−9.6	−10.5	148	UMHMNP23518981	−8.6	−10.3
45	UMHMNP154466372	−9.5	−10.9	97	UMHMNP290824474	−8.8	−10.5	149	UMHMNP2418453	−10.0	−10.3
46	UMHMNP116303	−8.4	−10.9	98	UMHMNP474395	−10.2	−10.5	150	UMHMNP386274868	−8.9	−10.3
47	UMHMNP890041722	−10.8	−10.9	99	UMHMNP741259858	−10.1	−10.5	151	UMHMNP29782658	−8.8	−10.3
48	UMHMNP664992272	−9.0	−10.9	100	UMHMNP6673683	−10.3	−10.5	152	UMHMNP54028465	−9.0	−10.3
49	UMHMNP6673661	−9.6	−10.9	101	UMHMNP827348072	−10.2	−10.5	153	UMHMNP61843796	−8.5	−10.3
50	UMHMNP444987637	−8.8	−10.8	102	UMHMNP107503093	−10.0	−10.5	154	UMHMNP888041267	−9.3	−10.2
51	UMHMNP131467000	−10.7	−10.8	103	UMHMNP132310908	−9.3	−10.5	155	UMHMNP152130615	−10.5	−10.2
52	UMHMNP757976673	−10.2	−10.8	104	UMHMNP126622648	−8.1	−10.4	156	UMHMNP170894367	−9.3	−10.2

Table S1. *Continued.*

No.	Compound Name/Code	Docking Score (kcal/mol)		No.	Compound Name/Code	Docking Score (kcal/mol)		No.	Compound Name/Code	Docking Score (kcal/mol)	
		Fast	Mod.			Fast	Mod.			Fast	Mod.
157	UMHMNP6377180	−9.8	−10.2	209	UMHMNP97649042	−10.1	−10.1	261	UMHMNP136781958	−9.0	−10.0
158	UMHMNP72509616	−10.2	−10.2	210	UMHMNP102488584	−10.1	−10.1	262	UMHMNP139765347	−9.7	−10.0
159	UMHMNP870095344	−9.0	−10.2	211	UMHMNP1058613	−9.9	−10.1	263	UMHMNP26195584	−8.7	−10.0
160	UMHMNP82866339	−8.5	−10.2	212	UMHMNP29939977	−8.6	−10.1	264	UMHMNP333723981	−8.7	−10.0
161	UMHMNP862286702	−8.9	−10.2	213	UMHMNP55688432	−9.5	−10.1	265	UMHMNP57874	−8.4	−10.0
162	UMHMNP116972931	−8.3	−10.2	214	UMHMNP676271833	−9.1	−10.1	266	UMHMNP115268434	−9.0	−10.0
163	UMHMNP22260464	−9.3	−10.2	215	UMHMNP72007944	−9.9	−10.1	267	UMHMNP119147125	−8.1	−10.0
164	UMHMNP682808895	−9.6	−10.2	216	UMHMNP81826495	−8.4	−10.1	268	UMHMNP214899215	−8.1	−10.0
165	UMHMNP149355759	−8.1	−10.2	217	UMHMNP858950400	−10.1	−10.1	269	UMHMNP233607711	−9.8	−10.0
166	UMHMNP85733795	−10.1	−10.2	218	UMHMNP91683328	−9.5	−10.1	270	UMHMNP23670942	−10.0	−10.0
167	UMHMNP109152376	−10.1	−10.2	219	UMHMNP94935974	−10.0	−10.1	271	UMHMNP28949668	−9.7	−10.0
168	UMHMNP53216027	−9.2	−10.2	220	UMHMNP102396178	−10.1	−10.1	272	UMHMNP83115440	−9.9	−10.0
169	UMHMNP113592817	−8.9	−10.2	221	UMHMNP134131183	−9.9	−10.1	273	UMHMNP874193610	−9.9	−10.0
170	UMHMNP118574735	−8.3	−10.2	222	UMHMNP140715873	−10.0	−10.1	274	UMHMNP150050127	−8.4	−10.0
171	UMHMNP681227032	−9.8	−10.2	223	UMHMNP151247697	−8.0	−10.1	275	UMHMNP244157980	−8.4	−10.0
172	UMHMNP88191144	−8.8	−10.2	224	UMHMNP253340293	−8.2	−10.1	276	UMHMNP27065958	−8.5	−10.0
173	UMHMNP143380396	−10.1	−10.2	225	UMHMNP133562499	−9.2	−10.1	277	UMHMNP73792935	−9.9	−10.0
174	UMHMNP168569184	−10.9	−10.2	226	UMHMNP189083781	−8.7	−10.1	278	UMHMNP136762942	−9.4	−10.0
175	UMHMNP174630116	−10.3	−10.2	227	UMHMNP50909869	−8.1	−10.1	279	UMHMNP185014599	−8.3	−10.0
176	UMHMNP40446068	−8.7	−10.2	228	UMHMNP69081885	−9.1	−10.1	280	UMHMNP681260093	−9.7	−10.0
177	UMHMNP405095945	−8.7	−10.2	229	UMHMNP80925060	−9.3	−10.1	281	UMHMNP852469375	−9.8	−10.0
178	UMHMNP6891356	−10.1	−10.2	230	UMHMNP137576002	−9.9	−10.0	282	UMHMNP126297390	−9.0	−9.9
179	UMHMNP84025321	−9.4	−10.2	231	UMHMNP54854921	−8.2	−10.0	283	UMHMNP188112858	−8.1	−9.9
180	UMHMNP19254694	−8.9	−10.2	232	UMHMNP80375184	−9.9	−10.0	279	UMHMNP283176136	−8.8	−9.9
181	UMHMNP114127785	−8.9	−10.1	233	UMHMNP83681792	−9.7	−10.0	284	UMHMNP51744559	−8.5	−9.9
182	UMHMNP244157968	−10.2	−10.1	234	UMHMNP57800585	−9.5	−10.0	285	UMHMNP53296836	−9.8	−9.9
183	UMHMNP481141	−8.8	−10.1	235	UMHMNP832723736	−9.6	−10.0	286	UMHMNP121678873	−9.7	−9.9
184	UMHMNP154496874	−10.9	−10.1	236	UMHMNP866025629	−8.6	−10.0	287	UMHMNP135091111	−8.4	−9.9
185	UMHMNP166797746	−8.3	−10.1	237	UMHMNP53822983	−8.5	−10.0	288	UMHMNP139975578	−8.3	−9.9
186	UMHMNP74185129	−8.8	−10.1	238	UMHMNP56525736	−8.7	−10.0	289	UMHMNP143572730	−10.5	−9.9
187	UMHMNP83115473	−9.2	−10.1	239	UMHMNP78342388	−9.4	−10.0	290	UMHMNP151890812	−8.7	−9.9
188	UMHMNP104048171	−9.9	−10.1	240	UMHMNP105064300	−9.5	−10.0	291	UMHMNP213547285	−8.8	−9.9
189	UMHMNP165815849	−8.5	−10.1	241	UMHMNP123165800	−8.1	−10.0	292	UMHMNP32352651	−9.4	−9.9
190	UMHMNP58028232	−8.4	−10.1	242	UMHMNP75605855	−9.8	−10.0	293	UMHMNP3903579	−8.2	−9.9
191	UMHMNP201800615	−8.7	−10.1	243	UMHMNP76094569	−10.0	−10.0	294	UMHMNP851610907	−10.0	−9.9
192	UMHMNP217449199	−8.1	−10.1	244	UMHMNP84223030	−9.7	−10.0	295	UMHMNP123158907	−9.6	−9.9
193	UMHMNP83704103	−8.8	−10.1	245	UMHMNP854203784	−9.9	−10.0	296	UMHMNP150931832	−8.3	−9.9
194	UMHMNP876620245	−9.8	−10.1	246	UMHMNP99617386	−10.0	−10.0	297	UMHMNP871943985	−9.8	−9.9
195	UMHMNP94451215	−9.9	−10.1	247	UMHMNP129623309	−8.4	−10.0	298	UMHMNP102054455	−9.5	−9.9
196	UMHMNP104928305	−10.0	−10.1	248	UMHMNP134128168	−9.5	−10.0	299	UMHMNP143592249	−8.4	−9.9
197	UMHMNP123314216	−8.4	−10.1	249	UMHMNP4298004	−8.8	−10.0	300	UMHMNP199600390	−8.6	−9.9
198	UMHMNP244157979	−10.0	−10.1	250	UMHMNP53296847	−8.3	−10.0	301	UMHMNP2122987	−8.7	−9.9
199	UMHMNP260436279	−8.1	−10.1	251	UMHMNP876756568	−9.9	−10.0	302	UMHMNP479411455	−8.5	−9.9
200	UMHMNP393870332	−9.1	−10.1	252	UMHMNP90108637	−8.9	−10.0	303	UMHMNP62410962	−8.2	−9.9
201	UMHMNP50335030	−10.1	−10.1	253	UMHMNP224576027	−9.2	−10.0	304	UMHMNP693790748	−9.8	−9.9
202	UMHMNP196500784	−9.2	−10.1	254	UMHMNP25747448	−9.7	−10.0	305	UMHMNP753457942	−9.8	−9.9
203	UMHMNP211358666	−9.1	−10.1	255	UMHMNP858950444	−10.0	−10.0	306	UMHMNP85733762	−9.8	−9.9
204	UMHMNP221169762	−8.6	−10.1	256	UMHMNP94806049	−9.9	−10.0	307	UMHMNP14050625	−9.4	−9.9
205	UMHMNP57082367	−9.1	−10.1	257	UMHMNP110012185	−9.9	−10.0	308	UMHMNP160098931	−8.5	−9.9
206	UMHMNP63109193	−9.0	−10.1	258	UMHMNP117675191	−9.0	−10.0	309	UMHMNP200942183	−8.3	−9.9
207	UMHMNP735278281	−10.0	−10.1	259	UMHMNP128229641	−8.6	−10.0	310	UMHMNP34212936	−8.3	−9.9
208	UMHMNP93426905	−10.1	−10.1	260	UMHMNP133883224	−9.8	−10.0	311	UMHMNP474624	−9.8	−9.9

Table S1. *Continued.*

No.	Compound Name/Code	Docking Score (kcal/mol)		No.	Compound Name/Code	Docking Score (kcal/mol)		No.	Compound Name/Code	Docking Score (kcal/mol)	
		Fast	Mod.			Fast	Mod.			Fast	Mod.
312	UMHMNP810685564	−9.3	−9.9	364	UMHMNP133738484	−9.8	−9.8	416	UMHMNP75197385	−9.5	−9.8
313	UMHMNP97564948	−8.6	−9.9	365	UMHMNP188111708	−8.3	−9.8	417	UMHMNP752211908	−9.7	−9.8
314	UMHMNP103065209	−9.5	−9.9	366	UMHMNP80735064	−8.7	−9.8	418	UMHMNP771534362	−9.7	−9.8
315	UMHMNP125329091	−10.9	−9.9	367	UMHMNP106534445	−8.9	−9.8	419	UMHMNP866403716	−9.6	−9.8
316	UMHMNP142755155	−9.8	−9.9	368	UMHMNP168569151	−8.3	−9.8	420	UMHMNP96363063	−9.3	−9.8
317	UMHMNP151564022	−10.7	−9.9	369	UMHMNP233607697	−9.7	−9.8	421	UMHMNP97190313	−8.8	−9.8
318	UMHMNP168004851	−8.5	−9.9	370	UMHMNP720681630	−9.8	−9.8	422	UMHMNP104196681	−9.7	−9.8
319	UMHMNP208927155	−9.4	−9.9	371	UMHMNP97889560	−9.7	−9.8	423	UMHMNP1247649	−8.5	−9.8
320	UMHMNP211358655	−8.6	−9.9	372	UMHMNP477935969	−9.7	−9.8	424	UMHMNP134887266	−9.7	−9.8
321	UMHMNP272118066	−9.6	−9.9	373	UMHMNP493889	−9.7	−9.8	425	UMHMNP18099246	−8.9	−9.8
322	UMHMNP329050228	−8.5	−9.9	374	UMHMNP78285844	−8.8	−9.8	426	UMHMNP334491257	−8.4	−9.8
323	UMHMNP329050251	−8.1	−9.9	375	UMHMNP123332876	−8.3	−9.8	427	UMHMNP102054466	−8.3	−9.7
324	UMHMNP870535118	−9.8	−9.9	376	UMHMNP149260800	−9.1	−9.8	428	UMHMNP112455842	−9.7	−9.7
325	UMHMNP114216909	−9.2	−9.9	377	UMHMNP168075147	−8.2	−9.8	429	UMHMNP116079539	−8.9	−9.7
326	UMHMNP136762997	−9.7	−9.9	378	UMHMNP172377503	−9.7	−9.8	430	UMHMNP122548032	−8.8	−9.7
327	UMHMNP264624397	−9.8	−9.9	379	UMHMNP185331946	−9.5	−9.8	431	UMHMNP153209122	−8.3	−9.7
328	UMHMNP334491279	−9.3	−9.9	380	UMHMNP2140467	−9.2	−9.8	432	UMHMNP219832378	−8.6	−9.7
329	UMHMNP38636509	−8.7	−9.9	381	UMHMNP4030926	−8.2	−9.8	433	UMHMNP823804837	−9.7	−9.7
330	UMHMNP5371540	−9.3	−9.9	382	UMHMNP102054477	−9.5	−9.8	434	UMHMNP852872936	−8.4	−9.7
331	UMHMNP125127573	−8.3	−9.9	383	UMHMNP144686420	−9.7	−9.8	435	UMHMNP85733739	−8.4	−9.7
332	UMHMNP134887277	−9.8	−9.9	384	UMHMNP151890834	−8.5	−9.8	436	UMHMNP102607761	−9.6	−9.7
333	UMHMNP140389497	−8.4	−9.9	385	UMHMNP155656023	−8.9	−9.8	437	UMHMNP142780436	−8.9	−9.7
334	UMHMNP214483155	−8.7	−9.9	386	UMHMNP179523387	−8.6	−9.8	438	UMHMNP149378563	−9.0	−9.7
335	UMHMNP24778516	−9.7	−9.9	387	UMHMNP2364230	−9.7	−9.8	439	UMHMNP186593862	−8.4	−9.7
336	UMHMNP32450252	−8.7	−9.9	388	UMHMNP4736565	−8.7	−9.8	440	UMHMNP20780410	−8.0	−9.7
337	UMHMNP76758219	−9.7	−9.9	389	UMHMNP55544359	−8.5	−9.8	441	UMHMNP229956472	−9.5	−9.7
338	UMHMNP851610861	−9.7	−9.9	390	UMHMNP80388507	−8.2	−9.8	442	UMHMNP63121062	−9.5	−9.7
339	UMHMNP121714747	−9.5	−9.9	391	UMHMNP858950557	−9.8	−9.8	443	UMHMNP693790737	−9.7	−9.7
340	UMHMNP124561202	−9.3	−9.9	392	UMHMNP196500762	−9.3	−9.8	444	UMHMNP82154210	−9.7	−9.7
341	UMHMNP162232395	−8.2	−9.9	393	UMHMNP19716268	−8.1	−9.8	445	UMHMNP88147197	−8.5	−9.7
342	UMHMNP233607722	−9.9	−9.9	394	UMHMNP37976888	−9.1	−9.8	446	UMHMNP105969640	−8.2	−9.7
343	UMHMNP383859607	−8.1	−9.9	395	UMHMNP473030	−8.6	−9.8	447	UMHMNP200625269	−8.6	−9.7
344	UMHMNP640734878	−9.1	−9.9	396	UMHMNP50909858	−9.9	−9.8	448	UMHMNP264624422	−9.4	−9.7
345	UMHMNP90195418	−8.4	−9.9	397	UMHMNP78183296	−9.7	−9.8	449	UMHMNP71242494	−8.4	−9.7
346	UMHMNP135340000	−9.9	−9.9	398	UMHMNP85617750	−8.9	−9.8	450	UMHMNP85733784	−9.7	−9.7
347	UMHMNP171090792	−8.3	−9.9	399	UMHMNP862587186	−9.0	−9.8	451	UMHMNP863919179	−9.3	−9.7
348	UMHMNP264624400	−9.0	−9.9	400	UMHMNP866025630	−8.1	−9.8	452	UMHMNP97888158	−8.7	−9.7
349	UMHMNP306971120	−8.7	−9.9	401	UMHMNP117417626	−9.8	−9.8	453	UMHMNP115982202	−8.7	−9.7
350	UMHMNP847605769	−9.8	−9.9	402	UMHMNP14908168	−8.9	−9.8	454	UMHMNP131487019	−9.1	−9.7
351	UMHMNP886851269	−8.3	−9.9	403	UMHMNP160666546	−9.5	−9.8	455	UMHMNP141754576	−8.8	−9.7
352	UMHMNP89495476	−9.6	−9.9	404	UMHMNP172854772	−8.2	−9.8	456	UMHMNP149415769	−8.4	−9.7
353	UMHMNP114820290	−8.5	−9.8	405	UMHMNP4680379	−8.8	−9.8	457	UMHMNP197381605	−8.8	−9.7
354	UMHMNP150627364	−8.5	−9.8	406	UMHMNP53296665	−9.6	−9.8	458	UMHMNP2137168	−8.7	−9.7
355	UMHMNP151606405	−9.4	−9.8	407	UMHMNP81387839	−9.8	−9.8	459	UMHMNP219832356	−9.7	−9.7
356	UMHMNP224784489	−9.7	−9.8	408	UMHMNP83115484	−9.3	−9.8	460	UMHMNP221169739	−8.8	−9.7
357	UMHMNP55945749	−8.9	−9.8	409	UMHMNP121994512	−8.8	−9.8	461	UMHMNP34751252	−8.2	−9.7
358	UMHMNP56121427	−8.5	−9.8	410	UMHMNP149260742	−9.0	−9.8	462	UMHMNP75917894	−9.6	−9.7
359	UMHMNP73622238	−9.6	−9.8	411	UMHMNP157459240	−8.6	−9.8	463	UMHMNP10530554	−9.7	−9.7
360	UMHMNP76758208	−9.6	−9.8	412	UMHMNP161776767	−9.3	−9.8	464	UMHMNP131711369	−9.7	−9.7
361	UMHMNP78094005	−8.8	−9.8	413	UMHMNP607388874	−8.7	−9.8	465	UMHMNP151606245	−9.3	−9.7
362	UMHMNP858950466	−9.8	−9.8	414	UMHMNP648428324	−9.5	−9.8	466	UMHMNP160666524	−8.1	−9.7
363	UMHMNP108868957	−8.8	−9.8	415	UMHMNP7273349	−9.4	−9.8	467	UMHMNP194148997	−8.8	−9.7

Table S1. *Continued.*

No.	Compound Name/Code	Docking Score (kcal/mol)		No.	Compound Name/Code	Docking Score (kcal/mol)		No.	Compound Name/Code	Docking Score (kcal/mol)	
		Fast	Mod.			Fast	Mod.			Fast	Mod.
468	UMHMNP223271770	−9.3	−9.7	520	UMHMNP169387728	−9.6	−9.6	572	UMHMNP232585158	−8.4	−9.6
469	UMHMNP478364180	−9.6	−9.7	521	UMHMNP204716041	−8.3	−9.6	573	UMHMNP368421356	−9.3	−9.6
470	UMHMNP59048872	−9.1	−9.7	522	UMHMNP290353683	−10.3	−9.6	574	UMHMNP433717385	−8.2	−9.6
471	UMHMNP85748125	−9.5	−9.7	523	UMHMNP459832678	−8.1	−9.6	575	UMHMNP477885384	−9.2	−9.6
472	UMHMNP114550753	−8.6	−9.7	524	UMHMNP474679	−9.2	−9.6	576	UMHMNP96603020	−9.5	−9.6
473	UMHMNP114820278	−9.4	−9.7	525	UMHMNP567726	−9.1	−9.6	577	UMHMNP109152398	−9.0	−9.6
474	UMHMNP171784091	−9.5	−9.7	526	UMHMNP61947942	−8.5	−9.6	578	UMHMNP121714769	−8.7	−9.6
475	UMHMNP287965280	−8.9	−9.7	527	UMHMNP78285866	−9.2	−9.6	579	UMHMNP452934951	−8.9	−9.6
476	UMHMNP681240608	−9.2	−9.7	528	UMHMNP83454	−9.4	−9.6	580	UMHMNP55309687	−8.1	−9.6
477	UMHMNP134981785	−9.6	−9.7	529	UMHMNP86105648	−9.5	−9.6	581	UMHMNP71947643	−8.0	−9.6
478	UMHMNP149764329	−8.5	−9.7	530	UMHMNP113597038	−9.1	−9.6	582	UMHMNP73731245	−9.4	−9.6
479	UMHMNP211239417	−8.4	−9.7	531	UMHMNP148371088	−9.6	−9.6	583	UMHMNP78370840	−8.9	−9.6
480	UMHMNP75795890	−9.7	−9.7	532	UMHMNP148717913	−9.1	−9.6	584	UMHMNP80357999	−9.2	−9.6
481	UMHMNP882524916	−9.5	−9.7	533	UMHMNP173681522	−9.4	−9.6	585	UMHMNP107168570	−9.6	−9.5
482	UMHMNP96886123	−9.4	−9.7	534	UMHMNP2643029	−8.2	−9.6	586	UMHMNP107585013	−9.5	−9.5
483	UMHMNP123158952	−8.9	−9.7	535	UMHMNP92264136	−9.1	−9.6	587	UMHMNP114820245	−9.4	−9.5
484	UMHMNP152110082	−8.5	−9.7	536	UMHMNP100942721	−9.1	−9.6	588	UMHMNP120154963	−8.6	−9.5
485	UMHMNP157459251	−9.8	−9.7	537	UMHMNP115982199	−9.4	−9.6	589	UMHMNP12903305	−8.3	−9.5
486	UMHMNP159813679	−8.2	−9.7	538	UMHMNP141344107	−8.9	−9.6	590	UMHMNP172854783	−9.2	−9.5
487	UMHMNP675106226	−9.1	−9.7	539	UMHMNP141754587	−8.2	−9.6	591	UMHMNP184584405	−9.4	−9.5
488	UMHMNP139765336	−8.8	−9.7	540	UMHMNP26040006	−9.6	−9.6	592	UMHMNP3963379	−8.3	−9.5
489	UMHMNP171864805	−9.4	−9.7	541	UMHMNP306997397	−8.8	−9.6	593	UMHMNP54602072	−9.2	−9.5
490	UMHMNP481254	−9.5	−9.7	542	UMHMNP481174	−8.8	−9.6	594	UMHMNP55688443	−9.0	−9.5
491	UMHMNP62541097	−8.1	−9.7	543	UMHMNP76376324	−9.6	−9.6	595	UMHMNP64907240	−8.2	−9.5
492	UMHMNP705279716	−9.5	−9.7	544	UMHMNP145398603	−8.9	−9.6	596	UMHMNP115982326	−8.8	−9.5
493	UMHMNP83198270	−9.6	−9.7	545	UMHMNP178200936	−8.6	−9.6	597	UMHMNP126622615	−8.7	−9.5
494	UMHMNP83681816	−9.6	−9.7	546	UMHMNP193074496	−8.1	−9.6	598	UMHMNP157521090	−8.6	−9.5
495	UMHMNP94806027	−8.8	−9.7	547	UMHMNP202855036	−8.4	−9.6	599	UMHMNP171784104	−8.5	−9.5
496	UMHMNP138530731	−9.8	−9.6	548	UMHMNP244303773	−9.4	−9.6	600	UMHMNP191112193	−9.6	−9.5
497	UMHMNP191212369	−8.6	−9.6	549	UMHMNP33168773	−8.5	−9.6	601	UMHMNP21317817	−8.5	−9.5
498	UMHMNP197381570	−9.6	−9.6	550	UMHMNP521040	−8.3	−9.6	602	UMHMNP246036862	−8.9	−9.5
499	UMHMNP221300001	−8.1	−9.6	551	UMHMNP87168330	−9.1	−9.6	603	UMHMNP393828336	−8.7	−9.5
500	UMHMNP244066055	−9.5	−9.6	552	UMHMNP97614625	−9.6	−9.6	604	UMHMNP474635	−8.9	−9.5
501	UMHMNP473739401	−9.5	−9.6	553	UMHMNP133530253	−9.4	−9.6	605	UMHMNP61586003	−8.8	−9.5
502	UMHMNP508193315	−9.4	−9.6	554	UMHMNP153444872	−9.1	−9.6	606	UMHMNP70165456	−9.5	−9.5
503	UMHMNP61897903	−9.2	−9.6	555	UMHMNP169387740	−9.6	−9.6	607	UMHMNP76836174	−10.1	−9.5
504	UMHMNP62008042	−8.2	−9.6	556	UMHMNP193008274	−9.4	−9.6	608	UMHMNP862200495	−9.2	−9.5
505	UMHMNP77715867	−9.3	−9.6	557	UMHMNP199012867	−8.1	−9.6	609	UMHMNP87532283	−9.5	−9.5
506	UMHMNP122540276	−8.1	−9.6	558	UMHMNP287964867	−8.9	−9.6	610	UMHMNP116407217	−8.4	−9.5
507	UMHMNP151890845	−8.2	−9.6	559	UMHMNP80677765	−9.1	−9.6	611	UMHMNP14729294	−9.5	−9.5
508	UMHMNP158758419	−9.0	−9.6	560	UMHMNP107168581	−8.9	−9.6	612	UMHMNP156953898	−8.8	−9.5
509	UMHMNP33646785	−9.0	−9.6	561	UMHMNP154563798	−8.1	−9.6	613	UMHMNP160014879	−9.0	−9.5
510	UMHMNP50868503	−8.4	−9.6	562	UMHMNP169565779	−9.2	−9.6	614	UMHMNP175170895	−9.5	−9.5
511	UMHMNP58497293	−9.0	−9.6	563	UMHMNP17752168	−8.4	−9.6	615	UMHMNP211555021	−8.0	−9.5
512	UMHMNP745075245	−9.5	−9.6	564	UMHMNP19383870	−8.8	−9.6	616	UMHMNP94444254	−9.4	−9.5
513	UMHMNP866403738	−9.6	−9.6	565	UMHMNP242467534	−9.6	−9.6	617	UMHMNP119108360	−9.9	−9.5
514	UMHMNP94806050	−8.8	−9.6	566	UMHMNP710324962	−9.4	−9.6	618	UMHMNP145398647	−9.0	−9.5
515	UMHMNP96313950	−9.2	−9.6	567	UMHMNP77794811	−8.3	−9.6	619	UMHMNP157207880	−8.1	−9.5
516	UMHMNP119760848	−8.4	−9.6	568	UMHMNP84873143	−8.9	−9.6	620	UMHMNP178115907	−9.5	−9.5
517	UMHMNP121819690	−8.3	−9.6	569	UMHMNP121295098	−9.7	−9.6	621	UMHMNP22350498	−9.3	−9.5
518	UMHMNP132911456	−8.3	−9.6	570	UMHMNP143545908	−9.2	−9.6	622	UMHMNP224576050	−9.4	−9.5
519	UMHMNP160014880	−9.3	−9.6	571	UMHMNP184885072	−10.9	−9.6	623	UMHMNP246037605	−9.5	−9.5

Table S1. *Continued.*

No.	Compound Name/Code	Docking Score (kcal/mol)		No.	Compound Name/Code	Docking Score (kcal/mol)		No.	Compound Name/Code	Docking Score (kcal/mol)	
		Fast	Mod.			Fast	Mod.			Fast	Mod.
624	UMHMNP452082596	−9.2	−9.5	676	UMHMNP82507253	−9.4	−9.5	728	UMHMNP17696694	−9.2	−9.4
625	UMHMNP53282681	−9.7	−9.5	677	UMHMNP83511846	−9.0	−9.5	729	UMHMNP195259826	−8.3	−9.4
626	UMHMNP82507264	−8.5	−9.5	678	UMHMNP114820267	−8.7	−9.5	730	UMHMNP209167259	−8.9	−9.4
627	UMHMNP97888147	−8.8	−9.5	679	UMHMNP134128157	−10.0	−9.5	731	UMHMNP214483235	−10.0	−9.4
628	UMHMNP109152332	−9.3	−9.5	680	UMHMNP20465901	−8.3	−9.5	732	UMHMNP570898	−8.6	−9.4
629	UMHMNP15146619	−8.9	−9.5	681	UMHMNP23869161	−8.6	−9.5	733	UMHMNP94806038	−9.2	−9.4
630	UMHMNP639512199	−8.8	−9.5	682	UMHMNP243668124	−9.0	−9.5	734	UMHMNP103005205	−9.2	−9.4
631	UMHMNP66082288	−9.5	−9.5	683	UMHMNP26808275	−8.8	−9.5	735	UMHMNP141266060	−8.4	−9.4
632	UMHMNP79703256	−8.5	−9.5	684	UMHMNP58701705	−8.2	−9.5	736	UMHMNP145176858	−9.1	−9.4
633	UMHMNP864514590	−9.5	−9.5	685	UMHMNP61737971	−9.3	−9.5	737	UMHMNP193008263	−8.9	−9.4
634	UMHMNP108675634	−8.0	−9.5	686	UMHMNP76343947	−9.4	−9.5	738	UMHMNP39832310	−9.0	−9.4
635	UMHMNP125282135	−9.8	−9.5	687	UMHMNP80525491	−9.4	−9.5	739	UMHMNP63109171	−9.3	−9.4
636	UMHMNP129829909	−8.2	−9.5	688	UMHMNP81387840	−9.4	−9.5	740	UMHMNP81262966	−9.9	−9.4
637	UMHMNP160632280	−8.2	−9.5	689	UMHMNP85748136	−9.2	−9.5	741	UMHMNP861676575	−9.4	−9.4
638	UMHMNP183593706	−8.8	−9.5	690	UMHMNP149064346	−9.5	−9.4	742	UMHMNP87697992	−8.9	−9.4
639	UMHMNP226716945	−8.4	−9.5	691	UMHMNP150417677	−8.3	−9.4	743	UMHMNP110189065	−8.2	−9.4
640	UMHMNP272458318	−8.6	−9.5	692	UMHMNP172173987	−8.4	−9.4	744	UMHMNP114820256	−9.5	−9.4
641	UMHMNP298196744	−9.8	−9.5	693	UMHMNP180603672	−9.3	−9.4	745	UMHMNP114836872	−8.6	−9.4
642	UMHMNP321847103	−8.2	−9.5	694	UMHMNP196500773	−9.4	−9.4	746	UMHMNP132922873	−9.3	−9.4
643	UMHMNP80358005	−9.2	−9.5	695	UMHMNP264254866	−9.5	−9.4	747	UMHMNP133562524	−8.4	−9.4
644	UMHMNP81306583	−8.5	−9.5	696	UMHMNP41083970	−8.3	−9.4	748	UMHMNP157758742	−10.4	−9.4
645	UMHMNP185331957	−8.7	−9.5	697	UMHMNP76758184	−8.6	−9.4	749	UMHMNP175861800	−9.3	−9.4
646	UMHMNP313042	−8.7	−9.5	698	UMHMNP771534384	−9.4	−9.4	750	UMHMNP189457269	−8.9	−9.4
647	UMHMNP37299084	−8.3	−9.5	699	UMHMNP78418458	−9.0	−9.4	751	UMHMNP34427639	−8.6	−9.4
648	UMHMNP516370	−9.1	−9.5	700	UMHMNP105418777	−9.4	−9.4	752	UMHMNP74799556	−9.4	−9.4
649	UMHMNP52645097	−8.1	−9.5	701	UMHMNP134887255	−8.1	−9.4	753	UMHMNP76898481	−9.2	−9.4
650	UMHMNP57830	−10.0	−9.5	702	UMHMNP168482346	−8.4	−9.4	754	UMHMNP80981664	−9.2	−9.4
651	UMHMNP59176624	−8.8	−9.5	703	UMHMNP215245264	−9.0	−9.4	755	UMHMNP82637056	−9.3	−9.4
652	UMHMNP745075267	−9.4	−9.5	704	UMHMNP337379198	−9.0	−9.4	756	UMHMNP84582627	−9.5	−9.4
653	UMHMNP89411314	−9.5	−9.5	705	UMHMNP55688501	−8.8	−9.4	757	UMHMNP88191064	−9.4	−9.4
654	UMHMNP144398550	−9.5	−9.5	706	UMHMNP651489	−9.4	−9.4	758	UMHMNP103190143	−9.7	−9.4
655	UMHMNP181306741	−8.5	−9.5	707	UMHMNP681240631	−9.1	−9.4	759	UMHMNP114820303	−8.9	−9.4
656	UMHMNP2061645	−8.4	−9.5	708	UMHMNP81275823	−9.4	−9.4	760	UMHMNP134985072	−9.4	−9.4
657	UMHMNP221169751	−10.0	−9.5	709	UMHMNP124596607	−9.0	−9.4	761	UMHMNP140429374	−9.7	−9.4
658	UMHMNP287964856	−9.6	−9.5	710	UMHMNP145038579	−9.4	−9.4	762	UMHMNP144436077	−10.0	−9.4
659	UMHMNP517900582	−9.1	−9.5	711	UMHMNP174286227	−8.5	−9.4	763	UMHMNP194148975	−9.6	−9.4
660	UMHMNP5813650	−8.4	−9.5	712	UMHMNP179936528	−8.2	−9.4	764	UMHMNP53755092	−9.2	−9.4
661	UMHMNP65717886	−8.1	−9.5	713	UMHMNP264624444	−8.4	−9.4	765	UMHMNP77643244	−8.6	−9.4
662	UMHMNP75266250	−9.5	−9.5	714	UMHMNP51630581	−9.0	−9.4	766	UMHMNP78342377	−8.1	−9.4
663	UMHMNP86690144	−9.5	−9.5	715	UMHMNP61973408	−9.5	−9.4	767	UMHMNP83487	−8.8	−9.4
664	UMHMNP86748303	−9.2	−9.5	716	UMHMNP63635438	−9.6	−9.4	768	UMHMNP132214245	−8.3	−9.4
665	UMHMNP102054488	−8.9	−9.5	717	UMHMNP674819466	−9.4	−9.4	769	UMHMNP151484707	−10.9	−9.4
666	UMHMNP119539752	−9.6	−9.5	718	UMHMNP78342402	−8.4	−9.4	770	UMHMNP155645515	−8.4	−9.4
667	UMHMNP133883177	−9.2	−9.5	719	UMHMNP798557989	−9.0	−9.4	771	UMHMNP169564969	−9.0	−9.4
668	UMHMNP135340011	−9.5	−9.5	720	UMHMNP80525479	−9.2	−9.4	772	UMHMNP171440247	−8.4	−9.4
669	UMHMNP143458757	−8.9	−9.5	721	UMHMNP81120743	−9.3	−9.4	773	UMHMNP180633550	−8.1	−9.4
670	UMHMNP144587553	−8.7	−9.5	722	UMHMNP114820289	−10.3	−9.4	774	UMHMNP181034935	−8.4	−9.4
671	UMHMNP158402610	−8.4	−9.5	723	UMHMNP121449943	−8.9	−9.4	775	UMHMNP107900754	−9.4	−9.4
672	UMHMNP165815827	−8.6	−9.5	724	UMHMNP143049154	−9.6	−9.4	776	UMHMNP126596029	−8.2	−9.4
673	UMHMNP681240620	−9.0	−9.5	725	UMHMNP14958668	−9.2	−9.4	777	UMHMNP16826418	−8.3	−9.4
674	UMHMNP72542495	−8.4	−9.5	726	UMHMNP152247944	−9.7	−9.4	778	UMHMNP171114071	−8.8	−9.4
675	UMHMNP75886123	−9.1	−9.5	727	UMHMNP173220978	−8.6	−9.4	779	UMHMNP70214925	−9.4	−9.4

Table S1. *Continued.*

No.	Compound Name/Code	Docking Score (kcal/mol)		No.	Compound Name/Code	Docking Score (kcal/mol)		No.	Compound Name/Code	Docking Score (kcal/mol)	
		Fast	Mod.			Fast	Mod.			Fast	Mod.
780	UMHMNP71678030	−9.3	−9.4	835	UMHMNP152845755	−10.3	−9.3	890	UMHMNP86105659	−8.9	−9.3
781	UMHMNP745075278	−9.4	−9.4	836	UMHMNP153415351	−8.7	−9.3	891	UMHMNP97719783	−9.1	−9.3
782	UMHMNP78355289	−8.9	−9.4	837	UMHMNP158149543	−8.6	−9.3	892	UMHMNP112515432	−9.1	−9.3
783	UMHMNP1174921	−8.3	−9.4	838	UMHMNP199600378	−8.4	−9.3	893	UMHMNP119212281	−8.2	−9.3
784	UMHMNP13963138	−9.3	−9.4	839	UMHMNP223596723	−9.3	−9.3	894	UMHMNP137350948	−8.6	−9.3
785	UMHMNP173681533	−8.9	−9.4	840	UMHMNP614748628	−8.3	−9.3	895	UMHMNP175992980	−9.2	−9.3
786	UMHMNP433717534	−8.7	−9.4	841	UMHMNP75373625	−9.1	−9.3	896	UMHMNP191789783	−8.3	−9.3
787	UMHMNP65773980	−9.3	−9.4	842	UMHMNP83728818	−8.8	−9.3	897	UMHMNP306967759	−9.2	−9.3
788	UMHMNP67314152	−8.9	−9.4	843	UMHMNP85198216	−9.1	−9.3	898	UMHMNP306997422	−8.8	−9.3
789	UMHMNP70022718	−10.0	−9.4	844	UMHMNP86748278	−9.1	−9.3	899	UMHMNP31893353	−9.4	−9.3
790	UMHMNP77983267	−8.8	−9.4	845	UMHMNP93426927	−9.1	−9.3	900	UMHMNP54448725	−8.9	−9.3
791	UMHMNP78285855	−8.6	−9.4	846	UMHMNP136685295	−9.6	−9.3	901	UMHMNP54602141	−8.2	−9.3
792	UMHMNP882693461	−8.1	−9.4	847	UMHMNP143016868	−8.3	−9.3	902	UMHMNP71932064	−9.2	−9.3
793	UMHMNP109152343	−8.5	−9.3	848	UMHMNP146506414	−8.3	−9.3	903	UMHMNP78518748	−9.2	−9.3
794	UMHMNP196500808	−8.7	−9.3	849	UMHMNP37976899	−8.2	−9.3	904	UMHMNP81853830	−8.8	−9.3
795	UMHMNP199600345	−9.1	−9.3	850	UMHMNP99165169	−8.3	−9.3	905	UMHMNP116747401	−9.8	−9.3
796	UMHMNP286433805	−8.7	−9.3	851	UMHMNP155850887	−8.5	−9.3	906	UMHMNP156352657	−8.3	−9.3
797	UMHMNP81657796	−8.7	−9.3	852	UMHMNP158734248	−9.1	−9.3	907	UMHMNP164081009	−8.6	−9.3
798	UMHMNP862288902	−9.3	−9.3	853	UMHMNP169387773	−9.3	−9.3	908	UMHMNP189818493	−8.1	−9.3
799	UMHMNP100667752	−9.4	−9.3	854	UMHMNP179733143	−9.3	−9.3	909	UMHMNP283605458	−9.4	−9.3
800	UMHMNP121819714	−8.4	−9.3	855	UMHMNP260436291	−10.4	−9.3	910	UMHMNP32450263	−9.3	−9.3
801	UMHMNP121825443	−8.1	−9.3	856	UMHMNP263764018	−8.3	−9.3	911	UMHMNP471534	−8.5	−9.3
802	UMHMNP143682171	−9.3	−9.3	857	UMHMNP37772035	−9.3	−9.3	912	UMHMNP60048900	−8.2	−9.3
803	UMHMNP221160845	−9.6	−9.3	858	UMHMNP41083903	−9.2	−9.3	913	UMHMNP601570	−8.1	−9.3
804	UMHMNP250147194	−9.2	−9.3	859	UMHMNP452082609	−9.0	−9.3	914	UMHMNP71885206	−8.2	−9.3
805	UMHMNP38636496	−8.6	−9.3	860	UMHMNP57885	−10.1	−9.3	915	UMHMNP93426916	−9.5	−9.3
806	UMHMNP448264651	−8.6	−9.3	861	UMHMNP69672680	−9.3	−9.3	917	UMHMNP112515443	−8.8	−9.3
807	UMHMNP636599901	−9.3	−9.3	862	UMHMNP749867230	−9.2	−9.3	918	UMHMNP129620253	−8.6	−9.3
808	UMHMNP675106204	−8.5	−9.3	863	UMHMNP85748147	−9.6	−9.3	919	UMHMNP132410356	−8.7	−9.3
809	UMHMNP75479117	−8.8	−9.3	864	UMHMNP90352208	−8.9	−9.3	920	UMHMNP133883213	−8.3	−9.3
810	UMHMNP81657398	−9.2	−9.3	865	UMHMNP112781234	−8.8	−9.3	921	UMHMNP141859974	−9.8	−9.3
811	UMHMNP148270160	−8.1	−9.3	866	UMHMNP112923407	−9.3	−9.3	922	UMHMNP1452295	−8.1	−9.3
812	UMHMNP155944266	−8.1	−9.3	867	UMHMNP124596629	−8.1	−9.3	923	UMHMNP211358713	−9.1	−9.3
813	UMHMNP174796782	−9.4	−9.3	868	UMHMNP132214234	−8.9	−9.3	924	UMHMNP39832309	−8.6	−9.3
814	UMHMNP183381068	−8.7	−9.3	869	UMHMNP132750482	−8.3	−9.3	925	UMHMNP40772121	−8.7	−9.3
815	UMHMNP188968447	−10.0	−9.3	870	UMHMNP160014868	−8.2	−9.3	926	UMHMNP495380477	−8.5	−9.3
816	UMHMNP395070889	−8.3	−9.3	871	UMHMNP179730360	−9.1	−9.3	927	UMHMNP73538575	−8.2	−9.3
817	UMHMNP74055428	−8.3	−9.3	872	UMHMNP247145397	−8.1	−9.3	928	UMHMNP749216464	−8.5	−9.3
818	UMHMNP85505664	−9.2	−9.3	873	UMHMNP26531715	−8.1	−9.3	929	UMHMNP874221893	−8.4	−9.3
819	UMHMNP86105682	−9.2	−9.3	864	UMHMNP90352208	−8.9	−9.3	930	UMHMNP145038580	−9.0	−9.3
820	UMHMNP94354980	−9.3	−9.3	865	UMHMNP112781234	−8.8	−9.3	931	UMHMNP16408102	−9.1	−9.3
821	UMHMNP107551746	−9.3	−9.3	866	UMHMNP112923407	−9.3	−9.3	932	UMHMNP166038284	−8.4	−9.3
822	UMHMNP109979171	−9.3	−9.3	867	UMHMNP124596629	−8.1	−9.3	933	UMHMNP186803327	−8.2	−9.3
823	UMHMNP147391819	−9.2	−9.3	868	UMHMNP132214234	−8.9	−9.3	934	UMHMNP20817929	−8.7	−9.3
824	UMHMNP156472898	−8.8	−9.3	869	UMHMNP132750482	−8.3	−9.3	935	UMHMNP263764041	−9.6	−9.3
825	UMHMNP161776756	−8.7	−9.3	870	UMHMNP160014868	−8.2	−9.3	936	UMHMNP474577	−8.2	−9.3
826	UMHMNP17991672	−9.3	−9.3	871	UMHMNP179730360	−9.1	−9.3	937	UMHMNP566916	−9.1	−9.3
827	UMHMNP2465114	−9.2	−9.3	872	UMHMNP247145397	−8.1	−9.3	938	UMHMNP64180718	−8.1	−9.3
828	UMHMNP735278270	−9.1	−9.3	873	UMHMNP26531715	−8.1	−9.3	939	UMHMNP68520263	−8.2	−9.3
829	UMHMNP782491785	−9.1	−9.3	884	UMHMNP268735575	−9.9	−9.3	940	UMHMNP72534135	−9.6	−9.3
830	UMHMNP106534434	−9.0	−9.3	885	UMHMNP306967760	−8.5	−9.3	941	UMHMNP91297073	−8.8	−9.3
831	UMHMNP117417717	−9.5	−9.3	886	UMHMNP67463790	−8.6	−9.3	942	UMHMNP95062296	−8.4	−9.3
832	UMHMNP1242019	−8.1	−9.3	887	UMHMNP69511202	−9.0	−9.3	943	UMHMNP100994556	−9.0	−9.3
833	UMHMNP14231335	−9.0	−9.3	888	UMHMNP81575066	−8.4	−9.3	944	UMHMNP124843681	−8.2	−9.3
834	UMHMNP144436066	−8.8	−9.3	889	UMHMNP858950386	−9.2	−9.3	945	UMHMNP130246989	−8.2	−9.3

Table S1. *Continued.*

No.	Compound Name/Code	Docking Score (kcal/mol)		No.	Compound Name/Code	Docking Score (kcal/mol)		No.	Compound Name/Code	Docking Score (kcal/mol)	
		Fast	Mod.			Fast	Mod.			Fast	Mod.
946	UMHMNP134458007	−9.0	−9.3	1001	UMHMNP333970131	−8.5	−9.3	1056	UMHMNP116972920	−9.5	−9.3
947	UMHMNP137570435	−8.5	−9.3	1002	UMHMNP452929645	−9.8	−9.3	1057	UMHMNP128802175	−8.7	−9.3
948	UMHMNP140866202	−8.7	−9.3	1003	UMHMNP461441429	−9.2	−9.3	1058	UMHMNP142780367	−9.6	−9.3
949	UMHMNP145212391	−10.7	−9.3	1004	UMHMNP478364226	−10.1	−9.3	1059	UMHMNP517900559	−9.8	−9.3
950	UMHMNP162290417	−8.5	−9.3	1005	UMHMNP50335041	−9.1	−9.3	1060	UMHMNP52043013	−8.1	−9.3
951	UMHMNP175669100	−8.6	−9.3	1006	UMHMNP771534373	−8.6	−9.3	1061	UMHMNP6035627	−8.2	−9.3
952	UMHMNP216770295	−10.0	−9.3	1007	UMHMNP99617397	−8.8	−9.3	1062	UMHMNP67230062	−9.2	−9.3
953	UMHMNP62996741	−8.7	−9.3	1008	UMHMNP116302353	−8.2	−9.3	1063	UMHMNP10525221	−9.7	−9.3
954	UMHMNP75246763	−8.2	−9.3	1009	UMHMNP121727273	−8.6	−9.3	1064	UMHMNP1159279	−8.6	−9.3
955	UMHMNP85733773	−8.5	−9.3	1010	UMHMNP142677121	−8.5	−9.3	1065	UMHMNP143007287	−8.9	−9.3
956	UMHMNP93474136	−8.5	−9.3	1011	UMHMNP174063811	−8.4	−9.3	1066	UMHMNP152833608	−8.8	−9.3
957	UMHMNP984849	−8.8	−9.3	1012	UMHMNP24662951	−8.1	−9.3	1067	UMHMNP161776734	−8.9	−9.3
958	UMHMNP106199819	−10.0	−9.3	1013	UMHMNP64687850	−9.0	−9.3	1068	UMHMNP178179992	−8.1	−9.3
959	UMHMNP112693240	−8.1	−9.3	1014	UMHMNP71031588	−9.1	−9.3	1069	UMHMNP17974786	−8.5	−9.3
960	UMHMNP116169290	−8.2	−9.3	1015	UMHMNP73723383	−9.1	−9.3	1070	UMHMNP225662099	−10.0	−9.3
961	UMHMNP133883202	−9.9	−9.3	1016	UMHMNP81474615	−9.3	−9.3	1071	UMHMNP326794176	−8.8	−9.3
962	UMHMNP144335148	−8.3	−9.3	1017	UMHMNP85337151	−10.0	−9.3	1072	UMHMNP55081395	−10.5	−9.3
963	UMHMNP148010406	−8.1	−9.3	1018	UMHMNP862200473	−10.8	−9.3	1073	UMHMNP58560375	−8.2	−9.3
964	UMHMNP1491776	−8.2	−9.3	1019	UMHMNP128855072	−8.8	−9.3	1074	UMHMNP62861158	−9.5	−9.3
965	UMHMNP157403333	−8.4	−9.3	1020	UMHMNP14599485	−8.7	−9.3	1075	UMHMNP68690926	−10.7	−9.3
966	UMHMNP189514485	−8.1	−9.3	1021	UMHMNP16250616	−8.6	−9.3	1076	UMHMNP69297490	−9.1	−9.3
967	UMHMNP24041665	−9.2	−9.3	1022	UMHMNP175413215	−8.2	−9.3	1077	UMHMNP79580282	−9.3	−9.3
968	UMHMNP487016966	−8.9	−9.3	1023	UMHMNP224577382	−9.8	−9.3	1078	UMHMNP98495364	−9.3	−9.3
969	UMHMNP50611864	−8.7	−9.3	1024	UMHMNP3404226	−9.2	−9.3	1079	UMHMNP99499817	−9.3	−9.3
970	UMHMNP57103216	−9.0	−9.3	1025	UMHMNP52936693	−8.4	−9.3	1080	UMHMNP135729452	−8.5	−9.3
971	UMHMNP690253771	−8.1	−9.3	1026	UMHMNP73700295	−8.2	−9.3	1081	UMHMNP139765290	−8.9	−9.3
972	UMHMNP871483351	−8.1	−9.3	1027	UMHMNP81387851	−9.9	−9.3	1082	UMHMNP143049132	−10.5	−9.3
973	UMHMNP104720152	−8.7	−9.3	1028	UMHMNP850145129	−8.4	−9.3	1083	UMHMNP143049143	−8.4	−9.3
974	UMHMNP14956242	−9.4	−9.3	1029	UMHMNP87307286	−9.1	−9.3	1084	UMHMNP156310188	−8.3	−9.3
975	UMHMNP214483199	−8.2	−9.3	1030	UMHMNP96253600	−8.8	−9.3	1085	UMHMNP175702360	−9.0	−9.3
976	UMHMNP517900515	−9.7	−9.3	1031	UMHMNP102054524	−9.3	−9.3	1086	UMHMNP178494821	−9.3	−9.3
977	UMHMNP549520743	−8.3	−9.3	1032	UMHMNP117675135	−8.7	−9.3	1087	UMHMNP184430011	−9.2	−9.3
978	UMHMNP87859981	−9.2	−9.3	1033	UMHMNP141321133	−8.7	−9.3	1088	UMHMNP38819442	−8.3	−9.3
979	UMHMNP139765303	−8.3	−9.3	1034	UMHMNP141859985	−9.5	−9.3	1089	UMHMNP54622514	−8.5	−9.3
980	UMHMNP149260731	−10.2	−9.3	1035	UMHMNP145349946	−9.0	−9.3	1090	UMHMNP99617433	−9.3	−9.3
981	UMHMNP149260753	−9.4	−9.3	1036	UMHMNP158786755	−9.3	−9.3	1091	UMHMNP117021064	−9.4	−9.3
982	UMHMNP22850119	−8.5	−9.3	1037	UMHMNP178064410	−9.3	−9.3	1092	UMHMNP124727077	−8.3	−9.3
983	UMHMNP237755167	−9.4	−9.3	1038	UMHMNP517900504	−9.2	−9.3	1093	UMHMNP132194322	−9.1	−9.1
984	UMHMNP264618215	−8.3	−9.3	1039	UMHMNP518069	−8.3	−9.3	1094	UMHMNP141854695	−8.2	−9.1
985	UMHMNP38788817	−9.2	−9.3	1040	UMHMNP681226415	−9.7	−9.3	1095	UMHMNP158734271	−8.3	−9.1
986	UMHMNP425368598	−9.0	−9.3	1041	UMHMNP745075256	−8.5	−9.3	1096	UMHMNP171784013	−8.6	−9.1
987	UMHMNP446862697	−8.6	−9.3	1042	UMHMNP80525480	−8.3	−9.3	1097	UMHMNP215245286	−9.6	−9.1
988	UMHMNP454476892	−8.7	−9.3	1043	UMHMNP126394749	−10.4	−9.3	1098	UMHMNP473256223	−8.8	−9.1
989	UMHMNP489446573	−9.1	−9.3	1044	UMHMNP133883199	−9.5	−9.3	1099	UMHMNP479067624	−9.4	−9.1
990	UMHMNP781646819	−8.4	−9.3	1045	UMHMNP143049121	−8.2	−9.3	1100	UMHMNP64997520	−8.9	−9.1
991	UMHMNP863684417	−9.8	−9.3	1046	UMHMNP147362398	−8.6	−9.3	1101	UMHMNP70329258	−8.1	−9.1
992	UMHMNP132938126	−8.3	−9.3	1047	UMHMNP156472887	−11.4	−9.3	1102	UMHMNP724462480	−8.2	−9.1
993	UMHMNP147395093	−8.1	−9.3	1048	UMHMNP199600367	−8.4	−9.3	1103	UMHMNP865308607	−9.9	−9.1
994	UMHMNP147641723	−9.1	−9.3	1049	UMHMNP2243096	−8.1	−9.3	1104	UMHMNP105369899	−8.4	−9.1
995	UMHMNP158822765	−8.5	−9.3	1050	UMHMNP31220447	−8.5	−9.3	1105	UMHMNP117857724	−8.9	−9.1
996	UMHMNP163318783	−8.8	−9.3	1051	UMHMNP494862714	−9.9	−9.3	1106	UMHMNP151890823	−10.3	−9.1
997	UMHMNP17181883	−8.1	−9.3	1052	UMHMNP516858	−9.3	−9.3	1107	UMHMNP153698909	−8.3	−9.1
998	UMHMNP188112825	−8.1	−9.3	1053	UMHMNP74799590	−8.3	−9.3	1108	UMHMNP169217452	−8.8	−9.1
999	UMHMNP209408720	−8.1	−9.3	1054	UMHMNP876756557	−8.9	−9.3	1109	UMHMNP2034722	−8.7	−9.1
1000	UMHMNP209408764	−8.7	−9.3	1055	UMHMNP115193152	−9.6	−9.3	1110	UMHMNP2259907	−9.5	−9.1

Table S1. *Continued.*

No.	Compound Name/Code	Docking Score (kcal/mol)		No.	Compound Name/Code	Docking Score (kcal/mol)		No.	Compound Name/Code	Docking Score (kcal/mol)	
		Fast	Mod.			Fast	Mod.			Fast	Mod.
1111	UMHMNP24041676	−8.9	−9.1	1166	UMHMNP16910320	−9.3	−9.0	1221	UMHMNP694436618	−8.4	−9.0
1112	UMHMNP39533738	−8.8	−9.1	1167	UMHMNP171114059	−9.1	−9.0	1222	UMHMNP80388461	−8.4	−9.0
1113	UMHMNP58514322	−9.8	−9.1	1168	UMHMNP175861822	−8.7	−9.0	1223	UMHMNP876756502	−9.6	−9.0
1114	UMHMNP87164338	−8.3	−9.1	1169	UMHMNP208708230	−8.1	−9.0	1224	UMHMNP115439617	−8.7	−9.0
1115	UMHMNP882400853	−8.3	−9.1	1170	UMHMNP39025279	−8.7	−9.0	1225	UMHMNP12626185	−9.5	−9.0
1116	UMHMNP90195441	−8.5	−9.1	1171	UMHMNP446862722	−8.7	−9.0	1226	UMHMNP140384709	−9.0	−9.0
1117	UMHMNP102054499	−8.5	−9.1	1172	UMHMNP4598678	−9.7	−9.0	1227	UMHMNP143682182	−9.7	−9.0
1118	UMHMNP107551757	−8.4	−9.1	1173	UMHMNP51529125	−8.6	−9.0	1228	UMHMNP149355760	−10.3	−9.0
1119	UMHMNP1106134	−8.2	−9.1	1174	UMHMNP64285854	−8.5	−9.0	1229	UMHMNP162830253	−8.9	−9.0
1120	UMHMNP116302284	−10.4	−9.1	1175	UMHMNP72154337	−8.1	−9.0	1230	UMHMNP175673582	−8.3	−9.0
1121	UMHMNP122540312	−8.7	−9.1	1176	UMHMNP81624035	−9.6	−9.0	1231	UMHMNP209169584	−8.7	−9.0
1122	UMHMNP15313696	−10.9	−9.1	1177	UMHMNP83465	−8.2	−9.0	1232	UMHMNP55688454	−8.4	−9.0
1123	UMHMNP171114093	−9.2	−9.1	1178	UMHMNP85443374	−8.3	−9.0	1233	UMHMNP6758710	−9.6	−9.0
1124	UMHMNP298196733	−8.2	−9.1	1179	UMHMNP89837978	−8.8	−9.0	1234	UMHMNP71801495	−9.1	−9.0
1125	UMHMNP744253483	−9.1	−9.1	1180	UMHMNP128629378	−10.5	−9.0	1235	UMHMNP848132354	−8.2	−9.0
1126	UMHMNP848132376	−8.2	−9.1	1181	UMHMNP129620231	−8.2	−9.0	1236	UMHMNP862522	−9.9	−9.0
1127	UMHMNP88840000	−8.1	−9.1	1182	UMHMNP139765358	−8.4	−9.0	1237	UMHMNP117675168	−9.2	−9.0
1128	UMHMNP96853155	−8.6	−9.1	1183	UMHMNP163136058	−8.6	−9.0	1238	UMHMNP123062431	−8.1	−9.0
1129	UMHMNP121135000	−9.4	−9.1	1184	UMHMNP175861855	−8.2	−9.0	1239	UMHMNP123165797	−8.4	−9.0
1130	UMHMNP123086817	−8.4	−9.1	1185	UMHMNP238758424	−8.9	−9.0	1240	UMHMNP18499859	−8.3	−9.0
1131	UMHMNP133401113	−8.8	−9.1	1186	UMHMNP250141641	−8.1	−9.0	1241	UMHMNP260390781	−8.6	−9.0
1132	UMHMNP165606699	−8.2	−9.1	1187	UMHMNP395070903	−9.9	−9.0	1242	UMHMNP263764052	−8.6	−9.0
1133	UMHMNP185331935	−8.6	−9.1	1188	UMHMNP425368623	−8.8	−9.0	1243	UMHMNP264624411	−8.1	−9.0
1134	UMHMNP188111695	−8.5	−9.1	1189	UMHMNP52936706	−8.7	−9.0	1244	UMHMNP29560267	−9.3	−9.0
1135	UMHMNP260062882	−8.2	−9.1	1190	UMHMNP54278896	−9.5	−9.0	1245	UMHMNP71486049	−9.6	−9.0
1136	UMHMNP33886747	−8.6	−9.1	1191	UMHMNP63015907	−8.5	−9.0	1246	UMHMNP77965812	−8.3	−9.0
1137	UMHMNP495380488	−8.4	−9.1	1192	UMHMNP815586471	−9.6	−9.0	1247	UMHMNP124609387	−9.4	−9.0
1138	UMHMNP516552	−8.1	−9.1	1193	UMHMNP85066780	−8.6	−9.0	1248	UMHMNP133206390	−8.4	−9.0
1139	UMHMNP516790	−9.0	−9.1	1194	UMHMNP85733717	−8.8	−9.0	1249	UMHMNP142508063	−8.1	−9.0
1140	UMHMNP55051780	−9.2	−9.1	1195	UMHMNP96736311	−9.0	−9.0	1250	UMHMNP159934142	−8.5	−9.0
1141	UMHMNP69672704	−8.4	−9.1	1196	UMHMNP134985083	−8.6	−9.0	1251	UMHMNP172173998	−8.5	−9.0
1142	UMHMNP70214992	−9.9	−9.1	1197	UMHMNP145403254	−8.3	−9.0	1252	UMHMNP200860895	−8.8	−9.0
1143	UMHMNP70406_p0	−10.2	−9.1	1198	UMHMNP146556287	−9.0	−9.0	1253	UMHMNP213547252	−8.2	−9.0
1144	UMHMNP80981631	−8.6	−9.1	1199	UMHMNP155233311	−9.1	−9.0	1254	UMHMNP246037854	−8.7	−9.0
1145	UMHMNP815586493	−9.4	−9.1	1200	UMHMNP160098920	−9.4	−9.0	1255	UMHMNP246037898	−8.7	−9.0
1146	UMHMNP85337128	−9.2	−9.1	1201	UMHMNP175861797	−9.2	−9.0	1256	UMHMNP287965202	−8.5	−9.0
1147	UMHMNP85733808	−8.2	−9.1	1202	UMHMNP175861877	−8.4	−9.0	1257	UMHMNP521039	−9.3	−9.0
1148	UMHMNP99631217	−8.8	−9.1	1203	UMHMNP1981904	−8.9	−9.0	1258	UMHMNP58115314	−9.7	−9.0
1149	UMHMNP117694969	−9.9	−9.1	1204	UMHMNP200497047	−8.5	−9.0	1259	UMHMNP61897890	−8.9	−9.0
1150	UMHMNP118984466	−9.6	−9.1	1205	UMHMNP36011195	−8.9	−9.0	1260	UMHMNP69977260	−8.9	−9.0
1151	UMHMNP148471856	−9.0	−9.1	1206	UMHMNP676271855	−10.3	−9.0	1261	UMHMNP89837956	−9.1	−9.0
1152	UMHMNP151345061	−8.5	−9.1	1207	UMHMNP780755375	−8.2	−9.0	1262	UMHMNP1107591	−8.6	−9.0
1153	UMHMNP20512316	−9.0	−9.1	1208	UMHMNP849752870	−8.4	−9.0	1263	UMHMNP135474101	−8.3	−9.0
1154	UMHMNP25819798	−8.2	−9.1	1209	UMHMNP95062310	−8.3	−9.0	1264	UMHMNP143503202	−8.8	−9.0
1155	UMHMNP3090811	−8.2	−9.1	1210	UMHMNP116079540	−8.1	−9.0	1265	UMHMNP190451060	−8.9	−9.0
1156	UMHMNP313503376	−8.5	−9.1	1211	UMHMNP121135011	−8.9	−9.0	1266	UMHMNP200860908	−9.7	−9.0
1157	UMHMNP383859594	−9.1	−9.1	1212	UMHMNP147641712	−8.9	−9.0	1267	UMHMNP20829554	−8.6	−9.0
1158	UMHMNP65228140	−8.5	−9.1	1213	UMHMNP153585649	−8.7	−9.0	1268	UMHMNP212069482	−8.9	−9.0
1159	UMHMNP77521	−9.4	−9.1	1214	UMHMNP157622576	−9.1	−9.0	1269	UMHMNP2326514	−8.8	−9.0
1160	UMHMNP82660615	−8.3	−9.1	1215	UMHMNP167394823	−8.8	−9.0	1270	UMHMNP28032522	−8.8	−9.0
1161	UMHMNP88840022_	−10.1	−9.1	1216	UMHMNP176447964	−8.6	−9.0	1271	UMHMNP52745876	−8.2	−9.0
1162	UMHMNP128364318	−8.3	−9.0	1217	UMHMNP17974775	−9.5	−9.0	1272	UMHMNP74920573	−9.4	−9.0
1163	UMHMNP129602193	−8.2	−9.0	1218	UMHMNP198274258	−9.8	−9.0	1273	UMHMNP749216453	−9.6	−9.0
1164	UMHMNP134515530	−8.1	−9.0	1219	UMHMNP475111785	−9.0	−9.0	1274	UMHMNP751940	−9.2	−9.0
1165	UMHMNP147170089	−8.6	−9.0	1220	UMHMNP54369141	−9.4	−9.0	1275	UMHMNP85482775	−8.3	−9.0

Table S1. *Continued.*

No.	Compound Name/Code	Docking Score (kcal/mol)		No.	Compound Name/Code	Docking Score (kcal/mol)		No.	Compound Name/Code	Docking Score (kcal/mol)	
		Fast	Mod.			Fast	Mod.			Fast	Mod.
1276	UMHMNP87978312	−8.9	−9.0	1331	UMHMNP122970132	−8.1	−8.9	1386	UMHMNP112781256	−8.6	−8.9
1277	UMHMNP97614658	−10.5	−9.0	1332	UMHMNP15140597	−8.1	−8.9	1387	UMHMNP115982224	−8.6	−8.9
1278	UMHMNP102396156	−8.2	−9.0	1333	UMHMNP178180013	−8.1	−8.9	1388	UMHMNP141859996	−9.8	−8.9
1279	UMHMNP106849369	−8.9	−9.0	1334	UMHMNP186383073	−9.4	−8.9	1389	UMHMNP157878256	−8.0	−8.9
1280	UMHMNP107168592	−10.0	−9.0	1335	UMHMNP23839476	−8.1	−8.9	1390	UMHMNP17757072	−9.0	−8.9
1281	UMHMNP141860006	−8.8	−9.0	1336	UMHMNP517900526	−9.6	−8.9	1391	UMHMNP189514474	−8.3	−8.9
1282	UMHMNP145223650	−9.0	−9.0	1337	UMHMNP517900571	−8.3	−8.9	1392	UMHMNP26033103	−8.1	−8.9
1283	UMHMNP160324925	−8.7	−9.0	1338	UMHMNP80977	−8.4	−8.9	1393	UMHMNP31665302	−8.6	−8.9
1284	UMHMNP177196393	−8.8	−9.0	1339	UMHMNP81306618	−8.6	−8.9	1394	UMHMNP53586519	−8.8	−8.9
1285	UMHMNP22831647	−8.6	−9.0	1340	UMHMNP84871067	−8.2	−8.9	1395	UMHMNP69297514	−8.8	−8.9
1286	UMHMNP241166245	−8.2	−9.0	1341	UMHMNP88840011	−8.4	−8.9	1396	UMHMNP70206870	−8.9	−8.9
1287	UMHMNP265121060	−8.4	−9.0	1342	UMHMNP97484543	−8.7	−8.9	1397	UMHMNP78342399	−9.2	−8.9
1288	UMHMNP306997444	−8.2	−9.0	1343	UMHMNP102054513	−9.0	−8.9	1398	UMHMNP97042201	−8.5	−8.9
1289	UMHMNP516789	−8.9	−9.0	1344	UMHMNP116407182	−9.3	−8.9	1399	UMHMNP121994523	−8.8	−8.9
1290	UMHMNP55081419	−9.1	−9.0	1345	UMHMNP122535653	−9.2	−8.9	1400	UMHMNP122052953	−9.6	−8.9
1291	UMHMNP6120714	−8.2	−9.0	1346	UMHMNP125990207	−9.0	−8.9	1401	UMHMNP13163810	−8.3	−8.9
1292	UMHMNP65745473	−9.0	−9.0	1347	UMHMNP144398254	−8.3	−8.9	1402	UMHMNP133562513	−8.2	−8.9
1293	UMHMNP676132335	−8.5	−9.0	1348	UMHMNP157536351	−9.1	−8.9	1403	UMHMNP156310177	−8.3	−8.9
1294	UMHMNP777940735	−9.0	−9.0	1349	UMHMNP171528033	−9.4	−8.9	1404	UMHMNP161236631	−8.1	−8.9
1295	UMHMNP815586459	−9.6	−9.0	1350	UMHMNP179189027	−8.1	−8.9	1405	UMHMNP161776745	−8.1	−8.9
1296	UMHMNP85735144	−9.1	−9.0	1351	UMHMNP210969521	−8.6	−8.9	1406	UMHMNP165606688	−8.6	−8.9
1297	UMHMNP86126376	−9.1	−9.0	1352	UMHMNP287965291	−8.7	−8.9	1407	UMHMNP201800604	−8.6	−8.9
1298	UMHMNP111036409	−8.1	−9.0	1353	UMHMNP298196722	−8.1	−8.9	1408	UMHMNP385815305	−8.8	−8.9
1299	UMHMNP122771737	−8.5	−9.0	1354	UMHMNP498572900	−8.5	−8.9	1409	UMHMNP68654933	−10.0	−8.9
1300	UMHMNP22343460	−8.3	−9.0	1355	UMHMNP781646820	−8.7	−8.9	1410	UMHMNP72534124	−8.5	−8.9
1301	UMHMNP265121059	−8.6	−9.0	1356	UMHMNP791846827	−9.1	−8.9	1411	UMHMNP75179587	−10.1	−8.9
1302	UMHMNP2665045	−8.8	−9.0	1357	UMHMNP81256588	−8.8	−8.9	1412	UMHMNP852629619	−9.2	−8.9
1303	UMHMNP445471614	−9.2	−9.0	1358	UMHMNP99081795	−8.7	−8.9	1413	UMHMNP86851209	−8.8	−8.9
1304	UMHMNP681240551	−9.4	−9.0	1359	UMHMNP114995721	−9.0	−8.9	1414	UMHMNP110359994	−8.3	−8.9
1305	UMHMNP77674998	−8.4	−9.0	1360	UMHMNP138779892	−9.1	−8.9	1415	UMHMNP112781267	−8.9	−8.9
1306	UMHMNP117859173	−8.3	−8.9	1361	UMHMNP139083139	−8.8	−8.9	1416	UMHMNP132310919	−8.1	−8.9
1307	UMHMNP121940509	−8.9	−8.9	1362	UMHMNP155656001	−8.8	−8.9	1417	UMHMNP134029439	−8.1	−8.9
1308	UMHMNP123853690	−8.1	−8.9	1363	UMHMNP33646774	−8.4	−8.9	1418	UMHMNP139974757	−10.0	−8.9
1309	UMHMNP142060035	−9.1	−8.9	1364	UMHMNP338949090	−8.1	−8.9	1419	UMHMNP157924862	−8.3	−8.9
1310	UMHMNP147170090	−8.9	−8.9	1365	UMHMNP41398725	−8.6	−8.9	1420	UMHMNP211311089	−8.4	−8.9
1311	UMHMNP147641745	−8.9	−8.9	1366	UMHMNP545471	−8.5	−8.9	1421	UMHMNP326794165	−9.0	−8.9
1312	UMHMNP151890801	−8.8	−8.9	1367	UMHMNP61737960	−8.9	−8.9	1422	UMHMNP473739398	−9.5	−8.9
1313	UMHMNP425368645	−8.9	−8.9	1368	UMHMNP65754678	−9.1	−8.9	1423	UMHMNP53527289	−8.8	−8.9
1314	UMHMNP681227532	−8.5	−8.9	1369	UMHMNP77517330	−8.1	−8.9	1424	UMHMNP799808289	−8.2	−8.9
1315	UMHMNP682808908	−8.3	−8.9	1370	UMHMNP782491796	−8.8	−8.9	1425	UMHMNP804531462	−8.7	−8.9
1316	UMHMNP70329269	−9.8	−8.9	1371	UMHMNP849215958	−8.4	−8.9	1426	UMHMNP97530739	−9.1	−8.9
1317	UMHMNP72560373	−8.8	−8.9	1372	UMHMNP863493032	−8.6	−8.9	1427	UMHMNP113269424	−8.2	−8.9
1318	UMHMNP74145703	−8.2	−8.9	1373	UMHMNP95513636	−8.3	−8.9	1428	UMHMNP139975556	−8.3	−8.9
1319	UMHMNP862286713	−8.5	−8.9	1374	UMHMNP99250176	−8.3	−8.9	1429	UMHMNP142780276	−8.7	−8.9
1320	UMHMNP93474147	−8.8	−8.9	1375	UMHMNP149636920	−8.4	−8.9	1430	UMHMNP145075021	−8.3	−8.9
1321	UMHMNP144335159	−9.4	−8.9	1376	UMHMNP155656012	−8.7	−8.9	1431	UMHMNP151345083	−8.2	−8.9
1322	UMHMNP209408753	−9.8	−8.9	1377	UMHMNP23235678	−8.9	−8.9	1432	UMHMNP160316621	−9.1	−8.9
1323	UMHMNP20981593	−8.6	−8.9	1378	UMHMNP2572523	−9.0	−8.9	1433	UMHMNP161470220	−8.3	−8.9
1324	UMHMNP228575244	−8.6	−8.9	1379	UMHMNP346706476	−8.6	−8.9	1434	UMHMNP169387751	−8.4	−8.9
1325	UMHMNP338949103	−8.5	−8.9	1380	UMHMNP53190888	−9.0	−8.9	1435	UMHMNP173693486	−8.6	−8.9
1326	UMHMNP60422880	−8.6	−8.9	1381	UMHMNP681240540	−8.5	−8.9	1436	UMHMNP199165883	−9.9	−8.9
1327	UMHMNP62861136	−8.6	−8.9	1382	UMHMNP70117214	−8.6	−8.9	1437	UMHMNP433264621	−9.6	−8.9
1328	UMHMNP781646795	−8.4	−8.9	1383	UMHMNP74175943	−9.8	−8.9	1438	UMHMNP6277141	−9.0	−8.9
1329	UMHMNP85733706	−8.3	−8.9	1384	UMHMNP102396214	−10.7	−8.9	1439	UMHMNP64190701	−8.6	−8.9
1330	UMHMNP111768162	−9.1	−8.9	1385	UMHMNP104387146	−8.7	−8.9	1440	UMHMNP78518737	−8.2	−8.9

Table S1. *Continued.*

No.	Compound Name/Code	Docking Score (kcal/mol)		No.	Compound Name/Code	Docking Score (kcal/mol)		No.	Compound Name/Code	Docking Score (kcal/mol)	
		Fast	Mod.			Fast	Mod.			Fast	Mod.
1441	UMHMNP83136883	−8.4	−8.9	1496	UMHMNP5259289	−9.2	−8.8	1551	UMHMNP901564	−9.0	−8.8
1442	UMHMNP88640215	−8.5	−8.9	1497	UMHMNP566267	−8.5	−8.8	1552	UMHMNP92804687	−9.2	−8.8
1443	UMHMNP90375603	−8.3	−8.9	1498	UMHMNP62073794	−9.3	−8.8	1553	UMHMNP113831008	−9.2	−8.8
1444	UMHMNP1042597	−8.9	−8.8	1499	UMHMNP73622216	−8.2	−8.8	1554	UMHMNP11598231	−8.9	−8.8
1445	UMHMNP120963613	−8.0	−8.8	1500	UMHMNP75605866	−8.2	−8.8	1555	UMHMNP150417699	−8.2	−8.8
1446	UMHMNP132278412	−8.4	−8.8	1501	UMHMNP130203677	−10.7	−8.8	1556	UMHMNP190451048	−8.8	−8.8
1447	UMHMNP136762975	−8.4	−8.8	1502	UMHMNP138843206	−9.1	−8.8	1557	UMHMNP23820760	−8.8	−8.8
1448	UMHMNP146471843	−9.5	−8.8	1503	UMHMNP151345107	−8.8	−8.8	1558	UMHMNP27857121	−9.7	−8.8
1449	UMHMNP149298000	−8.5	−8.8	1504	UMHMNP176181865	−8.4	−8.8	1559	UMHMNP306997411	−8.7	−8.8
1450	UMHMNP159518771	−8.6	−8.8	1505	UMHMNP2034744	−8.9	−8.8	1560	UMHMNP5241247	−9.6	−8.8
1451	UMHMNP16649415	−8.9	−8.8	1506	UMHMNP23983439	−9.0	−8.8	1561	UMHMNP561637	−10.9	−8.8
1452	UMHMNP221367904	−8.8	−8.8	1507	UMHMNP267407250	−8.7	−8.8	1562	UMHMNP732296536	−8.3	−8.8
1453	UMHMNP256377681	−9.8	−8.8	1508	UMHMNP50391725	−8.5	−8.8	1563	UMHMNP737006621	−8.8	−8.8
1454	UMHMNP273746397	−9.6	−8.8	1509	UMHMNP59957403	−8.2	−8.8	1564	UMHMNP753001739	−8.9	−8.8
1455	UMHMNP4575740	−8.4	−8.8	1510	UMHMNP65754634	−8.4	−8.8	1565	UMHMNP79849379	−8.3	−8.8
1456	UMHMNP459412056	−8.4	−8.8	1511	UMHMNP73436358	−8.2	−8.8	1566	UMHMNP109872459	−9.9	−8.8
1457	UMHMNP479637064	−8.9	−8.8	1512	UMHMNP750594	−9.6	−8.8	1567	UMHMNP145525333	−9.4	−8.8
1458	UMHMNP51489_p0	−8.4	−8.8	1513	UMHMNP113626769	−8.4	−8.8	1568	UMHMNP146488633	−8.4	−8.8
1459	UMHMNP62073863	−8.7	−8.8	1514	UMHMNP121442248	−8.3	−8.8	1569	UMHMNP154205193	−8.5	−8.8
1460	UMHMNP68671487	−8.6	−8.8	1515	UMHMNP123794309	−9.0	−8.8	1570	UMHMNP168781791	−8.1	−8.8
1461	UMHMNP69081874	−8.8	−8.8	1516	UMHMNP142677109	−10.3	−8.8	1571	UMHMNP171440236	−8.6	−8.8
1462	UMHMNP70214981	−9.2	−8.8	1517	UMHMNP145398625	−8.2	−8.8	1572	UMHMNP29478419	−8.2	−8.8
1463	UMHMNP81657785	−8.5	−8.8	1518	UMHMNP194020412	−9.5	−8.8	1573	UMHMNP36564291	−9.5	−8.8
1464	UMHMNP84323295	−8.3	−8.8	1519	UMHMNP205750250	−8.3	−8.8	1574	UMHMNP4192147	−9.2	−8.8
1465	UMHMNP85733693	−8.7	−8.8	1520	UMHMNP211311103	−9.2	−8.8	1575	UMHMNP433717556	−8.1	−8.8
1466	UMHMNP90195407	−9.9	−8.8	1521	UMHMNP211555032	−8.5	−8.8	1576	UMHMNP474779754	−9.7	−8.8
1467	UMHMNP105969651	−8.7	−8.8	1522	UMHMNP259670843	−8.1	−8.8	1577	UMHMNP60422879	−8.1	−8.8
1468	UMHMNP112514428	−8.2	−8.8	1523	UMHMNP263764029	−9.8	−8.8	1578	UMHMNP7069423	−8.4	−8.8
1469	UMHMNP116102400	−8.4	−8.8	1524	UMHMNP351441184	−9.6	−8.8	1579	UMHMNP97877708	−8.4	−8.8
1470	UMHMNP1236142	−9.3	−8.8	1525	UMHMNP474680	−8.9	−8.8	1580	UMHMNP102054502	−8.8	−8.8
1471	UMHMNP155521203	−8.4	−8.8	1526	UMHMNP54848292	−9.1	−8.8	1581	UMHMNP108675645	−8.2	−8.8
1472	UMHMNP175861786	−9.5	−8.8	1527	UMHMNP55088750	−9.1	−8.8	1582	UMHMNP11052327	−8.3	−8.8
1473	UMHMNP329050217	−9.0	−8.8	1528	UMHMNP63814493	−8.8	−8.8	1583	UMHMNP115982213	−8.3	−8.8
1474	UMHMNP65754623	−8.9	−8.8	1529	UMHMNP663597851	−8.1	−8.8	1584	UMHMNP122535642	−8.8	−8.8
1475	UMHMNP74799589	−9.6	−8.8	1530	UMHMNP708255985	−8.6	−8.8	1585	UMHMNP133625260	−9.4	−8.8
1476	UMHMNP78370851	−9.0	−8.8	1531	UMHMNP794536928	−8.0	−8.8	1586	UMHMNP134981809	−9.5	−8.8
1477	UMHMNP83704147	−8.2	−8.8	1532	UMHMNP80388472	−8.1	−8.8	1587	UMHMNP139083219	−9.7	−8.8
1478	UMHMNP856414567	−9.2	−8.8	1533	UMHMNP85650255	−9.2	−8.8	1588	UMHMNP191212370	−9.4	−8.8
1479	UMHMNP856414603	−8.3	−8.8	1534	UMHMNP862286735	−9.0	−8.8	1589	UMHMNP191803404	−8.3	−8.8
1480	UMHMNP85733728	−9.3	−8.8	1535	UMHMNP87532261	−9.7	−8.8	1590	UMHMNP479201564	−8.1	−8.8
1481	UMHMNP95043188	−8.1	−8.8	1536	UMHMNP94935996	−8.4	−8.8	1591	UMHMNP547988	−8.7	−8.8
1482	UMHMNP984872	−8.1	−8.8	1537	UMHMNP99481535	−8.4	−8.8	1592	UMHMNP55708742	−10.1	−8.8
1483	UMHMNP99081784	−8.1	−8.8	1538	UMHMNP133587163	−8.2	−8.8	1593	UMHMNP56362426	−8.2	−8.8
1484	UMHMNP107748878	−8.7	−8.8	1539	UMHMNP144587564	−8.8	−8.8	1594	UMHMNP63250226	−8.9	−8.8
1485	UMHMNP122052942	−8.5	−8.8	1540	UMHMNP175861866	−9.5	−8.8	1595	UMHMNP681240642	−9.3	−8.8
1486	UMHMNP122353291	−9.0	−8.8	1541	UMHMNP179733154	−8.2	−8.8	1596	UMHMNP92125694	−8.6	−8.8
1487	UMHMNP1256866	−9.8	−8.8	1542	UMHMNP186820553	−8.8	−8.8	1597	UMHMNP116428638	−8.3	−8.8
1488	UMHMNP132410403	−8.1	−8.8	1543	UMHMNP189083792	−8.2	−8.8	1598	UMHMNP118169361	−9.5	−8.8
1489	UMHMNP132774211	−9.0	−8.8	1544	UMHMNP201213027	−8.8	−8.8	1599	UMHMNP156953876	−8.6	−8.8
1490	UMHMNP151029413	−10.7	−8.8	1545	UMHMNP213749816	−8.6	−8.8	1600	UMHMNP1715862	−8.9	−8.8
1491	UMHMNP152213677	−8.7	−8.8	1546	UMHMNP2790321	−8.6	−8.8	1601	UMHMNP182806140	−8.7	−8.8
1492	UMHMNP184348398	−9.1	−8.8	1547	UMHMNP65754690	−8.3	−8.8	1602	UMHMNP185331968	−8.9	−8.8
1493	UMHMNP193619420	−8.7	−8.8	1548	UMHMNP81575782	−9.7	−8.8	1603	UMHMNP28636251	−9.8	−8.8
1494	UMHMNP19633955	−8.3	−8.8	1549	UMHMNP84323284	−9.9	−8.8	1604	UMHMNP387791	−9.2	−8.8
1495	UMHMNP196868005	−8.4	−8.8	1550	UMHMNP87411845	−9.1	−8.8	1605	UMHMNP571176948	−8.6	−8.8

Table S1. *Continued.*

No.	Compound Name/Code	Docking Score (kcal/mol)		No.	Compound Name/Code	Docking Score (kcal/mol)		No.	Compound Name/Code	Docking Score (kcal/mol)	
		Fast	Mod.			Fast	Mod.			Fast	Mod.
1606	UMHMNP61263830	−8.1	−8.8	1661	UMHMNP93379578	−9.3	−8.7	1716	UMHMNP40517860	−8.4	−8.7
1607	UMHMNP81387873	−9.2	−8.8	1662	UMHMNP100046046	−9.1	−8.7	1717	UMHMNP445471590	−8.3	−8.7
1608	UMHMNP83995044	−8.9	−8.8	1663	UMHMNP104855201	−8.4	−8.7	1718	UMHMNP472877	−8.8	−8.7
1609	UMHMNP856414523	−8.9	−8.8	1664	UMHMNP110271222	−8.8	−8.7	1719	UMHMNP53866367	−9.7	−8.7
1610	UMHMNP90195430	−10.0	−8.8	1665	UMHMNP126723168	−8.6	−8.7	1720	UMHMNP55081420	−9.1	−8.7
1611	UMHMNP90735436	−8.6	−8.8	1666	UMHMNP135048598	−8.7	−8.7	1721	UMHMNP63693185	−9.0	−8.7
1612	UMHMNP10395365	−8.2	−8.7	1667	UMHMNP157469404	−8.1	−8.7	1722	UMHMNP67506198	−9.6	−8.7
1613	UMHMNP105064297	−9.5	−8.7	1668	UMHMNP157622612	−8.1	−8.7	1723	UMHMNP71239651	−8.5	−8.7
1614	UMHMNP122271914	−9.3	−8.7	1669	UMHMNP20224708	−9.2	−8.7	1724	UMHMNP74055439	−8.5	−8.7
1615	UMHMNP122876691	−8.9	−8.7	1670	UMHMNP204716063	−8.6	−8.7	1725	UMHMNP95513647	−10.3	−8.7
1616	UMHMNP1232195	−8.4	−8.7	1671	UMHMNP212069506	−8.4	−8.7	1726	UMHMNP127524598	−8.1	−8.7
1617	UMHMNP128631209	−8.2	−8.7	1672	UMHMNP24041687	−8.4	−8.7	1727	UMHMNP139765381	−9.4	−8.7
1618	UMHMNP131501274	−8.9	−8.7	1673	UMHMNP474417544	−8.2	−8.7	1728	UMHMNP144686419	−9.1	−8.7
1619	UMHMNP1406162	−8.5	−8.7	1674	UMHMNP55303996	−9.2	−8.7	1729	UMHMNP147641734	−9.5	−8.7
1620	UMHMNP186313008	−8.3	−8.7	1675	UMHMNP62858257	−9.2	−8.7	1730	UMHMNP157047580	−10.4	−8.7
1621	UMHMNP34218945	−8.9	−8.7	1676	UMHMNP85650244	−9.2	−8.7	1731	UMHMNP204716030	−8.9	−8.7
1622	UMHMNP446862700	−8.6	−8.7	1677	UMHMNP90038247	−8.5	−8.7	1732	UMHMNP272458329	−9.6	−8.7
1623	UMHMNP52043002	−9.2	−8.7	1678	UMHMNP105815905	−9.9	−8.7	1733	UMHMNP286437749	−8.3	−8.7
1624	UMHMNP648883301	−10.3	−8.7	1679	UMHMNP106521613	−8.9	−8.7	1734	UMHMNP55870014	−8.4	−8.7
1625	UMHMNP79067735	−9.4	−8.7	1680	UMHMNP129369422	−8.1	−8.7	1735	UMHMNP64118747	−9.5	−8.7
1626	UMHMNP862587175	−8.4	−8.7	1681	UMHMNP141544594	−9.0	−8.7	1736	UMHMNP66082313	−8.1	−8.7
1627	UMHMNP100244368	−8.7	−8.7	1682	UMHMNP15073844	−9.3	−8.7	1737	UMHMNP79801	−9.2	−8.7
1628	UMHMNP105708756	−9.1	−8.7	1683	UMHMNP208708241	−8.7	−8.7	1738	UMHMNP85337139	−10.1	−8.7
1629	UMHMNP126622637	−8.0	−8.7	1684	UMHMNP288851310	−9.2	−8.7	1739	UMHMNP86047145	−8.5	−8.7
1630	UMHMNP134779343	−8.2	−8.7	1685	UMHMNP434162	−8.1	−8.7	1740	UMHMNP86766010	−8.6	−8.7
1631	UMHMNP135626561	−9.7	−8.7	1686	UMHMNP55831271	−9.4	−8.7	1741	UMHMNP871117805	−8.2	−8.7
1632	UMHMNP151171185	−9.2	−8.7	1687	UMHMNP78285968	−8.5	−8.7	1742	UMHMNP112058045	−8.6	−8.7
1633	UMHMNP155416345	−8.6	−8.7	1688	UMHMNP81387828	−8.6	−8.7	1743	UMHMNP117823344	−9.0	−8.7
1634	UMHMNP168434137	−8.7	−8.7	1689	UMHMNP889890613	−9.5	−8.7	1744	UMHMNP126026571	−8.7	−8.7
1635	UMHMNP172723352	−10.9	−8.7	1690	UMHMNP95062309	−8.1	−8.7	1745	UMHMNP132410389	−8.8	−8.7
1636	UMHMNP185543924	−10.0	−8.7	1691	UMHMNP95513614	−9.1	−8.7	1746	UMHMNP133738439	−8.8	−8.7
1637	UMHMNP197381638	−9.7	−8.7	1692	UMHMNP99617455	−9.0	−8.7	1747	UMHMNP136763003	−8.5	−8.7
1638	UMHMNP526432	−8.9	−8.7	1693	UMHMNP133401135	−9.2	−8.7	1748	UMHMNP156312071	−8.1	−8.7
1639	UMHMNP54602196	−8.9	−8.7	1694	UMHMNP135529690	−9.4	−8.7	1749	UMHMNP156953843	−9.8	−8.7
1640	UMHMNP56258323	−9.5	−8.7	1695	UMHMNP135574617	−8.8	−8.7	1750	UMHMNP163136047	−8.5	−8.7
1641	UMHMNP63015918	−8.2	−8.7	1696	UMHMNP160522156	−9.7	−8.7	1751	UMHMNP164081032	−8.2	−8.7
1642	UMHMNP76907636	−8.6	−8.7	1697	UMHMNP169340005	−8.6	−8.7	1752	UMHMNP185139315	−8.1	−8.7
1643	UMHMNP83995055	−9.7	−8.7	1698	UMHMNP516881	−8.2	−8.7	1753	UMHMNP220503297	−8.9	−8.7
1644	UMHMNP97614636	−8.1	−8.7	1699	UMHMNP566654	−9.7	−8.7	1754	UMHMNP273202850	−8.4	−8.7
1645	UMHMNP99957263	−8.3	−8.7	1700	UMHMNP79781661	−8.7	−8.7	1755	UMHMNP356566820	−8.6	−8.7
1646	UMHMNP106001381	−8.3	−8.7	1701	UMHMNP847450753	−8.2	−8.7	1756	UMHMNP37717058	−9.2	−8.7
1647	UMHMNP115141474	−8.3	−8.7	1702	UMHMNP90267917	−8.7	−8.7	1757	UMHMNP405274171	−9.2	−8.7
1648	UMHMNP130799370	−9.7	−8.7	1703	UMHMNP99081762	−8.8	−8.7	1758	UMHMNP71841979	−8.2	−8.7
1649	UMHMNP145398716	−8.4	−8.7	1704	UMHMNP100019247	−8.6	−8.7	1759	UMHMNP76758195	−9.0	−8.7
1650	UMHMNP17297788	−10.3	−8.7	1705	UMHMNP112523883	−8.2	−8.7	1760	UMHMNP79664623	−8.7	−8.7
1651	UMHMNP212069493	−9.0	−8.7	1706	UMHMNP114915343	−9.8	−8.7	1761	UMHMNP110612287	−9.0	−8.7
1652	UMHMNP28056191	−8.9	−8.7	1707	UMHMNP126572734	−8.1	−8.7	1762	UMHMNP111427975	−8.3	−8.7
1653	UMHMNP435327063	−8.6	−8.7	1708	UMHMNP133883100	−8.1	−8.7	1763	UMHMNP118964366	−8.1	−8.7
1654	UMHMNP446862711	−8.4	−8.7	1709	UMHMNP134887299	−8.4	−8.7	1764	UMHMNP143007298	−8.2	−8.7
1655	UMHMNP449166001	−10.2	−8.7	1710	UMHMNP156953956	−8.9	−8.7	1765	UMHMNP156352646	−9.4	−8.7
1656	UMHMNP5241225	−8.2	−8.7	1711	UMHMNP158761138	−9.3	−8.7	1766	UMHMNP161099543	−8.9	−8.7
1657	UMHMNP678183932	−8.6	−8.7	1712	UMHMNP169217463	−8.8	−8.7	1767	UMHMNP164672553	−10.1	−8.7
1658	UMHMNP690627555	−8.2	−8.7	1713	UMHMNP181034980	−11.1	−8.7	1768	UMHMNP169387762	−8.2	−8.7
1659	UMHMNP690627566	−8.3	−8.7	1714	UMHMNP34350840	−9.5	−8.7	1769	UMHMNP20780374	−8.4	−8.7
1660	UMHMNP90195394	−9.2	−8.7	1715	UMHMNP365541680	−8.2	−8.7	1770	UMHMNP2130178	−8.4	−8.7

Table S1. *Continued.*

No.	Compound Name/Code	Docking Score (kcal/mol)		No.	Compound Name/Code	Docking Score (kcal/mol)		No.	Compound Name/Code	Docking Score (kcal/mol)	
		Fast	Mod.			Fast	Mod.			Fast	Mod.
1771	UMHMNP255882098	−9.2	−8.7	1826	UMHMNP171090816	−9.7	−8.6	1881	UMHMNP200437667	−8.3	−8.6
1772	UMHMNP4651483	−8.1	−8.7	1827	UMHMNP174024976	−8.9	−8.6	1882	UMHMNP233607653	−9.0	−8.6
1773	UMHMNP509095209	−8.3	−8.7	1828	UMHMNP188558012	−8.9	−8.6	1883	UMHMNP452934962	−9.2	−8.6
1774	UMHMNP549521280	−8.7	−8.7	1829	UMHMNP188558501	−8.1	−8.6	1884	UMHMNP675106215	−9.1	−8.6
1775	UMHMNP58844614	−9.3	−8.7	1830	UMHMNP207385088	−8.2	−8.6	1885	UMHMNP676348940	−9.2	−8.6
1776	UMHMNP70363870	−8.1	−8.7	1831	UMHMNP22488626	−9.7	−8.6	1886	UMHMNP79405688	−11.0	−8.6
1777	UMHMNP856414501	−9.5	−8.7	1832	UMHMNP343573615	−9.3	−8.6	1887	UMHMNP868395908	−9.4	−8.6
1778	UMHMNP85733751	−8.5	−8.7	1833	UMHMNP351198099	−8.3	−8.6	1888	UMHMNP872721841	−9.3	−8.6
1779	UMHMNP95513625	−8.2	−8.7	1834	UMHMNP465952	−8.5	−8.6	1889	UMHMNP96999358	−8.2	−8.6
1780	UMHMNP10583047	−8.3	−8.6	1835	UMHMNP53190899	−8.6	−8.6	1890	UMHMNP114915321	−8.7	−8.6
1781	UMHMNP106211910	−8.1	−8.6	1836	UMHMNP55486061	−8.6	−8.6	1891	UMHMNP138629897	−8.5	−8.6
1782	UMHMNP117694970	−8.3	−8.6	1837	UMHMNP57291880	−8.6	−8.6	1892	UMHMNP141321122	−9.1	−8.6
1783	UMHMNP131998544	−9.6	−8.6	1838	UMHMNP578745_t0	−8.4	−8.6	1893	UMHMNP158761127	−9.0	−8.6
1784	UMHMNP132410367	−8.3	−8.6	1839	UMHMNP623167704	−9.8	−8.6	1894	UMHMNP164991655	−10.7	−8.6
1785	UMHMNP133607860	−9.2	−8.6	1840	UMHMNP82471156	−8.7	−8.6	1895	UMHMNP176704077	−9.1	−8.6
1786	UMHMNP145940745	−8.7	−8.6	1841	UMHMNP858950364	−9.1	−8.6	1896	UMHMNP181423712	−8.3	−8.6
1787	UMHMNP148000151	−9.2	−8.6	1842	UMHMNP95513578	−8.9	−8.6	1897	UMHMNP197381694	−8.6	−8.6
1788	UMHMNP159898110	−8.1	−8.6	1843	UMHMNP99624109	−8.7	−8.6	1898	UMHMNP39832683	−8.9	−8.6
1789	UMHMNP165815838	−8.4	−8.6	1844	UMHMNP100667741	−8.6	−8.6	1899	UMHMNP466690551	−8.7	−8.6
1790	UMHMNP170473628	−8.5	−8.6	1845	UMHMNP13327792	−8.2	−8.6	1900	UMHMNP55897773	−8.1	−8.6
1791	UMHMNP221169773	−8.3	−8.6	1846	UMHMNP142060002	−8.1	−8.6	1901	UMHMNP64825805	−8.2	−8.6
1792	UMHMNP29953502	−8.1	−8.6	1847	UMHMNP165337804	−9.7	−8.6	1902	UMHMNP866403750	−8.6	−8.6
1793	UMHMNP310396691	−8.5	−8.6	1848	UMHMNP165967000	−8.4	−8.6	1903	UMHMNP92679077	−8.1	−8.6
1794	UMHMNP5258866	−8.9	−8.6	1849	UMHMNP169565780	−8.4	−8.6	1904	UMHMNP97190404	−9.2	−8.6
1795	UMHMNP781646784	−9.0	−8.6	1850	UMHMNP478945652	−8.7	−8.6	1905	UMHMNP114774866	−8.5	−8.6
1796	UMHMNP871117792	−8.1	−8.6	1851	UMHMNP57103205	−9.1	−8.6	1906	UMHMNP123200566	−8.5	−8.6
1797	UMHMNP108695825	−9.3	−8.6	1852	UMHMNP70206869	−8.2	−8.6	1907	UMHMNP129602182	−8.8	−8.6
1798	UMHMNP119105178	−8.1	−8.6	1853	UMHMNP80999	−8.8	−8.6	1908	UMHMNP145398705	−8.7	−8.6
1799	UMHMNP142677096	−10.2	−8.6	1854	UMHMNP81855461	−8.6	−8.6	1909	UMHMNP156848678	−8.1	−8.6
1800	UMHMNP169565871	−9.5	−8.6	1855	UMHMNP82054215	−9.4	−8.6	1910	UMHMNP211118988	−9.9	−8.6
1801	UMHMNP250727016	−8.3	−8.6	1856	UMHMNP876754777	−8.2	−8.6	1911	UMHMNP221012799	−9.4	−8.6
1802	UMHMNP263744736	−9.9	−8.6	1857	UMHMNP133805035	−8.0	−8.6	1912	UMHMNP30674329	−8.2	−8.6
1803	UMHMNP475111796	−8.5	−8.6	1858	UMHMNP154512239	−9.3	−8.6	1913	UMHMNP329050193	−8.3	−8.6
1804	UMHMNP815577436	−8.8	−8.6	1859	UMHMNP168004862	−8.2	−8.6	1914	UMHMNP395070890	−8.3	−8.6
1805	UMHMNP847450731	−8.4	−8.6	1860	UMHMNP171370562	−8.2	−8.6	1915	UMHMNP433717307	−8.6	−8.6
1806	UMHMNP865668571	−9.1	−8.6	1861	UMHMNP196868016	−9.1	−8.6	1916	UMHMNP51847837	−9.3	−8.6
1807	UMHMNP121819703	−8.6	−8.6	1862	UMHMNP220866397	−9.3	−8.6	1917	UMHMNP64907262	−8.1	−8.6
1808	UMHMNP135446843	−9.6	−8.6	1863	UMHMNP247154638	−9.0	−8.6	1918	UMHMNP94773189	−8.4	−8.6
1809	UMHMNP159001975	−8.5	−8.6	1864	UMHMNP74323447	−9.5	−8.6	1919	UMHMNP10245815	−10.1	−8.5
1810	UMHMNP1778025	−8.3	−8.6	1865	UMHMNP74847099	−8.4	−8.6	1920	UMHMNP108695803	−8.8	−8.5
1811	UMHMNP190451059	−8.1	−8.6	1866	UMHMNP813436407	−9.4	−8.6	1921	UMHMNP111621375	−8.8	−8.5
1812	UMHMNP213547263	−8.5	−8.6	1867	UMHMNP83542169	−8.1	−8.6	1922	UMHMNP126060091	−8.2	−8.5
1813	UMHMNP217478734	−9.4	−8.6	1868	UMHMNP90996240	−8.3	−8.6	1923	UMHMNP130799358	−10.9	−8.5
1814	UMHMNP220304552	−8.3	−8.6	1869	UMHMNP114742722	−9.4	−8.6	1924	UMHMNP189748832	−9.4	−8.5
1815	UMHMNP257904942	−9.4	−8.6	1870	UMHMNP13327770	−8.8	−8.6	1925	UMHMNP215854061	−8.8	−8.5
1816	UMHMNP257904953	−8.4	−8.6	1871	UMHMNP221160801	−10.8	−8.6	1926	UMHMNP272118055	−9.6	−8.5
1817	UMHMNP329050240	−8.3	−8.6	1872	UMHMNP661476659	−8.1	−8.6	1927	UMHMNP481196	−8.4	−8.5
1818	UMHMNP439132044	−8.6	−8.6	1873	UMHMNP80387902	−8.4	−8.6	1928	UMHMNP50657313	−9.0	−8.5
1819	UMHMNP67067816	−8.8	−8.6	1874	UMHMNP97190426	−9.0	−8.6	1929	UMHMNP63250215	−8.4	−8.5
1820	UMHMNP81306594	−11.4	−8.6	1875	UMHMNP114250_t0	−8.1	−8.6	1930	UMHMNP74608632	−9.7	−8.5
1821	UMHMNP113830958	−9.7	−8.6	1876	UMHMNP116407262	−8.2	−8.6	1931	UMHMNP79206716	−8.3	−8.5
1822	UMHMNP114582728	−9.4	−8.6	1877	UMHMNP121350990	−8.7	−8.6	1932	UMHMNP848132387	−8.2	−8.5
1823	UMHMNP120314150	−8.4	−8.6	1878	UMHMNP174792020	−9.2	−8.6	1933	UMHMNP89837990	−9.3	−8.5
1824	UMHMNP133943507	−8.7	−8.6	1879	UMHMNP183006793	−8.7	−8.6	1934	UMHMNP90996239	−8.2	−8.5
1825	UMHMNP168113675	−8.7	−8.6	1880	UMHMNP200273661	−9.4	−8.6	1935	UMHMNP95062376	−8.6	−8.5

Table S1. *Continued.*

No.	Compound Name/Code	Docking Score (kcal/mol)		No.	Compound Name/Code	Docking Score (kcal/mol)		No.	Compound Name/Code	Docking Score (kcal/mol)	
		Fast	Mod.			Fast	Mod.			Fast	Mod.
1936	UMHMNP104855223	−8.3	−8.5	1991	UMHMNP118984477	−8.4	−8.5	2046	UMHMNP104993104	−8.2	−8.5
1937	UMHMNP106009807	−8.0	−8.5	1992	UMHMNP128049149	−8.0	−8.5	2047	UMHMNP110271211	−8.1	−8.5
1938	UMHMNP140866199	−8.2	−8.5	1993	UMHMNP149864700	−8.7	−8.5	2048	UMHMNP143380409	−9.0	−8.5
1939	UMHMNP162830242	−8.5	−8.5	1994	UMHMNP154071702	−8.9	−8.5	2049	UMHMNP151232836	−8.7	−8.5
1940	UMHMNP21321959	−8.1	−8.5	1995	UMHMNP194856423	−8.6	−8.5	2050	UMHMNP157232592	−8.6	−8.5
1941	UMHMNP63216217	−9.4	−8.5	1996	UMHMNP21072639	−9.0	−8.5	2051	UMHMNP174232350	−8.8	−8.5
1942	UMHMNP693790817	−8.9	−8.5	1997	UMHMNP65166021	−8.7	−8.5	2052	UMHMNP182806093	−9.8	−8.5
1943	UMHMNP74145736	−8.9	−8.5	1998	UMHMNP66879865	−8.7	−8.5	2053	UMHMNP18964539	−8.9	−8.5
1944	UMHMNP76520520	−10.4	−8.5	1999	UMHMNP78285957	−8.1	−8.5	2054	UMHMNP676271844	−10.1	−8.5
1945	UMHMNP80246248	−9.9	−8.5	2000	UMHMNP98112415	−8.2	−8.5	2055	UMHMNP6853992	−8.9	−8.5
1946	UMHMNP80860533	−8.4	−8.5	2001	UMHMNP104311708	−8.1	−8.5	2056	UMHMNP73538564	−9.4	−8.5
1947	UMHMNP849904887	−8.7	−8.5	2002	UMHMNP120152003	−8.8	−8.5	2057	UMHMNP89210139	−8.7	−8.5
1948	UMHMNP104992941	−8.3	−8.5	2003	UMHMNP165815792	−8.1	−8.5	2058	UMHMNP97190437	−10.4	−8.5
1949	UMHMNP128364307	−9.9	−8.5	2004	UMHMNP168422397	−9.6	−8.5	2059	UMHMNP116314	−8.2	−8.4
1950	UMHMNP138355074	−8.1	−8.5	2005	UMHMNP215035828	−8.6	−8.5	2060	UMHMNP116339969	−10.3	−8.4
1951	UMHMNP141394710	−8.2	−8.5	2006	UMHMNP221012788	−9.2	−8.5	2061	UMHMNP116407193	−9.3	−8.4
1952	UMHMNP149633567	−9.9	−8.5	2007	UMHMNP359415660	−8.5	−8.5	2062	UMHMNP123314227	−8.9	−8.4
1953	UMHMNP161187786	−10.1	−8.5	2008	UMHMNP39707556	−8.7	−8.5	2063	UMHMNP139083220	−8.8	−8.4
1954	UMHMNP211239495	−8.7	−8.5	2009	UMHMNP471278736	−8.2	−8.5	2064	UMHMNP146471854	−8.4	−8.4
1955	UMHMNP222960896	−8.9	−8.5	2010	UMHMNP475111809	−8.9	−8.5	2065	UMHMNP149155215	−9.0	−8.4
1956	UMHMNP4351557	−9.1	−8.5	2011	UMHMNP625835236	−9.2	−8.5	2066	UMHMNP159934153	−8.5	−8.4
1957	UMHMNP749216475	−8.6	−8.5	2012	UMHMNP109802177	−9.3	−8.5	2067	UMHMNP180603649	−8.7	−8.4
1958	UMHMNP781646875	−8.5	−8.5	2013	UMHMNP123492400	−8.1	−8.5	2068	UMHMNP200273672	−8.3	−8.4
1959	UMHMNP862588816	−8.6	−8.5	2014	UMHMNP136196651	−8.6	−8.5	2069	UMHMNP612089857	−9.6	−8.4
1960	UMHMNP99457984	−8.4	−8.5	2015	UMHMNP139953336	−10.3	−8.5	2070	UMHMNP63814539	−8.9	−8.4
1961	UMHMNP135048587	−8.1	−8.5	2016	UMHMNP141394721	−8.1	−8.5	2071	UMHMNP706784749	−8.3	−8.4
1962	UMHMNP139765289	−8.8	−8.5	2017	UMHMNP155112688	−8.1	−8.5	2072	UMHMNP70941332	−8.3	−8.4
1963	UMHMNP143592250	−8.5	−8.5	2018	UMHMNP199600389	−9.0	−8.5	2073	UMHMNP733753156	−8.1	−8.4
1964	UMHMNP145398614	−9.0	−8.5	2019	UMHMNP23637312	−10.0	−8.5	2074	UMHMNP75266238	−8.2	−8.4
1965	UMHMNP149378585	−11.3	−8.5	2020	UMHMNP366479041	−8.7	−8.5	2075	UMHMNP77209257	−8.4	−8.4
1966	UMHMNP15361407	−8.4	−8.5	2021	UMHMNP53823044	−8.1	−8.5	2076	UMHMNP863116487	−9.2	−8.4
1967	UMHMNP219517483	−9.3	−8.5	2022	UMHMNP60422891	−8.7	−8.5	2077	UMHMNP133883133	−8.1	−8.4
1968	UMHMNP24587415	−8.6	−8.5	2023	UMHMNP648883323	−8.6	−8.5	2078	UMHMNP138264503	−8.4	−8.4
1969	UMHMNP55623375	−8.9	−8.5	2024	UMHMNP681145471	−9.1	−8.5	2079	UMHMNP139765392	−9.7	−8.4
1970	UMHMNP693790806	−8.9	−8.5	2025	UMHMNP73436449	−9.5	−8.5	2080	UMHMNP147217218	−8.5	−8.4
1971	UMHMNP83704114	−8.3	−8.5	2026	UMHMNP111149872	−8.9	−8.5	2081	UMHMNP184430000	−8.4	−8.4
1972	UMHMNP91893833	−8.6	−8.5	2027	UMHMNP111863673	−8.6	−8.5	2082	UMHMNP192187670	−8.3	−8.4
1973	UMHMNP95062387	−9.6	−8.5	2028	UMHMNP120853136	−8.6	−8.5	2083	UMHMNP201800591	−8.7	−8.4
1974	UMHMNP97190346	−8.9	−8.5	2029	UMHMNP136762953	−8.4	−8.5	2084	UMHMNP225779771	−9.1	−8.4
1975	UMHMNP117472976	−9.0	−8.5	2030	UMHMNP139579274	−8.3	−8.5	2085	UMHMNP247115282	−8.3	−8.4
1976	UMHMNP117694992	−8.2	−8.5	2031	UMHMNP142780492	−10.1	−8.5	2086	UMHMNP288858560	−8.8	−8.4
1977	UMHMNP133530242	−9.8	−8.5	2032	UMHMNP142979348	−8.9	−8.5	2087	UMHMNP3633725	−9.9	−8.4
1978	UMHMNP144027781	−8.3	−8.5	2033	UMHMNP143380669	−10.1	−8.5	2088	UMHMNP445430659	−8.8	−8.4
1979	UMHMNP146488644	−9.1	−8.5	2034	UMHMNP143592227	−10.0	−8.5	2089	UMHMNP4657583	−8.4	−8.4
1980	UMHMNP146555820	−8.1	−8.5	2035	UMHMNP16852521	−8.2	−8.5	2090	UMHMNP676154293	−8.1	−8.4
1981	UMHMNP212069517	−8.5	−8.5	2036	UMHMNP205382236	−8.6	−8.5	2091	UMHMNP681145460	−8.7	−8.4
1982	UMHMNP306971108	−8.2	−8.5	2037	UMHMNP237749847	−8.2	−8.5	2092	UMHMNP697299211	−8.2	−8.4
1983	UMHMNP351198088	−8.2	−8.5	2038	UMHMNP334491268	−8.2	−8.5	2093	UMHMNP97190460	−10.0	−8.4
1984	UMHMNP35339710	−8.4	−8.5	2039	UMHMNP614748639	−8.2	−8.5	2094	UMHMNP124689641	−9.2	−8.4
1985	UMHMNP35498670	−8.9	−8.5	2040	UMHMNP62820342	−9.9	−8.5	2095	UMHMNP12771721	−9.0	−8.4
1986	UMHMNP51847826	−9.8	−8.5	2041	UMHMNP664987126	−9.9	−8.5	2096	UMHMNP129704832	−8.8	−8.4
1987	UMHMNP61897889	−8.1	−8.5	2042	UMHMNP69672679	−8.1	−8.5	2097	UMHMNP139579296	−8.6	−8.4
1988	UMHMNP61949675	−9.0	−8.5	2043	UMHMNP75578653	−9.4	−8.5	2098	UMHMNP141672062	−8.6	−8.4
1989	UMHMNP62820331	−9.5	−8.5	2044	UMHMNP81575760	−9.3	−8.5	2099	UMHMNP144967851	−8.9	−8.4
1990	UMHMNP74185094	−9.7	−8.5	2045	UMHMNP876756579	−8.4	−8.5	2100	UMHMNP216770364	−8.6	−8.4

Table S1. *Continued.*

No.	Compound Name/Code	Docking Score (kcal/mol)		No.	Compound Name/Code	Docking Score (kcal/mol)		No.	Compound Name/Code	Docking Score (kcal/mol)	
		Fast	Mod.			Fast	Mod.			Fast	Mod.
2101	UMHMNP225779748	−8.5	−8.4	2156	UMHMNP105372814	−9.2	−8.4	2211	UMHMNP143572194	−9.5	−8.4
2102	UMHMNP33300026	−8.2	−8.4	2157	UMHMNP133056652	−8.6	−8.4	2212	UMHMNP157000776	−9.5	−8.4
2103	UMHMNP352674330	−8.1	−8.4	2158	UMHMNP134985061	−8.1	−8.4	2213	UMHMNP250147547	−8.7	−8.4
2104	UMHMNP6147439	−10.4	−8.4	2159	UMHMNP151890890	−8.3	−8.4	2214	UMHMNP35454945	−9.7	−8.4
2105	UMHMNP69819825	−8.3	−8.4	2160	UMHMNP157622521	−8.3	−8.4	2215	UMHMNP41336656	−9.3	−8.4
2106	UMHMNP74145725	−8.4	−8.4	2161	UMHMNP158200103	−9.5	−8.4	2216	UMHMNP425381277	−8.3	−8.4
2107	UMHMNP882524927	−8.4	−8.4	2162	UMHMNP287101637	−8.2	−8.4	2217	UMHMNP452956751	−8.8	−8.4
2108	UMHMNP99631239	−8.5	−8.4	2163	UMHMNP52498938	−9.0	−8.4	2218	UMHMNP53402189	−8.9	−8.4
2109	UMHMNP109152401	−9.2	−8.4	2164	UMHMNP58220	−9.2	−8.4	2219	UMHMNP54602163	−9.8	−8.4
2110	UMHMNP123498022	−9.0	−8.4	2165	UMHMNP65556583	−8.6	−8.4	2220	UMHMNP70206916	−8.5	−8.4
2111	UMHMNP138264489	−8.2	−8.4	2166	UMHMNP67528341	−8.6	−8.4	2221	UMHMNP799808369	−8.7	−8.4
2112	UMHMNP147217194	−8.8	−8.4	2167	UMHMNP746655958	−10.2	−8.4	2222	UMHMNP852469240	−9.8	−8.4
2113	UMHMNP149379260	−10.4	−8.4	2168	UMHMNP777859004	−9.2	−8.4	2223	UMHMNP858950546	−9.6	−8.4
2114	UMHMNP178326540	−9.1	−8.4	2169	UMHMNP851369863	−9.8	−8.4	2224	UMHMNP87532330	−10.8	−8.4
2115	UMHMNP194856365	−8.8	−8.4	2170	UMHMNP104900665	−10.0	−8.4	2225	UMHMNP97190302	−8.9	−8.4
2116	UMHMNP202932147	−8.3	−8.4	2171	UMHMNP106001289	−8.2	−8.4	2226	UMHMNP108335042	−8.3	−8.3
2117	UMHMNP211486134	−10.0	−8.4	2172	UMHMNP119770115	−8.5	−8.4	2227	UMHMNP111139696	−8.4	−8.3
2118	UMHMNP220866400	−9.3	−8.4	2173	UMHMNP138039335	−8.2	−8.4	2228	UMHMNP114318217	−8.3	−8.3
2119	UMHMNP246516370	−9.7	−8.4	2174	UMHMNP147318410	−8.3	−8.4	2229	UMHMNP123483205	−9.3	−8.3
2120	UMHMNP338949125	−8.9	−8.4	2175	UMHMNP152517798	−11.1	−8.4	2230	UMHMNP134455939	−9.4	−8.3
2121	UMHMNP719285026	−9.4	−8.4	2176	UMHMNP153660232	−8.1	−8.4	2231	UMHMNP145427709	−8.5	−8.3
2122	UMHMNP78798080	−8.7	−8.4	2177	UMHMNP157403322	−9.0	−8.4	2232	UMHMNP175861811	−8.4	−8.3
2123	UMHMNP81575113	−9.0	−8.4	2178	UMHMNP17942084	−9.0	−8.4	2233	UMHMNP19588375	−8.8	−8.3
2124	UMHMNP84323273	−9.1	−8.4	2179	UMHMNP209969391	−9.5	−8.4	2234	UMHMNP28032544	−8.2	−8.3
2125	UMHMNP117569434	−8.3	−8.4	2180	UMHMNP220924516	−9.1	−8.4	2235	UMHMNP39707545	−8.2	−8.3
2126	UMHMNP132342746	−8.2	−8.4	2181	UMHMNP245511346	−8.5	−8.4	2236	UMHMNP52239686	−8.3	−8.3
2127	UMHMNP134029451	−9.8	−8.4	2182	UMHMNP325691336	−9.0	−8.4	2237	UMHMNP96627359	−8.7	−8.3
2128	UMHMNP135474098	−8.2	−8.4	2183	UMHMNP359415706	−8.3	−8.4	2238	UMHMNP106463756	−9.0	−8.3
2129	UMHMNP144398243	−8.7	−8.4	2184	UMHMNP6094355	−9.8	−8.4	2239	UMHMNP114719572	−9.5	−8.3
2130	UMHMNP163496262	−9.1	−8.4	2185	UMHMNP61515262	−8.4	−8.4	2240	UMHMNP117021053	−8.3	−8.3
2131	UMHMNP179900982	−8.3	−8.4	2186	UMHMNP84473347	−8.9	−8.4	2241	UMHMNP157207891	−8.1	−8.3
2132	UMHMNP22465641	−9.9	−8.4	2187	UMHMNP858950524	−8.1	−8.4	2242	UMHMNP208708229	−9.1	−8.3
2133	UMHMNP25612593	−9.9	−8.4	2188	UMHMNP866403772	−9.6	−8.4	2243	UMHMNP64687838	−8.3	−8.3
2134	UMHMNP329050206	−8.1	−8.4	2189	UMHMNP890028316	−8.8	−8.4	2244	UMHMNP78697560	−8.9	−8.3
2135	UMHMNP3836019	−9.2	−8.4	2190	UMHMNP95513669	−8.8	−8.4	2245	UMHMNP89837989	−9.9	−8.3
2136	UMHMNP38623960	−8.7	−8.4	2191	UMHMNP103538552	−8.5	−8.4	2246	UMHMNP122271414	−9.1	−8.3
2137	UMHMNP51231277	−8.3	−8.4	2192	UMHMNP116079573	−10.4	−8.4	2247	UMHMNP133883097	−8.3	−8.3
2138	UMHMNP566289	−8.2	−8.4	2193	UMHMNP123116030	−8.7	−8.4	2248	UMHMNP145038568	−8.3	−8.3
2139	UMHMNP63015894	−8.3	−8.4	2194	UMHMNP129257938	−10.0	−8.4	2249	UMHMNP149415827	−8.1	−8.3
2140	UMHMNP72241328	−8.4	−8.4	2195	UMHMNP132618689	−9.3	−8.4	2250	UMHMNP174286170	−8.3	−8.3
2141	UMHMNP123231470	−8.7	−8.4	2196	UMHMNP146555819	−8.9	−8.4	2251	UMHMNP216973078	−9.3	−8.3
2142	UMHMNP125034084	−9.5	−8.4	2197	UMHMNP178176755	−8.0	−8.4	2252	UMHMNP267407249	−9.5	−8.3
2143	UMHMNP152833595	−8.3	−8.4	2198	UMHMNP182806128	−9.5	−8.4	2253	UMHMNP302324590	−9.4	−8.3
2144	UMHMNP321847114	−9.7	−8.4	2199	UMHMNP198481991	−8.3	−8.4	2254	UMHMNP5255152	−9.2	−8.3
2145	UMHMNP448264640	−8.4	−8.4	2200	UMHMNP306971062	−8.7	−8.4	2255	UMHMNP592533874	−8.5	−8.3
2146	UMHMNP63555486	−8.4	−8.4	2201	UMHMNP33646796	−8.1	−8.4	2256	UMHMNP66648531	−8.5	−8.3
2147	UMHMNP74364106	−8.4	−8.4	2202	UMHMNP393828325	−8.8	−8.4	2257	UMHMNP70214914	−9.2	−8.3
2148	UMHMNP76343936	−8.3	−8.4	2203	UMHMNP72016332	−8.7	−8.4	2258	UMHMNP98155170	−9.1	−8.3
2149	UMHMNP813436418	−8.5	−8.4	2204	UMHMNP864685949	−8.3	−8.4	2259	UMHMNP107610004	−9.4	−8.3
2150	UMHMNP81575022	−9.2	−8.4	2205	UMHMNP88526732	−8.9	−8.4	2260	UMHMNP160324947	−9.3	−8.3
2151	UMHMNP83481236	−8.5	−8.4	2206	UMHMNP97560708	−8.7	−8.4	2261	UMHMNP182801623	−8.4	−8.3
2152	UMHMNP853236	−8.6	−8.4	2207	UMHMNP102977764	−9.2	−8.4	2262	UMHMNP191480350	−9.6	−8.3
2153	UMHMNP86748290	−8.3	−8.4	2208	UMHMNP120853170	−8.4	−8.4	2263	UMHMNP264618180	−8.6	−8.3
2154	UMHMNP95513603	−10.1	−8.4	2209	UMHMNP121994501	−8.7	−8.4	2264	UMHMNP487016988	−8.9	−8.3
2155	UMHMNP97915907	−8.6	−8.4	2210	UMHMNP134029440	−9.7	−8.4	2265	UMHMNP69672668	−8.3	−8.3

Table S1. *Continued.*

No.	Compound Name/Code	Docking Score (kcal/mol)		No.	Compound Name/Code	Docking Score (kcal/mol)		No.	Compound Name/Code	Docking Score (kcal/mol)	
		Fast	Mod.			Fast	Mod.			Fast	Mod.
2266	UMHMNP876754766	−8.3	−8.3	2321	UMHMNP112663919	−9.3	−8.3	2376	UMHMNP29478420	−9.0	−8.2
2267	UMHMNP95388048_	−10.1	−8.3	2322	UMHMNP115178535	−9.1	−8.3	2377	UMHMNP329025152	−8.3	−8.2
2268	UMHMNP155944277	−9.5	−8.3	2323	UMHMNP121923975	−10.1	−8.3	2378	UMHMNP349147280	−9.3	−8.2
2269	UMHMNP65717897	−8.0	−8.3	2324	UMHMNP125482271	−8.8	−8.3	2379	UMHMNP437651064	−8.4	−8.2
2270	UMHMNP70142880	−8.1	−8.3	2325	UMHMNP139220181	−8.1	−8.3	2380	UMHMNP76958673	−8.3	−8.2
2271	UMHMNP81275812	−8.6	−8.3	2326	UMHMNP139953325	−8.4	−8.3	2381	UMHMNP85733682	−8.9	−8.2
2272	UMHMNP873202176	−8.9	−8.3	2327	UMHMNP142780312	−8.3	−8.3	2382	UMHMNP865668582	−8.1	−8.2
2273	UMHMNP90139201	−9.9	−8.3	2328	UMHMNP148000402	−9.0	−8.3	2383	UMHMNP129744247	−9.0	−8.2
2274	UMHMNP101968745	−9.5	−8.3	2329	UMHMNP169217372	−8.3	−8.3	2384	UMHMNP130221248	−9.1	−8.2
2275	UMHMNP151358473	−8.5	−8.3	2330	UMHMNP186803305	−8.2	−8.3	2385	UMHMNP156953854	−9.2	−8.2
2276	UMHMNP156280921	−10.4	−8.3	2331	UMHMNP199439753	−8.7	−8.3	2386	UMHMNP158734259	−8.9	−8.2
2277	UMHMNP159934164	−8.8	−8.3	2332	UMHMNP217478723	−8.6	−8.3	2387	UMHMNP194020434	−8.1	−8.2
2278	UMHMNP183114887	−8.1	−8.3	2333	UMHMNP32469800	−8.7	−8.3	2388	UMHMNP215440725	−9.6	−8.2
2279	UMHMNP223130618	−8.9	−8.3	2334	UMHMNP385793373	−8.2	−8.3	2389	UMHMNP287965279	−8.5	−8.2
2280	UMHMNP301842580	−8.3	−8.3	2335	UMHMNP62008111	−8.4	−8.3	2390	UMHMNP77761547	−8.9	−8.2
2281	UMHMNP485817616	−9.0	−8.3	2336	UMHMNP70165445	−8.6	−8.3	2391	UMHMNP874359418	−8.1	−8.2
2282	UMHMNP615285319	−8.3	−8.3	2337	UMHMNP71751774	−8.7	−8.3	2392	UMHMNP106009909	−8.6	−8.2
2283	UMHMNP62504614	−8.8	−8.3	2338	UMHMNP73723407	−9.2	−8.3	2393	UMHMNP123941582	−8.4	−8.2
2284	UMHMNP75921903	−8.2	−8.3	2339	UMHMNP77136620	−9.7	−8.3	2394	UMHMNP133293897	−9.8	−8.2
2285	UMHMNP761409249	−8.9	−8.3	2340	UMHMNP84164863	−8.6	−8.3	2395	UMHMNP161897638	−9.2	−8.2
2286	UMHMNP77394026	−9.3	−8.3	2341	UMHMNP89199917	−8.3	−8.3	2396	UMHMNP175861844	−8.6	−8.2
2287	UMHMNP862286724	−8.3	−8.3	2342	UMHMNP102396167	−10.1	−8.2	2397	UMHMNP188558976	−8.7	−8.2
2288	UMHMNP876754788	−9.5	−8.3	2343	UMHMNP109894113	−8.1	−8.2	2398	UMHMNP222960909	−9.0	−8.2
2289	UMHMNP122876486	−9.6	−8.3	2344	UMHMNP118974020	−8.2	−8.2	2399	UMHMNP244295645	−8.6	−8.2
2290	UMHMNP145075010	−8.1	−8.3	2345	UMHMNP137761172	−8.8	−8.2	2400	UMHMNP255830274	−8.2	−8.2
2291	UMHMNP189456040	−8.3	−8.3	2346	UMHMNP186144092	−8.3	−8.2	2401	UMHMNP486992272	−9.1	−8.2
2292	UMHMNP193816716	−8.3	−8.3	2347	UMHMNP215035817	−8.5	−8.2	2402	UMHMNP549502978	−8.7	−8.2
2293	UMHMNP213824307	−11.3	−8.3	2348	UMHMNP329040815	−8.1	−8.2	2403	UMHMNP58735641	−9.4	−8.2
2294	UMHMNP247921820	−8.8	−8.3	2349	UMHMNP49759220	−8.9	−8.2	2404	UMHMNP73723394	−8.1	−8.2
2295	UMHMNP249731348	−8.8	−8.3	2350	UMHMNP65079494	−8.6	−8.2	2405	UMHMNP88514049	−8.4	−8.2
2296	UMHMNP287101648	−8.7	−8.3	2351	UMHMNP67970	−9.6	−8.2	2406	UMHMNP89398130	−10.1	−8.2
2297	UMHMNP539824878	−8.5	−8.3	2352	UMHMNP69809365	−8.8	−8.2	2407	UMHMNP122540298	−8.3	−8.2
2298	UMHMNP77136835	−8.9	−8.3	2353	UMHMNP78835082	−9.2	−8.2	2408	UMHMNP135048576	−8.3	−8.2
2299	UMHMNP781646853	−8.2	−8.3	2354	UMHMNP83995066	−8.4	−8.2	2409	UMHMNP146345735	−8.4	−8.2
2300	UMHMNP105708767	−9.2	−8.3	2355	UMHMNP88428879	−8.8	−8.2	2410	UMHMNP174232418	−9.3	−8.2
2301	UMHMNP181229196	−9.5	−8.3	2356	UMHMNP105880102	−8.8	−8.2	2411	UMHMNP26612486	−9.9	−8.2
2302	UMHMNP314739952	−8.6	−8.3	2357	UMHMNP109894260	−9.5	−8.2	2412	UMHMNP325691461	−10.0	−8.2
2303	UMHMNP403805743	−8.3	−8.3	2358	UMHMNP121295112	−9.5	−8.2	2413	UMHMNP852469284	−8.9	−8.2
2304	UMHMNP54602152	−9.3	−8.3	2359	UMHMNP125282124	−8.3	−8.2	2414	UMHMNP890041697	−8.7	−8.2
2305	UMHMNP62824082	−8.3	−8.3	2360	UMHMNP130799381	−10.8	−8.2	2415	UMHMNP156848689	−8.6	−8.2
2306	UMHMNP705279727	−8.4	−8.3	2361	UMHMNP132911536	−8.1	−8.2	2416	UMHMNP191927147	−9.3	−8.2
2307	UMHMNP78853028	−8.9	−8.3	2362	UMHMNP148101502	−8.7	−8.2	2417	UMHMNP196403937	−8.1	−8.2
2308	UMHMNP100015165	−8.3	−8.3	2363	UMHMNP158930451	−9.9	−8.2	2418	UMHMNP29706965	−8.7	−8.2
2309	UMHMNP108787560	−8.5	−8.3	2364	UMHMNP176181876	−9.0	−8.2	2419	UMHMNP310396704	−8.4	−8.2
2310	UMHMNP121825421	−8.6	−8.3	2365	UMHMNP180001201	−8.7	−8.2	2420	UMHMNP340156185	−8.1	−8.2
2311	UMHMNP125236591	−8.5	−8.3	2366	UMHMNP189514849	−10.1	−8.2	2421	UMHMNP349147291	−9.1	−8.2
2312	UMHMNP135820790	−9.0	−8.3	2367	UMHMNP193411744	−9.4	−8.2	2422	UMHMNP813461200	−9.3	−8.2
2313	UMHMNP142755075	−8.8	−8.3	2368	UMHMNP39707567	−8.5	−8.2	2423	UMHMNP90468738	−8.5	−8.2
2314	UMHMNP166318861	−9.3	−8.3	2369	UMHMNP70497142	−8.6	−8.2	2424	UMHMNP103955755	−9.9	−8.2
2315	UMHMNP247115293	−8.9	−8.3	2370	UMHMNP96845555	−8.6	−8.2	2425	UMHMNP116079562	−8.3	−8.2
2316	UMHMNP481643496	−8.2	−8.3	2371	UMHMNP97764357	−8.5	−8.2	2426	UMHMNP132244383	−8.2	−8.2
2317	UMHMNP648883312	−8.4	−8.3	2372	UMHMNP115890590	−8.7	−8.2	2427	UMHMNP145427732	−8.1	−8.2
2318	UMHMNP80388449	−8.6	−8.3	2373	UMHMNP132541845	−8.4	−8.2	2428	UMHMNP149378574	−9.9	−8.2
2319	UMHMNP111268645	−8.5	−8.3	2374	UMHMNP144676379	−9.1	−8.2	2429	UMHMNP169181344	−8.3	−8.2
2320	UMHMNP112570873	−8.7	−8.3	2375	UMHMNP175861888	−8.3	−8.2	2430	UMHMNP170475135	−9.0	−8.2

Table S1. *Continued.*

No.	Compound Name/Code	Docking Score (kcal/mol)		No.	Compound Name/Code	Docking Score (kcal/mol)		No.	Compound Name/Code	Docking Score (kcal/mol)	
		Fast	Mod.			Fast	Mod.			Fast	Mod.
2431	UMHMNP244295634	−8.1	−8.2	2486	UMHMNP155885657	−8.2	−8.1	2541	UMHMNP330846929	−8.6	−8.1
2432	UMHMNP5996225	−9.5	−8.2	2487	UMHMNP19369643	−8.5	−8.1	2542	UMHMNP80902358	−8.1	−8.1
2433	UMHMNP74799567	−9.1	−8.2	2488	UMHMNP259194939	−9.1	−8.1	2543	UMHMNP115952255	−8.8	−8.0
2434	UMHMNP871332079	−8.1	−8.2	2489	UMHMNP27975195	−10.3	−8.1	2544	UMHMNP124512454	−10.4	−8.0
2435	UMHMNP100814662	−8.1	−8.2	2490	UMHMNP306971142	−8.2	−8.1	2545	UMHMNP132911503	−9.0	−8.0
2436	UMHMNP129932805	−8.8	−8.2	2491	UMHMNP481643485	−9.0	−8.1	2546	UMHMNP146396249	−8.4	−8.0
2437	UMHMNP145398670	−8.5	−8.2	2492	UMHMNP66082302	−8.4	−8.1	2547	UMHMNP150998995	−9.2	−8.0
2438	UMHMNP15979767	−8.2	−8.2	2493	UMHMNP70546644	−8.9	−8.1	2548	UMHMNP157622598	−9.1	−8.0
2439	UMHMNP167172852	−11.1	−8.2	2494	UMHMNP88514061	−9.0	−8.1	2549	UMHMNP200437689	−9.0	−8.0
2440	UMHMNP171528044	−8.9	−8.2	2495	UMHMNP145398590	−8.5	−8.1	2550	UMHMNP37717025	−8.6	−8.0
2441	UMHMNP174630092	−9.0	−8.2	2496	UMHMNP154554902	−8.3	−8.1	2551	UMHMNP58957	−8.5	−8.0
2442	UMHMNP176328500	−8.1	−8.2	2497	UMHMNP155850901	−8.7	−8.1	2552	UMHMNP74185038	−9.5	−8.0
2443	UMHMNP251907194	−9.2	−8.2	2498	UMHMNP164301308	−8.3	−8.1	2553	UMHMNP74839819	−8.2	−8.0
2444	UMHMNP3148092	−8.5	−8.2	2499	UMHMNP433717716	−8.3	−8.1	2554	UMHMNP80388483	−8.7	−8.0
2445	UMHMNP57765749	−8.7	−8.2	2500	UMHMNP864685961	−8.7	−8.1	2555	UMHMNP112515410	−10.4	−8.0
2446	UMHMNP6055501	−8.3	−8.2	2501	UMHMNP99957252	−8.3	−8.1	2556	UMHMNP113351750	−8.1	−8.0
2447	UMHMNP68835916	−8.1	−8.2	2502	UMHMNP114728073	−8.7	−8.1	2557	UMHMNP125236604	−8.5	−8.0
2448	UMHMNP75088812	−9.7	−8.2	2503	UMHMNP146555831	−8.6	−8.1	2558	UMHMNP129602137	−10.0	−8.0
2449	UMHMNP80322107	−8.3	−8.2	2504	UMHMNP156280943	−8.7	−8.1	2559	UMHMNP184301915	−8.9	−8.0
2450	UMHMNP847450742	−8.6	−8.2	2505	UMHMNP159518793	−8.2	−8.1	2560	UMHMNP282118858	−10.0	−8.0
2451	UMHMNP861434142	−8.4	−8.2	2506	UMHMNP179669331	−8.9	−8.1	2561	UMHMNP282524623	−8.8	−8.0
2452	UMHMNP104387135	−8.8	−8.2	2507	UMHMNP232922235	−10.0	−8.1	2562	UMHMNP33281811	−9.3	−8.0
2453	UMHMNP135048554	−9.0	−8.2	2508	UMHMNP460060224	−9.6	−8.1	2563	UMHMNP108907240	−10.1	−8.0
2454	UMHMNP157799150	−8.8	−8.2	2509	UMHMNP108605512	−8.2	−8.1	2564	UMHMNP90162268	−8.8	−8.0
2455	UMHMNP184305688	−9.9	−8.2	2510	UMHMNP265652991	−8.6	−8.1	2565	UMHMNP102396247	−8.5	−8.0
2456	UMHMNP185839218	−8.7	−8.2	2511	UMHMNP478364248	−9.5	−8.1	2566	UMHMNP325691483	−9.4	−8.0
2457	UMHMNP193411766	−9.2	−8.2	2512	UMHMNP625413356	−8.1	−8.1	2567	UMHMNP331970059	−8.6	−8.0
2458	UMHMNP353796211	−8.3	−8.2	2513	UMHMNP74364117	−9.8	−8.1	2568	UMHMNP124596652	−8.3	−8.0
2459	UMHMNP663154572	−8.4	−8.2	2514	UMHMNP76984913	−8.1	−8.1	2569	UMHMNP132621831	−9.2	−8.0
2460	UMHMNP116302364	−8.5	−8.1	2515	UMHMNP78173883	−8.5	−8.1	2570	UMHMNP88899610	−8.7	−8.0
2461	UMHMNP133401124	−8.6	−8.1	2516	UMHMNP96426925	−8.3	−8.1	2571	UMHMNP141544629	−9.0	−8.0
2462	UMHMNP155210529	−8.9	−8.1	2517	UMHMNP110042289	−8.5	−8.1	2572	UMHMNP55826512	−8.4	−8.0
2463	UMHMNP165171244	−8.5	−8.1	2518	UMHMNP133883086	−8.8	−8.1	2573	UMHMNP59048816	−9.0	−8.0
2464	UMHMNP233607631	−9.6	−8.1	2519	UMHMNP197234198	−8.6	−8.1	2574	UMHMNP132410390	−8.3	−7.9
2465	UMHMNP511262	−8.4	−8.1	2520	UMHMNP199165872	−8.6	−8.1	2575	UMHMNP99102406	−8.2	−7.9
2466	UMHMNP85754707	−8.1	−8.1	2521	UMHMNP31665324	−8.4	−8.1	2576	UMHMNP126026582	−8.1	−7.9
2467	UMHMNP866228946	−8.1	−8.1	2522	UMHMNP62008155	−8.5	−8.1	2577	UMHMNP180001212	−8.3	−7.9
2468	UMHMNP113023715	−8.8	−8.1	2523	UMHMNP690627613	−9.4	−8.1	2578	UMHMNP189580083	−8.9	−7.9
2469	UMHMNP114763518	−10.3	−8.1	2524	UMHMNP94359656	−9.4	−8.1	2579	UMHMNP200437645	−9.4	−7.9
2470	UMHMNP117229411	−8.5	−8.1	2525	UMHMNP120484802	−8.7	−8.1	2580	UMHMNP181034979	−9.4	−7.9
2471	UMHMNP119979769	−8.3	−8.1	2526	UMHMNP128700856	−8.2	−8.1	2581	UMHMNP100046035	−8.8	−7.9
2472	UMHMNP129744156	−8.5	−8.1	2527	UMHMNP145131	−8.5	−8.1	2582	UMHMNP157002409	−8.1	−7.9
2473	UMHMNP135048601	−9.0	−8.1	2528	UMHMNP173485819	−9.4	−8.1	2583	UMHMNP863638102	−9.1	−7.9
2474	UMHMNP137571303	−8.8	−8.1	2529	UMHMNP238431760	−8.9	−8.1	2584	UMHMNP68857443	−8.3	−7.9
2475	UMHMNP137836658	−8.3	−8.1	2530	UMHMNP356566842	−8.7	−8.1	2585	UMHMNP114076516	−8.3	−7.8
2476	UMHMNP192222176	−9.5	−8.1	2531	UMHMNP479201575	−9.6	−8.1	2586	UMHMNP122548043	−10.2	−7.8
2477	UMHMNP207918954	−8.5	−8.1	2532	UMHMNP67240431	−9.0	−8.1	2587	UMHMNP189388827	−8.8	−7.8
2478	UMHMNP252733407	−8.8	−8.1	2533	UMHMNP68042999	−8.4	−8.1	2588	UMHMNP338949114	−8.3	−7.8
2479	UMHMNP73731347	−8.3	−8.1	2534	UMHMNP847450719	−8.9	−8.1	2589	UMHMNP496909763	−9.2	−7.8
2480	UMHMNP80442780	−9.4	−8.1	2535	UMHMNP130322246	−8.6	−8.1	2590	UMHMNP106009727	−8.2	−7.8
2481	UMHMNP81920185	−8.2	−8.1	2536	UMHMNP250686874	−8.1	−8.1	2591	UMHMNP121379484	−8.8	−7.8
2482	UMHMNP890028305	−9.5	−8.1	2537	UMHMNP71302266	−8.8	−8.1	2592	UMHMNP109979160	−8.2	−7.8
2483	UMHMNP132160442	−8.3	−8.1	2538	UMHMNP74804038	−10.0	−8.1	2593	UMHMNP180048106	−8.5	−7.8
2484	UMHMNP132911467	−8.3	−8.1	2539	UMHMNP114094336	−9.4	−8.1	2594	UMHMNP52665744	−9.2	−7.8
2485	UMHMNP136094365	−8.6	−8.1	2540	UMHMNP127875609	−9.1	−8.1	2595	UMHMNP145398681	−8.4	−7.8

Table S1. *Continued.*

No.	Compound Name/Code	Docking Score (kcal/mol)		No.	Compound Name/Code	Docking Score (kcal/mol)		No.	Compound Name/Code	Docking Score (kcal/mol)	
		Fast	Mod.			Fast	Mod.			Fast	Mod.
2596	UMHMNP405274159	−8.4	−7.8	2626	UMHMNP114216874	−8.1	−7.5	2657	UMHMNP80764198	−8.1	−7.1
2597	UMHMNP164727337	−8.1	−7.8	2627	UMHMNP180965257	−8.8	−7.5	2658	UMHMNP96820132	−9.2	−7.0
2598	UMHMNP620604413	−9.6	−7.8	2628	UMHMNP132564988	−8.2	−7.4	2659	UMHMNP168482380	−9.2	−7.0
2599	UMHMNP95387476	−8.7	−7.8	2629	UMHMNP620604220	−8.6	−7.4	2660	UMHMNP147663783	−9.0	−7.0
2600	UMHMNP405283376	−8.7	−7.8	2630	UMHMNP97730988	−8.5	−7.4	2661	UMHMNP115787967	−8.6	−7.0
2601	UMHMNP1406662	−8.1	−7.7	2632	UMHMNP856414625	−9.4	−7.4	2662	UMHMNP53755569	−8.1	−7.0
2602	UMHMNP174545789	−8.2	−7.7	2633	UMHMNP130829311	−8.6	−7.4	2663	UMHMNP150351238	−8.6	−6.9
2603	UMHMNP13394351	−8.7	−7.7	2634	UMHMNP176226576	−8.7	−7.4	2664	UMHMNP126596041	−8.8	−6.9
2604	UMHMNP139579310	−8.2	−7.7	2635	UMHMNP196874109	−8.6	−7.4	2665	UMHMNP207683703	−8.8	−6.9
2605	UMHMNP90332722	−8.6	−7.7	2636	UMHMNP114216852	−8.3	−7.4	2666	UMHMNP479249304	−9.4	−6.9
2606	UMHMNP81254	−8.8	−7.7	2637	UMHMNP115178524	−8.2	−7.4	2667	UMHMNP161897718	−8.7	−6.8
2607	UMHMNP81575102	−8.8	−7.7	2638	UMHMNP15979712	−8.6	−7.4	2668	UMHMNP107168605	−9.3	−6.8
2608	UMHMNP174232361	−9.0	−7.7	2639	UMHMNP174630081	−8.8	−7.3	2669	UMHMNP152606616	−8.3	−6.8
2609	UMHMNP99458045	−9.3	−7.7	2640	UMHMNP227597435	−8.1	−7.3	2670	UMHMNP70363892	−8.5	−6.7
2610	UMHMNP170661413	−9.4	−7.7	2641	UMHMNP153212841	−8.1	−7.3	2671	UMHMNP681225956	−9.0	−6.6
2611	UMHMNP314740006	−8.1	−7.7	2642	UMHMNP190510862	−9.5	−7.3	2672	UMHMNP115178546	−8.1	−6.6
2612	UMHMNP168434239	−9.3	−7.6	2643	UMHMNP130170079	−10.4	−7.3	2673	UMHMNP109152365	−8.5	−6.5
2613	UMHMNP107585455	−8.8	−7.6	2644	UMHMNP97068309	−8.3	−7.3	2674	UMHMNP799246869	−9.3	−6.4
2614	UMHMNP160581135	−8.2	−7.6	2645	UMHMNP160638120	−8.5	−7.3	2675	UMHMNP212502879	−8.4	−6.4
2615	UMHMNP136734935	−8.2	−7.6	2646	UMHMNP108663770	−8.4	−7.2	2676	UMHMNP77136631	−9.1	−6.4
2616	UMHMNP92279920	−8.3	−7.6	2647	UMHMNP205323144	−8.4	−7.2	2677	UMHMNP856414589	−8.8	−6.3
2617	UMHMNP148022382	−9.1	−7.6	2648	UMHMNP75872683	−8.6	−7.2	2678	UMHMNP383898064	−9.8	−6.3
2618	UMHMNP105064344	−8.9	−7.6	2649	UMHMNP188405187	−8.6	−7.2	2679	UMHMNP266352367	−8.8	−6.2
2619	UMHMNP123167011	−8.3	−7.6	2650	UMHMNP168075136	−9.0	−7.2	2680	UMHMNP102209778	−8.6	−6.1
2620	UMHMNP20298866	−9.0	−7.6	2651	UMHMNP135531805	−8.5	−7.2	2681	UMHMNP126675523	−10.2	−6.0
2621	UMHMNP163318794	−8.5	−7.5	2652	UMHMNP136707692	−9.9	−7.2	2682	UMHMNP168433929	−8.9	−5.3
2622	UMHMNP179985616	−8.1	−7.5	2653	UMHMNP28979295	−8.8	−7.1	2683	UMHMNP189819190	−8.0	−5.3
2623	UMHMNP224784478	−8.6	−7.5	2654	UMHMNP111466672	−9.7	−7.1	2684	UMHMNP681225967	−8.7	−5.0
2624	UMHMNP125111695	−11.2	−7.5	2655	UMHMNP13408565	−8.6	−7.1	2685	UMHMNP697299200	−8.1	−5.0
2625	UMHMNP128269692	−8.9	−7.5	2656	UMHMNP147663794	−8.3	−7.1	2686	UMHMNP73573178	−8.1	−5.0

[a]Data sorted according to stage II docking scores.

Table S2. Estimated fast, moderate, and expensive docking scores for XF7 and the top 1092 potent MNPs compounds within the 3CL^{pro} binding pocket.

No.	Compound Name/Code	Docking Score (kcal/mol)			No.	Compound Name/Code	Docking Score (kcal/mol)		
		Fast	Moderate	Expensive			Fast	Moderate	Expensive
	XF7	−8.1	−9.2	−9.5	55	UMHMNP143049165	−8.9	−10.6	−11.0
1	UMHMNP1403367	−11.7	−12.2	−13.4	56	UMHMNP143049143	−8.4	−9.3	−11.0
2	UMHMNP101691127	−11.4	−12.1	−12.3	57	UMHMNP58670636	−9.5	−11.0	−11.0
3	UMHMNP791849666	−11.4	−11.9	−12.3	58	UMHMNP6673661	−9.6	−10.9	−10.9
4	UMHMNP14984668	−11.3	−11.7	−12.2	59	UMHMNP161470231	−8.9	−10.6	−10.9
5	UMHMNP143621754	−11.3	−11.7	−12.2	60	UMHMNP890041722	−10.8	−10.9	−10.9
6	UMHMNP148839036	−11.3	−11.6	−11.8	61	UMHMNP171674923	−8.9	−10.8	−10.9
7	UMHMNP133056072	−11.3	−11.6	−11.7	62	UMHMNP125329091	−10.9	−9.9	−10.9
8	UMHMNP386274857	−11.3	−11.6	−11.7	63	UMHMNP174630070	−10.9	−11.0	−10.9
9	UMHMNP133056094	−11.2	−11.5	−11.5	64	UMHMNP131467000	−10.7	−10.8	−10.8
10	UMHMNP26195584	−11.1	−11.5	−11.5	65	UMHMNP221163311	−9.6	−10.5	−10.8
11	UMHMNP874383707	−11.1	−11.5	−11.4	66	UMHMNP139765370	−8.1	−10.7	−10.8
12	UMHMNP221163300	−11.1	−11.4	−11.4	67	UMHMNP86708329	−10.6	−10.7	−10.8
13	UMHMNP109152387	−11.1	−11.4	−11.4	68	UMHMNP151606405	−9.4	−9.8	−10.8
14	UMHMNP156953912	−11.1	−11.4	−11.4	69	UMHMNP151078921	−8.8	−10.7	−10.8
15	UMHMNP5035303	−11.0	−11.4	−11.4	70	UMHMNP272458330	−9.2	−9.3	−10.7
16	UMHMNP706784727	−10.9	−11.3	−11.4	71	UMHMNP272458341	−10.0	−10.3	−10.7
17	UMHMNP116407160	−8.7	−9.5	−11.3	72	UMHMNP64421201	−8.8	−9.5	−10.7
18	UMHMNP100942743	−8.8	−11.2	−11.3	73	UMHMNP162830231	−8.8	−9.3	−10.7
19	UMHMNP100942754	−9.1	−10.7	−11.3	74	UMHMNP64548169	−9.3	−9.5	−10.7
20	UMHMNP105404840	−10.0	−10.1	−11.3	75	UMHMNP70139569	−8.2	−9.3	−10.7
21	UMHMNP131466972	−8.2	−9.3	−11.3	76	UMHMNP244157946	−8.6	−9.7	−10.7
22	UMHMNP147362376	−8.3	−9.3	−11.3	77	UMHMNP41410586	−8.4	−9.8	−10.7
23	UMHMNP149444928	−9.1	−9.4	−11.3	78	UMHMNP2497747	−9.3	−9.3	−10.7
24	UMHMNP149764341	−8.9	−9.6	−11.3	79	UMHMNP852872925	−8.4	−9.7	−10.7
25	UMHMNP150050138	−10.7	−10.7	−11.3	80	UMHMNP116302386	−9.4	−9.5	−10.7
26	UMHMNP153212852	−8.4	−9.7	−11.3	81	UMHMNP124689652	−8.9	−9.4	−10.7
27	UMHMNP154205182	−9.9	−11.2	−11.3	82	UMHMNP4030926	−8.8	−9.3	−10.7
28	UMHMNP157207915	−9.2	−10.8	−11.3	83	UMHMNP351443759	−8.8	−9.9	−10.7
29	UMHMNP162830264	−9.5	−10.4	−11.3	84	UMHMNP74185049	−10.0	−10.1	−10.6
30	UMHMNP167172987	−8.4	−9.5	−11.3	85	UMHMNP152110093	−10.2	−9.3	−10.6
31	UMHMNP22033870	−8.6	−9.7	−11.3	86	UMHMNP165815816	−8.6	−9.8	−10.6
32	UMHMNP221169740	−8.4	−9.6	−11.3	87	UMHMNP444987637	−8.3	−9.9	−10.6
33	UMHMNP76915247	−9.6	−9.7	−11.3	88	UMHMNP6673683	−9.5	−9.7	−10.6
34	UMHMNP823808555	−8.4	−10.1	−11.3	89	UMHMNP745075278	−9.9	−10.0	−10.6
35	UMHMNP120685112	−8.2	−11.0	−11.3	90	UMHMNP129350181	−8.1	−9.3	−10.5
36	UMHMNP184885890	−8.2	−9.3	−11.3	91	UMHMNP741259858	−9.1	−9.3	−10.5
37	UMHMNP148010406	−8.9	−10.6	−11.3	92	UMHMNP827348072	−9.7	−9.7	−10.5
38	UMHMNP153212863	−8.2	−11.2	−11.2	93	UMHMNP244157957	−8.7	−11.2	−10.5
39	UMHMNP157622601	−8.5	−9.8	−11.2	94	UMHMNP253195403	−9.4	−9.5	−10.5
40	UMHMNP107503093	−8.2	−9.7	−11.2	95	UMHMNP150079951	−8.7	−9.3	−10.5
41	UMHMNP116303	−9.5	−9.4	−11.2	96	UMHMNP77982800	−10.9	−11.2	−10.5
42	UMHMNP53296836	−9.9	−9.3	−11.2	97	UMHMNP868395884	−9.1	−9.6	−10.5
43	UMHMNP176447964	−9.0	−9.4	−11.2	98	UMHMNP1058613	−9.5	−10.0	−10.5
44	UMHMNP119108360	−8.9	−9.7	−11.1	99	UMHMNP374819086	−8.9	−9.6	−10.5
45	UMHMNP393870332	−8.5	−9.3	−11.0	100	UMHMNP474395	−8.2	−9.9	−10.5
46	UMHMNP94203539	−9.9	−10.0	−11.0	101	UMHMNP70329281	−9.1	−10.1	−10.5
47	UMHMNP54369118	−9.0	−9.4	−11.0	102	UMHMNP142796314	−8.1	−10.7	−10.5
48	UMHMNP260436291	−9.5	−9.7	−11.0	103	UMHMNP290824474	−8.9	−9.5	−10.5
49	UMHMNP61369275	−8.7	−10.0	−11.0	104	UMHMNP552839802	−8.3	−9.3	−10.5
50	UMHMNP757976673	−9.7	−9.8	−11.0	105	UMHMNP862286702	−9.3	−9.7	−10.5
51	UMHMNP106231258	−9.7	−9.7	−11.0	106	UMHMNP2122987	−9.4	−9.4	−10.4
52	UMHMNP54028465	−9.9	−9.8	−11.0	107	UMHMNP73622238	−9.9	−10.1	−10.4
53	UMHMNP664992272	−9.0	−10.1	−11.0	108	UMHMNP386274868	−8.8	−10.3	−10.4
54	UMHMNP86748314	−8.1	−9.3	−11.0	109	UMHMNP745075245	−8.2	−9.3	−10.4

Table S2. *Continued.*

No.	Compound Name/Code	Docking Score (kcal/mol)			No.	Compound Name/Code	Docking Score (kcal/mol)		
		Fast	Moderate	Expensive			Fast	Moderate	Expensive
110	UMHMNP80981379	−9.2	−9.4	−10.4	166	UMHMNP82866339	−10.9	−11.2	−10.0
111	UMHMNP142755133	−8.5	−9.3	−10.4	167	UMHMNP83115473	−8.5	−9.5	−10.0
112	UMHMNP173792580	−8.6	−9.3	−10.4	168	UMHMNP84025321	−8.8	−10.1	−10.0
113	UMHMNP23518981	−8.7	−9.7	−10.4	169	UMHMNP85733795	−9.6	−9.3	−10.0
114	UMHMNP77944037	−9.2	−9.4	−10.4	170	UMHMNP244157968	−9.6	−9.3	−10.0
115	UMHMNP142755144	−9.9	−10.0	−10.4	171	UMHMNP81826495	−8.4	−9.3	−10.0
116	UMHMNP156953934	−10.9	−9.4	−10.4	172	UMHMNP168569184	−8.2	−9.3	−10.0
117	UMHMNP135091111	−8.3	−9.3	−10.4	173	UMHMNP19254694	−9.1	−9.3	−10.0
118	UMHMNP152845744	−9.1	−9.8	−10.4	174	UMHMNP70022718	−9.6	−10.2	−10.0
119	UMHMNP2418453	−8.1	−10.1	−10.4	175	UMHMNP74185129	−8.2	−9.3	−10.0
120	UMHMNP623167715	−8.4	−10.0	−10.4	176	UMHMNP83704103	−9.0	−9.5	−10.0
121	UMHMNP6377180	−8.2	−9.3	−10.4	177	UMHMNP110012185	−8.0	−9.5	−10.0
122	UMHMNP133613771	−8.4	−10.0	−10.4	178	UMHMNP253340293	−10.8	−11.2	−10.0
123	UMHMNP99528877	−10.4	−10.4	−10.4	179	UMHMNP75605855	−9.5	−9.8	−10.0
124	UMHMNP142796303	−9.7	−10.0	−10.4	180	UMHMNP80735064	−9.9	−10.0	−10.0
125	UMHMNP516869	−10.2	−10.5	−10.4	181	UMHMNP122143902	−9.4	−9.3	−10.0
126	UMHMNP109152376	−9.3	−9.3	−10.3	182	UMHMNP143380396	−8.7	−9.3	−10.0
127	UMHMNP126149904	−8.8	−9.8	−10.3	183	UMHMNP166797746	−8.6	−9.3	−10.0
128	UMHMNP890041675	−8.9	−9.3	−10.3	184	UMHMNP481141	−8.3	−9.4	−10.0
129	UMHMNP19698669	−8.8	−9.5	−10.3	185	UMHMNP63109193	−8.2	−9.5	−10.0
130	UMHMNP6901606	−8.9	−9.4	−10.3	186	UMHMNP6891356	−9.2	−9.3	−10.0
131	UMHMNP77643244	−9.6	−9.8	−10.3	187	UMHMNP123314216	−8.1	−10.0	−10.0
132	UMHMNP84223030	−8.8	−9.3	−10.3	188	UMHMNP196500762	−8.9	−9.8	−10.0
133	UMHMNP139765347	−10.0	−9.5	−10.3	189	UMHMNP50335030	−8.6	−9.3	−10.0
134	UMHMNP151109545	−8.6	−9.3	−10.3	190	UMHMNP160666546	−9.5	−10.9	−10.0
135	UMHMNP870095344	−9.8	−9.9	−10.3	191	UMHMNP104048171	−9.5	−9.9	−10.0
136	UMHMNP152130615	−9.0	−9.8	−10.3	192	UMHMNP201800615	−8.1	−9.9	−10.0
137	UMHMNP53216027	−8.9	−9.3	−10.3	193	UMHMNP58028232	−8.2	−10.0	−10.0
138	UMHMNP870535129	−9.9	−10.0	−10.3	194	UMHMNP221169762	−8.3	−9.6	−10.0
139	UMHMNP175992991	−9.3	−9.3	−10.3	195	UMHMNP260436279	−8.5	−9.3	−10.0
140	UMHMNP196500784	−8.5	−9.5	−10.2	196	UMHMNP94451215	−8.8	−9.3	−10.0
141	UMHMNP61843796	−9.1	−10.1	−10.2	197	UMHMNP104928305	−9.9	−10.1	−10.0
142	UMHMNP681227032	−8.2	−9.5	−10.2	198	UMHMNP221169739	−8.7	−10.1	−10.0
143	UMHMNP91283919	−9.9	−11.0	−10.2	199	UMHMNP244157979	−9.6	−10.5	−10.0
144	UMHMNP165815849	−9.5	−11.2	−10.2	200	UMHMNP862200495	−9.8	−9.3	−10.0
145	UMHMNP97614647	−9.3	−9.3	−10.2	201	UMHMNP134128168	−10.8	−10.4	−10.0
146	UMHMNP196403926	−8.9	−9.8	−10.2	202	UMHMNP162232395	−8.3	−9.3	−10.0
147	UMHMNP682808895	−9.0	−10.9	−10.2	203	UMHMNP54854921	−9.2	−9.3	−10.0
148	UMHMNP208927155	−8.9	−9.4	−10.2	204	UMHMNP55688443	−8.4	−9.3	−10.0
149	UMHMNP29782658	−9.5	−9.5	−10.2	205	UMHMNP123165800	−9.9	−9.5	−10.0
150	UMHMNP86105648	−10.8	−9.3	−10.2	206	UMHMNP128229641	−8.1	−9.3	−10.0
151	UMHMNP888041267	−8.9	−9.3	−10.1	207	UMHMNP23670942	−8.7	−9.9	−10.0
152	UMHMNP124727077	−9.7	−9.9	−10.1	208	UMHMNP474679	−8.3	−9.5	−10.0
153	UMHMNP149355759	−9.7	−9.8	−10.1	209	UMHMNP648428324	−8.5	−9.6	−10.0
154	UMHMNP155645515	−8.3	−9.9	−10.1	210	UMHMNP676271833	−9.9	−10.7	−10.0
155	UMHMNP72509616	−8.4	−9.7	−10.1	211	UMHMNP735278281	−9.2	−9.3	−10.0
156	UMHMNP116972931	−8.9	−9.4	−10.1	212	UMHMNP134131183	−8.9	−9.3	−10.0
157	UMHMNP114127785	−9.3	−9.3	−10.1	213	UMHMNP171864805	−8.2	−9.9	−10.0
158	UMHMNP115982202	−10.9	−10.4	−10.1	214	UMHMNP55688432	−8.1	−9.5	−10.0
159	UMHMNP133562499	−8.2	−10.5	−10.1	215	UMHMNP68520263	−9.6	−10.9	−10.0
160	UMHMNP40446068	−8.5	−9.9	−10.0	216	UMHMNP72007944	−9.4	−9.4	−10.0
161	UMHMNP53822983	−8.7	−9.3	−10.0	217	UMHMNP80375184	−8.9	−9.6	−10.0
162	UMHMNP57082367	−8.5	−10.0	−10.0	218	UMHMNP810685564	−9.1	−9.6	−10.0
163	UMHMNP118574735	−8.7	−9.7	−10.0	219	UMHMNP854203784	−8.9	−9.8	−10.0
164	UMHMNP22260464	−8.0	−9.7	−10.0	220	UMHMNP97649042	−8.3	−9.3	−10.0
165	UMHMNP405095945	−8.1	−9.9	−10.0	221	UMHMNP102396178	−10.1	−10.1	−10.0

Table S2. *Continued.*

No.	Compound Name/Code	Docking Score (kcal/mol)			No.	Compound Name/Code	Docking Score (kcal/mol)		
		Fast	Moderate	Expensive			Fast	Moderate	Expensive
222	UMHMNP28949668	−8.4	−10.0	−10.0	278	UMHMNP27065958	−9.4	−9.3	−9.9
223	UMHMNP80925060	−8.2	−9.8	−10.0	279	UMHMNP3903579	−8.8	−9.6	−9.9
224	UMHMNP83681792	−9.4	−9.6	−10.0	280	UMHMNP681260093	−9.5	−9.5	−9.9
225	UMHMNP858950400	−9.5	−9.6	−10.0	281	UMHMNP136762942	−9.2	−10.1	−9.9
226	UMHMNP105064300	−9.7	−9.8	−10.0	282	UMHMNP140429374	−9.7	−9.8	−9.9
227	UMHMNP151247697	−9.2	−9.3	−10.0	283	UMHMNP143572730	−8.7	−9.3	−9.9
228	UMHMNP53296847	−8.5	−9.3	−10.0	284	UMHMNP150050127	−9.0	−9.5	−9.9
229	UMHMNP137576002	−9.2	−9.5	−10.0	285	UMHMNP84582627	−9.4	−10.2	−9.9
230	UMHMNP176666834	−9.2	−9.6	−10.0	286	UMHMNP852469375	−8.4	−9.7	−9.9
231	UMHMNP57800585	−8.9	−9.3	−10.0	287	UMHMNP114820245	−9.5	−10.5	−9.9
232	UMHMNP832723736	−8.5	−10.2	−10.0	288	UMHMNP151890812	−9.6	−9.6	−9.9
233	UMHMNP117675191	−8.6	−9.3	−10.0	289	UMHMNP213547285	−8.7	−9.3	−9.9
234	UMHMNP145398603	−9.8	−9.9	−10.0	290	UMHMNP214483155	−8.8	−9.7	−9.9
235	UMHMNP185014599	−8.6	−9.3	−10.0	291	UMHMNP283176136	−9.6	−9.6	−9.9
236	UMHMNP29939977	−8.1	−9.3	−10.0	292	UMHMNP76758219	−10.2	−10.8	−9.9
237	UMHMNP50909869	−8.7	−9.3	−10.0	293	UMHMNP124561202	−8.6	−9.5	−9.9
238	UMHMNP121714747	−9.8	−9.3	−9.9	294	UMHMNP151564022	−9.1	−9.3	−9.9
239	UMHMNP56525736	−8.3	−10.0	−9.9	295	UMHMNP153209122	−8.4	−9.7	−9.9
240	UMHMNP876756568	−8.8	−10.2	−9.9	296	UMHMNP19716268	−9.2	−9.3	−9.9
241	UMHMNP136762997	−8.4	−9.4	−9.9	297	UMHMNP32352651	−9.6	−9.3	−9.9
242	UMHMNP189083781	−9.3	−9.3	−9.9	298	UMHMNP479411455	−9.2	−9.3	−9.9
243	UMHMNP193008263	−9.3	−9.3	−9.9	299	UMHMNP123158907	−9.0	−10.0	−9.9
244	UMHMNP4298004	−9.4	−10.4	−9.9	300	UMHMNP141321133	−8.4	−9.9	−9.9
245	UMHMNP88147197	−9.3	−10.2	−9.9	301	UMHMNP143049154	−10.3	−10.3	−9.9
246	UMHMNP121678873	−9.2	−10.3	−9.9	302	UMHMNP168004851	−9.1	−9.3	−9.9
247	UMHMNP133883224	−9.7	−9.7	−9.9	303	UMHMNP188111708	−9.2	−9.4	−9.9
248	UMHMNP78342388	−8.4	−9.3	−9.9	304	UMHMNP2364230	−9.2	−9.8	−9.9
249	UMHMNP85617750	−9.8	−9.9	−9.9	305	UMHMNP851610907	−9.8	−10.0	−9.9
250	UMHMNP90108637	−10.1	−10.1	−9.9	306	UMHMNP97564948	−9.7	−9.8	−9.9
251	UMHMNP115268434	−8.8	−9.3	−9.9	307	UMHMNP106534445	−9.4	−9.4	−9.9
252	UMHMNP211358655	−8.8	−9.7	−9.9	308	UMHMNP113592817	−10.1	−10.2	−9.9
253	UMHMNP25747448	−9.8	−9.3	−9.9	309	UMHMNP38636509	−8.6	−10.1	−9.9
254	UMHMNP272118066	−8.6	−9.5	−9.9	310	UMHMNP76758208	−9.8	−10.0	−9.9
255	UMHMNP76094569	−9.5	−9.5	−9.9	311	UMHMNP874193610	−9.9	−10.0	−9.9
256	UMHMNP858950444	−8.9	−9.3	−9.9	312	UMHMNP139765336	−9.8	−10.0	−9.9
257	UMHMNP90195418	−9.5	−9.3	−9.9	313	UMHMNP14050625	−9.7	−9.8	−9.9
258	UMHMNP102054455	−9.5	−9.9	−9.9	314	UMHMNP160098931	−8.7	−9.3	−9.9
259	UMHMNP136781958	−9.8	−10.3	−9.9	315	UMHMNP306971120	−10.7	−10.7	−9.9
260	UMHMNP199600390	−8.4	−9.7	−9.9	316	UMHMNP329050228	−9.8	−9.9	−9.9
261	UMHMNP62410962	−10.1	−9.3	−9.9	317	UMHMNP474624	−9.1	−10.1	−9.9
262	UMHMNP83115440	−9.4	−9.5	−9.9	318	UMHMNP52936706	−8.8	−9.6	−9.9
263	UMHMNP125127573	−8.6	−9.3	−9.9	319	UMHMNP55688454	−9.2	−10.2	−9.9
264	UMHMNP57874	−8.2	−9.3	−9.9	320	UMHMNP693790748	−9.7	−9.3	−9.9
265	UMHMNP78370840	−8.8	−9.8	−9.9	321	UMHMNP753457942	−8.3	−9.3	−9.9
266	UMHMNP94806049	−9.5	−9.6	−9.9	322	UMHMNP85733762	−9.7	−9.7	−9.9
267	UMHMNP113597038	−9.0	−9.6	−9.9	323	UMHMNP134887277	−8.7	−9.3	−9.9
268	UMHMNP119147125	−8.9	−9.7	−9.9	324	UMHMNP150931832	−9.5	−9.5	−9.9
269	UMHMNP233607711	−8.8	−9.9	−9.9	325	UMHMNP329050251	−9.0	−9.9	−9.9
270	UMHMNP73792935	−8.4	−9.5	−9.9	326	UMHMNP870535118	−9.1	−9.3	−9.9
271	UMHMNP83454	−9.9	−10.0	−9.9	327	UMHMNP17752168	−9.3	−10.2	−9.9
272	UMHMNP871943985	−9.8	−10.1	−9.9	328	UMHMNP5371540	−10.1	−10.1	−9.9
273	UMHMNP132911456	−8.1	−10.4	−9.9	329	UMHMNP675106226	−8.1	−9.3	−9.9
274	UMHMNP142755155	−9.3	−9.4	−9.9	330	UMHMNP851610861	−9.1	−9.3	−9.9
275	UMHMNP151890845	−8.9	−9.8	−9.9	331	UMHMNP211239417	−8.8	−9.7	−9.9
276	UMHMNP200942183	−8.1	−9.3	−9.9	332	UMHMNP24778516	−9.3	−9.5	−9.9
277	UMHMNP244157980	−8.8	−9.7	−9.9	333	UMHMNP32450252	−9.5	−9.4	−9.9

Table S2. *Continued.*

No.	Compound Name/Code	Docking Score (kcal/mol)			No.	Compound Name/Code	Docking Score (kcal/mol)		
		Fast	Moderate	Expensive			Fast	Moderate	Expensive
334	UMHMNP4736565	−9.2	−9.3	−9.9	390	UMHMNP105969640	−9.7	−9.3	−9.8
335	UMHMNP78285844	−10.1	−10.4	−9.9	391	UMHMNP144686420	−9.8	−9.3	−9.8
336	UMHMNP140389497	−8.1	−9.4	−9.9	392	UMHMNP53296665	−9.7	−9.8	−9.8
337	UMHMNP150627364	−8.3	−9.3	−9.9	393	UMHMNP83115484	−9.3	−9.4	−9.8
338	UMHMNP473030	−8.6	−9.3	−9.9	394	UMHMNP858950557	−9.4	−9.4	−9.8
339	UMHMNP89495476	−9.5	−10.1	−9.9	395	UMHMNP100942721	−9.1	−9.6	−9.8
340	UMHMNP116407217	−10.3	−9.4	−9.9	396	UMHMNP1247649	−9.5	−9.9	−9.8
341	UMHMNP119212281	−8.2	−9.3	−9.9	397	UMHMNP134887266	−9.3	−10.5	−9.8
342	UMHMNP132310908	−10.4	−9.3	−9.9	398	UMHMNP149260742	−8.8	−9.3	−9.8
343	UMHMNP149260800	−8.7	−9.5	−9.9	399	UMHMNP161776767	−8.1	−9.3	−9.8
344	UMHMNP151890834	−9.5	−9.4	−9.9	400	UMHMNP173681522	−8.3	−11.2	−9.8
345	UMHMNP20780410	−9.6	−9.5	−9.9	401	UMHMNP219832378	−8.6	−9.9	−9.8
346	UMHMNP233607722	−8.7	−9.7	−9.9	402	UMHMNP81387839	−9.3	−9.9	−9.8
347	UMHMNP73731245	−9.6	−9.3	−9.9	403	UMHMNP863919179	−9.6	−9.8	−9.8
348	UMHMNP80388507	−9.2	−9.3	−9.9	404	UMHMNP97888158	−10.0	−10.0	−9.8
349	UMHMNP114820290	−8.8	−9.3	−9.9	405	UMHMNP102054466	−8.3	−9.7	−9.8
350	UMHMNP121714747	−9.8	−9.3	−9.9	406	UMHMNP102607761	−10.1	−10.1	−9.8
351	UMHMNP193008274	−8.5	−9.3	−9.9	407	UMHMNP112923407	−8.5	−9.3	−9.8
352	UMHMNP459832678	−9.3	−9.3	−9.9	408	UMHMNP149378563	−9.0	−9.3	−9.8
353	UMHMNP80525491	−9.2	−9.6	−9.9	409	UMHMNP158149543	−10.5	−10.2	−9.8
354	UMHMNP847605769	−9.7	−10.0	−9.9	410	UMHMNP179523387	−8.1	−9.3	−9.8
355	UMHMNP84873143	−8.9	−9.6	−9.9	411	UMHMNP197381605	−10.9	−9.6	−9.8
356	UMHMNP886851269	−8.4	−9.9	−9.9	412	UMHMNP50868503	−8.9	−9.6	−9.8
357	UMHMNP126297390	−8.8	−9.7	−9.8	413	UMHMNP52936693	−8.8	−10.1	−9.8
358	UMHMNP18099246	−8.9	−9.4	−9.8	414	UMHMNP59048872	−9.0	−9.5	−9.8
359	UMHMNP2140467	−8.1	−9.8	−9.8	415	UMHMNP63121062	−8.8	−9.5	−9.8
360	UMHMNP224784489	−8.7	−9.3	−9.8	416	UMHMNP866403716	−9.2	−9.5	−9.8
361	UMHMNP477935969	−8.2	−9.8	−9.8	417	UMHMNP96363063	−10.1	−10.1	−9.8
362	UMHMNP55945749	−9.6	−9.8	−9.8	418	UMHMNP102054488	−9.5	−9.8	−9.8
363	UMHMNP858950466	−9.2	−9.3	−9.8	419	UMHMNP116079539	−8.9	−10.2	−9.8
364	UMHMNP133738484	−8.2	−9.3	−9.8	420	UMHMNP132214234	−8.8	−10.3	−9.8
365	UMHMNP168075147	−9.3	−9.3	−9.8	421	UMHMNP157459240	−9.3	−9.7	−9.8
366	UMHMNP186593862	−10.0	−10.3	−9.8	422	UMHMNP2137168	−9.5	−10.3	−9.8
367	UMHMNP233607697	−8.5	−9.5	−9.8	423	UMHMNP334491257	−8.1	−9.3	−9.8
368	UMHMNP607388874	−8.5	−9.8	−9.8	424	UMHMNP53282681	−9.1	−9.3	−9.8
369	UMHMNP78094005	−8.6	−9.3	−9.8	425	UMHMNP752211908	−9.4	−9.5	−9.8
370	UMHMNP78183296	−8.6	−9.4	−9.8	426	UMHMNP771534362	−10.0	−10.0	−9.8
371	UMHMNP97889560	−8.8	−9.3	−9.8	427	UMHMNP169565779	−8.1	−9.7	−9.8
372	UMHMNP168569151	−8.2	−9.3	−9.8	428	UMHMNP171784104	−8.7	−9.3	−9.8
373	UMHMNP246036862	−10.0	−9.5	−9.8	429	UMHMNP199012867	−9.5	−9.8	−9.8
374	UMHMNP34751252	−8.6	−10.7	−9.8	430	UMHMNP693790737	−9.1	−10.1	−9.8
375	UMHMNP639512199	−8.7	−9.8	−9.8	431	UMHMNP823804837	−8.7	−9.3	−9.8
376	UMHMNP720681630	−10.1	−10.5	−9.8	432	UMHMNP219832356	−8.4	−9.3	−9.7
377	UMHMNP102054477	−8.3	−9.7	−9.8	433	UMHMNP4680379	−8.1	−9.9	−9.7
378	UMHMNP161470253	−8.1	−9.6	−9.8	434	UMHMNP681240608	−9.4	−9.4	−9.7
379	UMHMNP172377503	−9.7	−11.0	−9.8	435	UMHMNP83198270	−10.2	−10.5	−9.7
380	UMHMNP477885384	−9.0	−9.4	−9.8	436	UMHMNP85733739	−9.7	−9.7	−9.7
381	UMHMNP75197385	−8.5	−9.3	−9.8	437	UMHMNP94806050	−8.8	−9.8	−9.7
382	UMHMNP862587186	−8.6	−10.0	−9.8	438	UMHMNP114550753	−9.9	−10.0	−9.7
383	UMHMNP94806027	−9.3	−9.8	−9.8	439	UMHMNP131487019	−8.3	−9.9	−9.7
384	UMHMNP123332876	−8.2	−9.3	−9.8	440	UMHMNP229956472	−8.0	−9.5	−9.7
385	UMHMNP185331946	−9.0	−9.3	−9.8	441	UMHMNP61947942	−9.0	−9.3	−9.7
386	UMHMNP37976888	−10.3	−9.6	−9.8	442	UMHMNP194148997	−8.1	−9.4	−9.7
387	UMHMNP55544359	−8.3	−9.6	−9.8	443	UMHMNP22149695	−8.0	−9.7	−9.7
388	UMHMNP71242494	−9.8	−9.9	−9.8	444	UMHMNP22350498	−9.4	−9.9	−9.7
389	UMHMNP83728818	−9.7	−10.0	−9.8	445	UMHMNP433717385	−9.0	−9.4	−9.7

Table S2. *Continued.*

No.	Compound Name/Code	Docking Score (kcal/mol)			No.	Compound Name/Code	Docking Score (kcal/mol)		
		Fast	Moderate	Expensive			Fast	Moderate	Expensive
446	UMHMNP481174	−8.8	−10.7	−9.7	502	UMHMNP62008042	−10.0	−9.5	−9.7
447	UMHMNP681240631	−9.3	−9.4	−9.7	503	UMHMNP108868957	−9.6	−9.5	−9.6
448	UMHMNP85733784	−9.2	−9.5	−9.7	504	UMHMNP153444872	−9.2	−9.4	−9.6
449	UMHMNP152110082	−8.2	−9.3	−9.7	505	UMHMNP193074496	−9.3	−9.3	−9.6
450	UMHMNP171784091	−8.9	−9.3	−9.7	506	UMHMNP290353683	−9.4	−9.6	−9.6
451	UMHMNP200625269	−8.3	−9.8	−9.7	507	UMHMNP474635	−8.3	−9.3	−9.6
452	UMHMNP33646785	−8.8	−9.5	−9.7	508	UMHMNP61897903	−8.6	−9.4	−9.6
453	UMHMNP478364180	−8.7	−10.2	−9.7	509	UMHMNP67314152	−9.3	−9.3	−9.6
454	UMHMNP64997520	−9.8	−10.3	−9.7	510	UMHMNP81306583	−9.2	−9.4	−9.6
455	UMHMNP75917894	−8.2	−9.3	−9.7	511	UMHMNP109152332	−8.9	−9.6	−9.6
456	UMHMNP96886123	−8.7	−9.7	−9.7	512	UMHMNP121449943	−8.4	−9.5	−9.6
457	UMHMNP160666524	−8.8	−11.1	−9.7	513	UMHMNP122540276	−9.8	−9.8	−9.6
458	UMHMNP85748125	−10.1	−10.1	−9.7	514	UMHMNP145398647	−9.6	−9.3	−9.6
459	UMHMNP92264136	−9.4	−9.5	−9.7	515	UMHMNP159813679	−8.6	−10.5	−9.6
460	UMHMNP114820278	−9.7	−9.7	−9.7	516	UMHMNP169564969	−8.1	−9.7	−9.6
461	UMHMNP123158952	−8.3	−10.2	−9.7	517	UMHMNP54602072	−8.4	−10.3	−9.6
462	UMHMNP131711369	−9.8	−9.5	−9.7	518	UMHMNP76836174	−9.1	−9.5	−9.6
463	UMHMNP148717913	−10.5	−9.9	−9.7	519	UMHMNP78285866	−8.8	−9.4	−9.6
464	UMHMNP151606245	−8.1	−9.3	−9.7	520	UMHMNP866403738	−10.8	−10.9	−9.6
465	UMHMNP223271770	−8.7	−9.3	−9.7	521	UMHMNP148371088	−8.9	−9.5	−9.6
466	UMHMNP244303773	−8.8	−9.7	−9.7	522	UMHMNP160014880	−8.4	−11.2	−9.6
467	UMHMNP287965280	−10.0	−10.1	−9.7	523	UMHMNP55309687	−8.1	−9.3	−9.6
468	UMHMNP33168773	−9.4	−9.7	−9.7	524	UMHMNP76376324	−9.1	−9.3	−9.6
469	UMHMNP473739401	−8.3	−9.3	−9.7	525	UMHMNP88191064	−9.7	−10.3	−9.6
470	UMHMNP69081885	−9.4	−9.4	−9.7	526	UMHMNP107585013	−9.0	−9.3	−9.6
471	UMHMNP134128157	−9.7	−9.7	−9.7	527	UMHMNP114820303	−10.4	−10.4	−9.6
472	UMHMNP149764329	−9.0	−9.3	−9.7	528	UMHMNP172854783	−8.9	−10.6	−9.6
473	UMHMNP157459251	−9.3	−9.7	−9.7	529	UMHMNP243668124	−9.7	−9.7	−9.6
474	UMHMNP244066055	−8.6	−9.7	−9.7	530	UMHMNP78418458	−8.6	−9.4	−9.6
475	UMHMNP63635438	−8.2	−9.3	−9.7	531	UMHMNP97888147	−10.4	−10.4	−9.6
476	UMHMNP710324962	−9.7	−9.7	−9.7	532	UMHMNP115982199	−9.3	−9.3	−9.6
477	UMHMNP158758419	−8.8	−9.3	−9.7	533	UMHMNP121714769	−9.5	−9.3	−9.6
478	UMHMNP2643029	−9.8	−10.0	−9.7	534	UMHMNP178200936	−8.9	−10.8	−9.6
479	UMHMNP481254	−9.0	−9.3	−9.7	535	UMHMNP521040	−10.1	−9.3	−9.6
480	UMHMNP75795890	−9.5	−9.8	−9.7	536	UMHMNP681240620	−8.1	−9.5	−9.6
481	UMHMNP77715867	−9.7	−9.9	−9.7	537	UMHMNP76758184	−8.8	−9.3	−9.6
482	UMHMNP83681816	−8.8	−9.4	−9.7	538	UMHMNP77794811	−10.1	−9.5	−9.6
483	UMHMNP882524916	−9.6	−9.9	−9.7	539	UMHMNP78342402	−9.7	−9.8	−9.6
484	UMHMNP109152398	−9.5	−9.5	−9.7	540	UMHMNP80358005	−8.9	−9.4	−9.6
485	UMHMNP114836872	−8.1	−9.3	−9.7	541	UMHMNP80677765	−9.2	−9.5	−9.6
486	UMHMNP141344107	−9.9	−9.9	−9.7	542	UMHMNP160632280	−9.1	−9.6	−9.6
487	UMHMNP19383870	−8.2	−9.4	−9.7	543	UMHMNP287964867	−10.2	−10.1	−9.6
488	UMHMNP197381570	−9.4	−9.5	−9.7	544	UMHMNP433717534	−9.2	−9.3	−9.6
489	UMHMNP204716041	−8.9	−9.4	−9.7	545	UMHMNP55688501	−9.7	−9.5	−9.6
490	UMHMNP58497293	−10.5	−9.3	−9.7	546	UMHMNP107168570	−9.9	−10.1	−9.6
491	UMHMNP705279716	−9.1	−9.3	−9.7	547	UMHMNP133883177	−10.7	−10.8	−9.6
492	UMHMNP119760848	−10.4	−10.3	−9.7	548	UMHMNP169387740	−8.2	−9.5	−9.6
493	UMHMNP134981785	−8.3	−9.3	−9.7	549	UMHMNP232585158	−8.7	−9.9	−9.6
494	UMHMNP154563798	−8.3	−9.4	−9.7	550	UMHMNP246037605	−8.6	−10.1	−9.6
495	UMHMNP165815827	−8.6	−9.5	−9.7	551	UMHMNP26040006	−8.4	−9.5	−9.6
496	UMHMNP191212369	−8.6	−9.8	−9.7	552	UMHMNP368421356	−8.7	−9.3	−9.6
497	UMHMNP221300001	−8.4	−9.5	−9.7	553	UMHMNP452934951	−8.0	−10.4	−9.6
498	UMHMNP34212936	−8.6	−9.5	−9.7	554	UMHMNP64907240	−9.5	−9.4	−9.6
499	UMHMNP37772035	−9.7	−10.0	−9.7	555	UMHMNP80357999	−8.4	−9.4	−9.6
500	UMHMNP508193315	−9.8	−9.3	−9.7	556	UMHMNP156472898	−8.9	−9.5	−9.6
501	UMHMNP567726	−9.2	−9.4	−9.7	557	UMHMNP184885072	−9.5	−9.5	−9.6

Table S2. *Continued.*

No.	Compound Name/Code	Docking Score (kcal/mol)			No.	Compound Name/Code	Docking Score (kcal/mol)		
		Fast	Moderate	Expensive			Fast	Moderate	Expensive
558	UMHMNP23869161	−10.0	−9.4	−9.6	614	UMHMNP119539752	−8.2	−9.3	−9.6
559	UMHMNP242467534	−9.7	−9.7	−9.6	615	UMHMNP132214245	−9.0	−9.9	−9.6
560	UMHMNP81262966	−9.3	−10.1	−9.6	616	UMHMNP221169751	−8.3	−9.5	−9.6
561	UMHMNP82507264	−9.7	−9.7	−9.6	617	UMHMNP287964856	−10.4	−10.5	−9.6
562	UMHMNP86105659	−9.2	−9.5	−9.6	618	UMHMNP52645097	−8.5	−9.9	−9.6
563	UMHMNP133530253	−8.3	−9.5	−9.6	619	UMHMNP57830	−9.2	−9.5	−9.6
564	UMHMNP15146619	−8.1	−10.4	−9.6	620	UMHMNP745075267	−9.4	−9.6	−9.6
565	UMHMNP152833608	−9.4	−9.3	−9.6	621	UMHMNP77983267	−9.7	−9.8	−9.6
566	UMHMNP170894367	−9.5	−9.8	−9.6	622	UMHMNP82637056	−9.7	−9.7	−9.6
567	UMHMNP96603020	−9.1	−9.3	−9.6	623	UMHMNP96313950	−10.1	−10.3	−9.6
568	UMHMNP126596030	−8.1	−10.0	−9.6	624	UMHMNP180603672	−9.4	−9.6	−9.6
569	UMHMNP158402610	−8.6	−10.3	−9.6	625	UMHMNP181306741	−8.4	−9.3	−9.6
570	UMHMNP211555021	−9.3	−9.8	−9.6	626	UMHMNP185331957	−9.3	−9.4	−9.6
571	UMHMNP306997422	−8.6	−10.4	−9.6	627	UMHMNP196500773	−8.4	−9.4	−9.6
572	UMHMNP3963379	−9.4	−9.9	−9.6	628	UMHMNP20465901	−8.7	−10.1	−9.6
573	UMHMNP516370	−8.8	−9.8	−9.6	629	UMHMNP41083970	−8.5	−9.3	−9.6
574	UMHMNP120154963	−8.4	−10.9	−9.6	630	UMHMNP517900582	−9.2	−9.6	−9.6
575	UMHMNP184584405	−9.4	−9.3	−9.6	631	UMHMNP75266250	−9.4	−9.4	−9.6
576	UMHMNP313042	−9.6	−9.6	−9.6	632	UMHMNP82507253	−8.8	−9.3	−9.6
577	UMHMNP393828336	−8.8	−9.3	−9.6	633	UMHMNP86690144	−10.2	−10.5	−9.6
578	UMHMNP4936101	−8.2	−9.6	−9.6	634	UMHMNP114820267	−9.7	−9.7	−9.6
579	UMHMNP70165456	−10.7	−9.3	−9.6	635	UMHMNP126596029	−9.6	−9.9	−9.6
580	UMHMNP85748136	−10.0	−10.0	−9.6	636	UMHMNP133562524	−8.6	−9.3	−9.6
581	UMHMNP94444254	−8.4	−9.3	−9.6	637	UMHMNP144398550	−8.8	−9.7	−9.6
582	UMHMNP126622615	−8.4	−10.1	−9.6	638	UMHMNP2061645	−8.1	−9.3	−9.6
583	UMHMNP129829909	−8.5	−9.8	−9.6	639	UMHMNP337379198	−9.9	−9.3	−9.6
584	UMHMNP14729294	−8.9	−9.3	−9.6	640	UMHMNP80525479	−8.5	−9.5	−9.6
585	UMHMNP157521090	−9.4	−9.8	−9.6	641	UMHMNP83511846	−9.6	−10.0	−9.6
586	UMHMNP21317817	−9.2	−10.1	−9.6	642	UMHMNP882400853	−9.5	−9.5	−9.6
587	UMHMNP226716945	−9.1	−10.1	−9.6	643	UMHMNP100994556	−9.0	−9.3	−9.6
588	UMHMNP61586003	−9.1	−10.5	−9.6	644	UMHMNP124596607	−8.5	−11.2	−9.6
589	UMHMNP156953898	−9.3	−11.2	−9.6	645	UMHMNP142780436	−8.3	−9.3	−9.6
590	UMHMNP160014879	−8.3	−9.7	−9.6	646	UMHMNP157758742	−8.1	−10.6	−9.6
591	UMHMNP171440247	−8.9	−10.6	−9.6	647	UMHMNP195259826	−9.6	−10.5	−9.6
592	UMHMNP178115907	−8.8	−9.4	−9.6	648	UMHMNP51630581	−9.2	−9.3	−9.6
593	UMHMNP37299084	−9.6	−9.5	−9.6	649	UMHMNP58701705	−9.5	−10.1	−9.6
594	UMHMNP452082596	−8.4	−10.6	−9.6	650	UMHMNP76343947	−9.8	−9.9	−9.6
595	UMHMNP97614625	−8.8	−9.3	−9.6	651	UMHMNP771534384	−9.6	−9.6	−9.6
596	UMHMNP107168581	−8.2	−9.7	−9.6	652	UMHMNP81387840	−9.3	−9.4	−9.6
597	UMHMNP134887255	−8.3	−9.4	−9.6	653	UMHMNP87532283	−9.2	−9.3	−9.6
598	UMHMNP154496874	−10.9	−10.5	−9.6	654	UMHMNP141266060	−9.4	−9.4	−9.6
599	UMHMNP172173987	−8.5	−9.3	−9.6	655	UMHMNP214483235	−8.1	−9.6	−9.6
600	UMHMNP298196744	−8.1	−9.3	−9.6	656	UMHMNP264254866	−9.7	−9.8	−9.6
601	UMHMNP72542495	−9.3	−9.4	−9.6	657	UMHMNP50909858	−9.6	−10.4	−9.6
602	UMHMNP89411314	−8.8	−9.3	−9.6	658	UMHMNP61973408	−9.5	−10.0	−9.6
603	UMHMNP145176858	−8.5	−9.3	−9.6	659	UMHMNP651489	−8.2	−9.9	−9.6
604	UMHMNP26808275	−9.7	−9.8	−9.6	660	UMHMNP161536249	−8.4	−9.4	−9.6
605	UMHMNP321847103	−8.3	−9.3	−9.6	661	UMHMNP181034935	−9.9	−10.4	−9.6
606	UMHMNP75886123	−9.7	−9.8	−9.6	662	UMHMNP39832310	−9.3	−9.3	−9.6
607	UMHMNP76898481	−9.6	−9.7	−9.6	663	UMHMNP61737971	−9.1	−9.6	−9.6
608	UMHMNP864514590	−9.6	−9.8	−9.6	664	UMHMNP674819466	−8.8	−9.5	−9.6
609	UMHMNP86748303	−9.9	−10.3	−9.6	665	UMHMNP72534135	−8.4	−9.7	−9.6
610	UMHMNP125282135	−8.4	−9.3	−9.6	666	UMHMNP81120743	−8.7	−9.8	−9.6
611	UMHMNP147391819	−8.2	−9.3	−9.6	667	UMHMNP850145129	−9.7	−9.9	−9.6
612	UMHMNP5813650	−8.3	−9.3	−9.6	668	UMHMNP94806038	−9.3	−9.8	−9.6
613	UMHMNP81275823	−10.4	−10.4	−9.6	669	UMHMNP103005205	−9.6	−9.7	−9.6

Table S2. *Continued.*

No.	Compound Name/Code	Docking Score (kcal/mol)			No.	Compound Name/Code	Docking Score (kcal/mol)		
		Fast	Moderate	Expensive			Fast	Moderate	Expensive
670	UMHMNP109152343	−10.0	−10.5	−9.6	726	UMHMNP87307286	−8.9	−9.3	−9.6
671	UMHMNP145038579	−9.5	−9.3	−9.6	727	UMHMNP112781234	−9.3	−9.5	−9.6
672	UMHMNP179936528	−8.6	−9.4	−9.6	728	UMHMNP121825443	−8.3	−10.2	−9.6
673	UMHMNP26531715	−8.6	−10.3	−9.6	729	UMHMNP163318783	−8.3	−11.2	−9.6
674	UMHMNP798557989	−9.4	−10.0	−9.6	730	UMHMNP17991672	−9.2	−9.5	−9.6
675	UMHMNP86105682	−8.9	−10.2	−9.6	731	UMHMNP221160845	−8.6	−9.7	−9.6
676	UMHMNP114820289	−9.1	−9.3	−9.6	732	UMHMNP38636496	−9.8	−9.5	−9.6
677	UMHMNP143049121	−8.4	−9.9	−9.6	733	UMHMNP614748628	−9.1	−9.3	−9.6
678	UMHMNP14958668	−8.1	−9.3	−9.6	734	UMHMNP862522	−9.5	−9.5	−9.6
679	UMHMNP175673582	−8.5	−9.9	−9.6	735	UMHMNP153415351	−9.4	−9.3	−9.6
680	UMHMNP199600345	−8.7	−9.5	−9.6	736	UMHMNP153698909	−8.4	−10.0	−9.6
681	UMHMNP209167259	−9.4	−9.6	−9.6	737	UMHMNP174796782	−8.4	−9.3	−9.6
682	UMHMNP570898	−9.0	−10.3	−9.6	738	UMHMNP175861800	−8.4	−9.4	−9.6
683	UMHMNP75373625	−8.5	−9.3	−9.6	739	UMHMNP183381068	−8.5	−9.4	−9.6
684	UMHMNP858950386	−9.8	−9.8	−9.6	740	UMHMNP199600378	−8.4	−9.7	−9.6
685	UMHMNP861676575	−9.3	−9.3	−9.6	741	UMHMNP250147194	−9.2	−10.0	−9.6
686	UMHMNP862288902	−9.3	−9.7	−9.6	742	UMHMNP306967760	−9.7	−9.9	−9.6
687	UMHMNP87697992	−8.1	−9.4	−9.6	743	UMHMNP395070889	−8.7	−9.5	−9.6
688	UMHMNP108675634	−8.9	−9.8	−9.6	744	UMHMNP425368598	−8.4	−9.8	−9.6
689	UMHMNP114820256	−8.2	−9.4	−9.6	745	UMHMNP81657398	−9.9	−9.3	−9.6
690	UMHMNP132922873	−8.6	−10.0	−9.6	746	UMHMNP81657796	−9.3	−9.3	−9.6
691	UMHMNP141754576	−9.5	−9.5	−9.6	747	UMHMNP85505664	−8.4	−9.7	−9.6
692	UMHMNP189457269	−9.5	−9.5	−9.6	748	UMHMNP93426916	−9.9	−10.1	−9.6
693	UMHMNP196500808	−8.7	−9.3	−9.6	749	UMHMNP109979171	−9.4	−9.4	−9.6
694	UMHMNP34427639	−10.6	−10.7	−9.6	750	UMHMNP156352657	−8.9	−10.3	−9.6
695	UMHMNP63109171	−9.5	−11.0	−9.6	751	UMHMNP161776756	−8.5	−9.3	−9.6
696	UMHMNP71932064	−10.7	−10.7	−9.6	752	UMHMNP188968447	−8.4	−9.6	−9.6
697	UMHMNP83487	−9.3	−9.8	−9.6	753	UMHMNP448264651	−8.2	−9.7	−9.6
698	UMHMNP103190143	−9.2	−9.4	−9.6	754	UMHMNP636599901	−8.1	−9.3	−9.6
699	UMHMNP140384709	−9.0	−9.3	−9.6	755	UMHMNP143016868	−9.9	−10.7	−9.6
700	UMHMNP175669100	−8.1	−11.2	−9.6	756	UMHMNP149064346	−8.4	−9.9	−9.6
701	UMHMNP53755092	−9.1	−9.3	−9.6	757	UMHMNP14908168	−9.3	−9.3	−9.6
702	UMHMNP694436618	−9.8	−10.2	−9.6	758	UMHMNP155850887	−8.8	−10.7	−9.6
703	UMHMNP74799556	−8.3	−9.3	−9.6	759	UMHMNP179730360	−8.4	−9.4	−9.6
704	UMHMNP78285855	−10.4	−10.5	−9.6	760	UMHMNP183593706	−10.9	−11.0	−9.6
705	UMHMNP78342377	−8.8	−9.8	−9.6	761	UMHMNP268735575	−10.0	−10.0	−9.6
706	UMHMNP90352208	−8.5	−9.3	−9.6	762	UMHMNP57885	−8.5	−9.3	−9.6
707	UMHMNP134985072	−8.3	−9.3	−9.6	763	UMHMNP74055428	−9.4	−9.8	−9.6
708	UMHMNP16826418	−8.5	−9.3	−9.6	764	UMHMNP85198216	−9.7	−10.6	−9.6
709	UMHMNP172854772	−8.6	−9.3	−9.6	765	UMHMNP94354980	−10.0	−10.1	−9.6
710	UMHMNP194148975	−9.3	−9.4	−9.6	766	UMHMNP107551746	−10.0	−9.3	−9.6
711	UMHMNP100667752	−9.4	−9.3	−9.6	767	UMHMNP117857724	−8.7	−9.7	−9.6
712	UMHMNP143503224	−8.4	−9.4	−9.6	768	UMHMNP1242019	−9.6	−9.5	−9.6
713	UMHMNP173681533	−8.8	−9.3	−9.6	769	UMHMNP13963138	−9.5	−9.3	−9.6
714	UMHMNP71678030	−9.0	−9.3	−9.6	770	UMHMNP152845755	−8.1	−10.2	−9.6
715	UMHMNP1174921	−9.6	−9.3	−9.6	771	UMHMNP2465114	−9.4	−10.5	−9.6
716	UMHMNP128855072	−9.0	−9.4	−9.6	772	UMHMNP50611864	−9.0	−9.3	−9.6
717	UMHMNP140866202	−9.8	−9.9	−9.6	773	UMHMNP53755105	−8.7	−11.2	−9.6
718	UMHMNP306997397	−9.2	−9.3	−9.6	774	UMHMNP71885206	−10.0	−9.4	−9.6
719	UMHMNP31893353	−8.7	−10.0	−9.6	775	UMHMNP735278270	−8.2	−9.3	−9.6
720	UMHMNP65773980	−9.3	−9.4	−9.6	776	UMHMNP93426927	−9.2	−9.4	−9.6
721	UMHMNP675106204	−9.1	−9.9	−9.6	777	UMHMNP97719783	−9.3	−9.3	−9.6
722	UMHMNP70214925	−10.1	−10.2	−9.6	778	UMHMNP106534434	−9.2	−10.4	−9.6
723	UMHMNP75479117	−9.2	−9.3	−9.6	779	UMHMNP132750482	−8.7	−9.5	−9.6
724	UMHMNP80981631	−8.3	−9.3	−9.6	780	UMHMNP37976899	−8.8	−10.5	−9.6
725	UMHMNP86748278	−9.8	−9.9	−9.6	781	UMHMNP452082609	−9.3	−9.6	−9.6

Table S2. *Continued.*

No.	Compound Name/Code	Docking Score (kcal/mol)			No.	Compound Name/Code	Docking Score (kcal/mol)		
		Fast	Moderate	Expensive			Fast	Moderate	Expensive
782	UMHMNP67463790	−9.6	−9.4	−9.6	838	UMHMNP145038580	−9.0	−9.3	−9.6
783	UMHMNP749867230	−9.8	−10.2	−9.6	839	UMHMNP147641723	−9.6	−9.4	−9.6
784	UMHMNP124596629	−8.8	−10.1	−9.6	840	UMHMNP162290417	−8.3	−9.3	−9.6
785	UMHMNP141859974	−9.7	−9.6	−9.6	841	UMHMNP24041665	−9.0	−9.4	−9.6
786	UMHMNP14231335	−9.4	−10.0	−9.6	842	UMHMNP40772121	−8.5	−9.6	−9.6
787	UMHMNP191789783	−9.0	−10.0	−9.6	843	UMHMNP494862714	−8.7	−9.4	−9.6
788	UMHMNP133883199	−9.1	−9.3	−9.6	844	UMHMNP549520743	−9.7	−9.3	−9.6
789	UMHMNP134515530	−9.1	−9.7	−9.6	845	UMHMNP64180718	−8.3	−10.7	−9.6
790	UMHMNP214483199	−8.9	−9.3	−9.6	846	UMHMNP876620245	−8.5	−9.7	−9.6
791	UMHMNP315209086	−8.8	−9.7	−9.6	847	UMHMNP128802175	−9.3	−9.9	−9.6
792	UMHMNP41083903	−10.4	−9.3	−9.6	848	UMHMNP129620231	−9.0	−10.5	−9.6
793	UMHMNP452929645	−8.7	−10.0	−9.6	849	UMHMNP130246989	−8.5	−9.8	−9.6
794	UMHMNP69511202	−8.3	−9.5	−9.6	850	UMHMNP133738473	−8.2	−9.5	−9.6
795	UMHMNP69672680	−9.0	−9.5	−9.6	851	UMHMNP186803327	−9.0	−10.7	−9.6
796	UMHMNP804531451	−9.1	−9.4	−9.6	852	UMHMNP474577	−8.7	−9.5	−9.6
797	UMHMNP82154210	−9.3	−9.3	−9.6	853	UMHMNP517900504	−8.2	−9.3	−9.6
798	UMHMNP91297073	−9.2	−9.3	−9.6	854	UMHMNP782491785	−8.3	−9.6	−9.6
799	UMHMNP158734248	−9.3	−9.3	−9.6	855	UMHMNP85733773	−10.1	−10.2	−9.6
800	UMHMNP175702360	−9.7	−9.4	−9.6	856	UMHMNP144335148	−8.9	−9.6	−9.6
801	UMHMNP178064410	−8.2	−9.8	−9.6	857	UMHMNP145212391	−9.8	−10.4	−9.6
802	UMHMNP20817929	−8.3	−9.9	−9.6	858	UMHMNP224577382	−8.1	−9.3	−9.6
803	UMHMNP54369141	−8.3	−9.3	−9.6	859	UMHMNP264618215	−9.9	−9.9	−9.6
804	UMHMNP54602141	−9.1	−9.5	−9.6	860	UMHMNP495380477	−8.8	−10.8	−9.6
805	UMHMNP984849	−8.5	−9.9	−9.6	861	UMHMNP54278896	−8.1	−10.1	−9.6
806	UMHMNP116169290	−9.3	−9.3	−9.6	862	UMHMNP57103216	−8.9	−10.5	−9.6
807	UMHMNP122540323	−8.9	−10.1	−9.6	863	UMHMNP690253771	−8.6	−9.3	−9.6
808	UMHMNP129620253	−8.3	−9.4	−9.6	864	UMHMNP73700295	−9.8	−9.8	−9.6
809	UMHMNP175992980	−8.3	−9.3	−9.6	865	UMHMNP75246763	−9.4	−9.4	−9.6
810	UMHMNP2034722	−9.6	−9.6	−9.6	866	UMHMNP871483351	−8.4	−9.3	−9.6
811	UMHMNP306967759	−10.0	−9.3	−9.6	867	UMHMNP104720152	−9.7	−9.4	−9.6
812	UMHMNP32450263	−8.1	−9.3	−9.6	868	UMHMNP106199819	−9.7	−9.7	−9.6
813	UMHMNP54448725	−8.2	−9.6	−9.6	869	UMHMNP133883202	−9.1	−9.7	−9.6
814	UMHMNP73538575	−9.3	−9.3	−9.6	870	UMHMNP1491776	−8.3	−9.3	−9.6
815	UMHMNP78518748	−8.0	−9.6	−9.6	871	UMHMNP14956242	−10.7	−9.3	−9.6
816	UMHMNP122759675	−9.2	−9.6	−9.6	872	UMHMNP189514485	−8.1	−9.3	−9.6
817	UMHMNP127709459	−8.5	−10.3	−9.6	873	UMHMNP237755167	−8.2	−9.3	−9.6
818	UMHMNP132410356	−8.3	−9.8	−9.6	874	UMHMNP487016966	−8.8	−10.0	−9.6
819	UMHMNP153585649	−8.2	−9.4	−9.6	875	UMHMNP70329258	−8.1	−9.3	−9.6
820	UMHMNP157403333	−9.7	−11.2	−9.6	876	UMHMNP123086817	−8.7	−9.3	−9.6
821	UMHMNP17181883	−10.7	−9.9	−9.6	877	UMHMNP139765303	−8.3	−9.3	−9.6
822	UMHMNP17974786	−9.3	−9.8	−9.6	878	UMHMNP16250616	−11.4	−9.3	−9.6
823	UMHMNP211358713	−8.2	−9.8	−9.6	879	UMHMNP189818493	−8.6	−9.6	−9.6
824	UMHMNP39832309	−8.2	−10.3	−9.6	880	UMHMNP263764041	−8.4	−9.6	−9.6
825	UMHMNP566916	−8.7	−9.9	−9.6	881	UMHMNP333970131	−8.4	−9.4	−9.6
826	UMHMNP87859981	−9.3	−9.9	−9.6	882	UMHMNP38788817	−9.2	−9.3	−9.6
827	UMHMNP95062296	−8.3	−9.9	−9.6	883	UMHMNP39025279	−8.7	−9.9	−9.6
828	UMHMNP139765358	−8.8	−9.8	−9.6	884	UMHMNP446862697	−8.6	−9.4	−9.6
829	UMHMNP1452295	−9.5	−10.0	−9.6	885	UMHMNP132938126	−8.7	−9.3	−9.6
830	UMHMNP148270160	−9.5	−10.3	−9.6	886	UMHMNP147395093	−10.5	−9.3	−9.6
831	UMHMNP151345061	−10.1	−10.2	−9.6	887	UMHMNP478364226	−8.7	−10.2	−9.6
832	UMHMNP471534	−8.1	−9.3	−9.6	888	UMHMNP69297490	−8.5	−9.3	−9.6
833	UMHMNP516858	−8.9	−10.3	−9.6	889	UMHMNP71031588	−9.7	−9.7	−9.6
834	UMHMNP749216464	−8.7	−9.8	−9.6	890	UMHMNP771534373	−9.4	−9.5	−9.6
835	UMHMNP78355289	−9.6	−10.6	−9.6	891	UMHMNP80525480	−9.0	−9.4	−9.6
836	UMHMNP874221893	−9.1	−9.3	−9.6	892	UMHMNP102054524	−9.3	−9.3	−9.6
837	UMHMNP137570435	−9.8	−9.8	−9.6	893	UMHMNP141859985	−9.7	−9.9	−9.6

Table S2. *Continued.*

No.	Compound Name/Code	Docking Score (kcal/mol)			No.	Compound Name/Code	Docking Score (kcal/mol)		
		Fast	Moderate	Expensive			Fast	Moderate	Expensive
894	UMHMNP149260731	−9.5	−9.5	−9.6	950	UMHMNP852872936	−10.0	−9.3	−9.6
895	UMHMNP156472887	−8.0	−10.1	−9.6	951	UMHMNP116972920	−8.5	−9.8	−9.6
896	UMHMNP158822765	−10.3	−9.3	−9.6	952	UMHMNP139765290	−9.9	−9.3	−9.6
897	UMHMNP175413215	−8.3	−10.1	−9.6	953	UMHMNP143007287	−8.3	−9.9	−9.6
898	UMHMNP209408764	−9.6	−9.4	−9.6	954	UMHMNP178179992	−8.4	−9.4	−9.6
899	UMHMNP22850119	−9.1	−9.3	−9.6	955	UMHMNP2259907	−8.6	−9.9	−9.6
900	UMHMNP24041676	−10.0	−9.3	−9.6	956	UMHMNP260062882	−10.0	−9.3	−9.6
901	UMHMNP24662951	−9.3	−10.2	−9.6	957	UMHMNP39533738	−8.2	−9.5	−9.6
902	UMHMNP31220447	−9.7	−10.0	−9.6	958	UMHMNP479067624	−8.7	−9.3	−9.6
903	UMHMNP461441429	−8.2	−9.3	−9.6	959	UMHMNP58560375	−8.1	−9.6	−9.6
904	UMHMNP73723383	−10.2	−10.2	−9.6	960	UMHMNP744253483	−9.6	−9.8	−9.6
905	UMHMNP848132376	−9.8	−9.9	−9.6	961	UMHMNP1159279	−9.3	−9.3	−9.6
906	UMHMNP862200473	−9.0	−9.8	−9.6	962	UMHMNP132194322	−8.3	−11.2	−9.6
907	UMHMNP117675135	−9.0	−10.0	−9.6	963	UMHMNP157622576	−8.7	−9.9	−9.6
908	UMHMNP151890823	−9.1	−9.6	−9.6	964	UMHMNP178494821	−8.5	−9.5	−9.6
909	UMHMNP174063811	−9.1	−9.3	−9.6	965	UMHMNP225662099	−8.3	−10.3	−9.6
910	UMHMNP209408720	−8.8	−9.6	−9.6	966	UMHMNP85443374	−8.4	−9.7	−9.6
911	UMHMNP2243096	−8.9	−9.4	−9.6	967	UMHMNP105369899	−8.7	−9.3	−9.6
912	UMHMNP38819442	−8.5	−9.3	−9.6	968	UMHMNP115193152	−8.8	−9.3	−9.6
913	UMHMNP4598678	−9.1	−9.8	−9.6	969	UMHMNP147170089	−10.3	−10.4	−9.6
914	UMHMNP50335041	−9.2	−9.5	−9.6	970	UMHMNP169217452	−8.5	−9.9	−9.6
915	UMHMNP64687850	−8.5	−10.3	−9.6	971	UMHMNP334491279	−8.1	−9.3	−9.6
916	UMHMNP81387851	−9.9	−9.4	−9.6	972	UMHMNP395070903	−9.4	−9.3	−9.6
917	UMHMNP96253600	−9.6	−9.6	−9.6	973	UMHMNP495380488	−8.6	−9.3	−9.6
918	UMHMNP135729452	−9.4	−9.6	−9.6	974	UMHMNP51529125	−8.1	−9.6	−9.6
919	UMHMNP141854695	−9.6	−9.3	−9.6	975	UMHMNP724462480	−9.4	−9.6	−9.6
920	UMHMNP55081395	−9.1	−9.5	−9.6	976	UMHMNP85748147	−9.8	−9.8	−9.6
921	UMHMNP601570	−8.8	−9.4	−9.6	977	UMHMNP865308607	−9.5	−9.5	−9.6
922	UMHMNP6035627	−8.9	−9.8	−9.6	978	UMHMNP96736311	−10.3	−10.3	−9.6
923	UMHMNP72154337	−9.1	−9.3	−9.6	979	UMHMNP102054499	−8.9	−9.5	−9.6
924	UMHMNP83465	−9.2	−10.1	−9.6	980	UMHMNP12626185	−8.9	−10.7	−9.6
925	UMHMNP85337151	−9.2	−9.3	−9.6	981	UMHMNP143049132	−9.7	−9.4	−9.6
926	UMHMNP142780367	−8.9	−9.3	−9.6	982	UMHMNP82660615	−9.7	−9.7	−9.6
927	UMHMNP158786755	−9.5	−10.4	−9.6	983	UMHMNP118984466	−8.8	−9.5	−9.6
928	UMHMNP163136058	−8.1	−9.5	−9.6	984	UMHMNP144436066	−8.8	−9.7	−9.6
929	UMHMNP199600367	−9.1	−10.3	−9.6	985	UMHMNP185331935	−8.9	−10.5	−9.6
930	UMHMNP326794176	−8.3	−9.3	−9.6	986	UMHMNP286433805	−10.6	−10.7	−9.6
931	UMHMNP3404226	−9.6	−9.9	−9.6	987	UMHMNP516790	−8.6	−9.8	−9.6
932	UMHMNP517900515	−9.8	−9.9	−9.6	988	UMHMNP58514322	−8.1	−10.4	−9.6
933	UMHMNP517900559	−8.9	−9.5	−9.6	989	UMHMNP849752870	−8.4	−9.3	−9.6
934	UMHMNP518069	−9.2	−9.6	−9.6	990	UMHMNP96853155	−8.8	−9.5	−9.6
935	UMHMNP65228140	−9.5	−9.3	−9.6	991	UMHMNP107551757	−10	−11.0	−9.6
936	UMHMNP745075256	−9.1	−9.3	−9.6	992	UMHMNP1106134	−8.8	−9.8	−9.6
937	UMHMNP87164338	−9.5	−9.5	−9.6	993	UMHMNP116302284	−9.2	−9.9	−9.6
938	UMHMNP876756557	−9.4	−9.4	−9.6	994	UMHMNP133401113	−8.8	−9.3	−9.6
939	UMHMNP126394749	−8.8	−9.7	−9.6	995	UMHMNP25819798	−9.7	−9.8	−9.6
940	UMHMNP133883213	−10.9	−10.3	−9.6	996	UMHMNP298196733	−9.2	−9.3	−9.6
941	UMHMNP142677121	−8.6	−9.3	−9.6	997	UMHMNP313503376	−8.1	−10.1	−9.6
942	UMHMNP14599485	−8.9	−9.7	−9.6	998	UMHMNP33886747	−8.5	−10.0	−9.6
943	UMHMNP15313696	−9.0	−9.7	−9.6	999	UMHMNP90195441	−9.1	−9.3	−9.6
944	UMHMNP161776734	−8.9	−9.8	−9.6	1000	UMHMNP95062310	−8.6	−9.9	−9.6
945	UMHMNP174630116	−8.5	−10.1	−9.6	1001	UMHMNP122540312	−9.5	−9.3	−9.6
946	UMHMNP52043013	−9.7	−9.8	−9.6	1002	UMHMNP149355760	−9.4	−9.4	−9.6
947	UMHMNP55051780	−9.8	−9.3	−9.6	1003	UMHMNP165606699	−8.4	−9.3	−9.6
948	UMHMNP68690926	−10.3	−10.5	−9.6	1004	UMHMNP16910320	−9.0	−9.5	−9.6
949	UMHMNP74799590	−10.1	−10.5	−9.6	1005	UMHMNP184430011	−10.3	−10.2	−9.6

Table S2. *Continued.*

No.	Compound Name/Code	Docking Score (kcal/mol)			No.	Compound Name/Code	Docking Score (kcal/mol)		
		Fast	Moderate	Expensive			Fast	Moderate	Expensive
1006	UMHMNP198274258	−8.3	−10.0	−9.6	1050	UMHMNP80388461	−9.0	−9.4	−9.6
1007	UMHMNP238758424	−8.1	−10.0	−9.6	1051	UMHMNP141754587	−8.5	−9.3	−9.6
1008	UMHMNP516552	−8.5	−9.3	−9.6	1052	UMHMNP10525221	−9.7	−9.8	−9.6
1009	UMHMNP69672704	−9.7	−10.0	−9.6	1053	UMHMNP144436077	−8.2	−9.6	−9.6
1010	UMHMNP70214992	−10.3	−10.3	−9.6	1054	UMHMNP146506414	−10.2	−10.3	−9.6
1011	UMHMNP71801495	−9.3	−9.3	−9.6	1055	UMHMNP162830253	−8.8	−9.5	−9.6
1012	UMHMNP77521	−8.6	−9.4	−9.6	1056	UMHMNP166038284	−10.4	−9.4	−9.6
1013	UMHMNP85337128	−9.9	−10.0	−9.6	1057	UMHMNP815586493	−9.8	−9.8	−9.6
1014	UMHMNP117694969	−9.4	−9.6	−9.6	1058	UMHMNP217449199	−8.4	−9.3	−9.6
1015	UMHMNP145403254	−8.9	−9.7	−9.6	1059	UMHMNP85733808	−9.2	−9.3	−9.6
1016	UMHMNP175861855	−10.9	−10.2	−9.6	1060	UMHMNP149260753	−10	−9.4	−9.6
1017	UMHMNP250141641	−8.1	−9.3	−9.6	1061	UMHMNP263764018	−9.5	−9.7	−9.6
1018	UMHMNP3090811	−8.2	−10.1	−9.6	1062	UMHMNP81474615	−9.4	−9.4	−9.6
1019	UMHMNP62861158	−8.4	−9.5	−9.6	1063	UMHMNP81575066	−9.4	−9.5	−9.6
1020	UMHMNP676271855	−9.0	−9.3	−9.6	1064	UMHMNP186593839	−9.2	−9.3	−9.6
1021	UMHMNP85066780	−10.0	−9.9	−9.6	1065	UMHMNP160014868	−8.3	−9.7	−9.6
1022	UMHMNP85733717	−8.5	−9.3	−9.6	1066	UMHMNP62660202	−8.4	−10.1	−9.6
1023	UMHMNP129602193	−8.1	−9.3	−9.6	1067	UMHMNP333723981	−9.4	−9.7	−9.6
1024	UMHMNP148471856	−9.2	−9.6	−9.6	1068	UMHMNP80981664	−9.4	−9.5	−9.6
1025	UMHMNP175861822	−8.3	−9.8	−9.6	1069	UMHMNP446862722	−8.2	−9.7	−9.6
1026	UMHMNP17974775	−9.7	−9.8	−9.6	1070	UMHMNP147641712	−8.4	−9.3	−9.6
1027	UMHMNP1981904	−9.5	−11.2	−9.6	1071	UMHMNP117021064	−8.6	−9.4	−9.6
1028	UMHMNP208708230	−8.9	−10.2	−9.6	1072	UMHMNP283605458	−9.0	−9.5	−9.6
1029	UMHMNP475111785	−8.6	−9.3	−9.6	1073	UMHMNP866025629	−10.6	−10.7	−9.6
1030	UMHMNP56121427	−9.8	−9.9	−9.6	1074	UMHMNP216770295	−9.1	−9.3	−9.6
1031	UMHMNP63015907	−8.2	−9.3	−9.6	1075	UMHMNP89837978	−9.1	−9.6	−9.6
1032	UMHMNP64285854	−8.3	−9.3	−9.6	1076	UMHMNP681226415	−9.5	−9.8	−9.6
1033	UMHMNP780755375	−9.7	−9.8	−9.6	1077	UMHMNP124843681	−8.7	−9.6	−9.6
1034	UMHMNP863684417	−8.1	−9.8	−9.6	1078	UMHMNP103065209	−9.6	−9.7	−9.6
1035	UMHMNP160098920	−10.6	−11.1	−9.6	1079	UMHMNP153209111	−9.0	−9.7	−9.6
1036	UMHMNP175861877	−9.6	−9.6	−9.6	1080	UMHMNP97190313	−8.7	−9.7	−9.6
1037	UMHMNP815586471	−8.5	−9.5	−9.6	1081	UMHMNP425368623	−9.3	−9.9	−9.6
1038	UMHMNP88191144	−10.8	−10.9	−9.6	1082	UMHMNP882693461	−8.9	−10.0	−9.6
1039	UMHMNP116079540	−9.1	−9.6	−9.6	1083	UMHMNP79580282	−8.1	−9.4	−9.6
1040	UMHMNP131727010	−10.9	−9.9	−9.6	1084	UMHMNP20512316	−8.1	−9.3	−9.6
1041	UMHMNP134985083	−9.3	−9.4	−9.6	1085	UMHMNP174286227	−8.5	−10.6	−9.6
1042	UMHMNP175861797	−8.3	−9.4	−9.6	1086	UMHMNP71947643	−9.5	−9.5	−9.6
1043	UMHMNP200497047	−8.2	−9.3	−9.6	1087	UMHMNP65717886	−8.7	−9.3	−9.6
1044	UMHMNP209169584	−8.1	−9.6	−9.6	1088	UMHMNP168482346	−8.4	−10.6	−9.6
1045	UMHMNP36011195	−9.4	−9.3	−9.6	1089	UMHMNP211358666	−8.3	−9.4	−9.6
1046	UMHMNP848132354	−9.5	−9.4	−9.6	1090	UMHMNP866025630	−9.1	−9.3	−9.6
1047	UMHMNP876756502	−8.5	−9.7	−9.6	1091	UMHMNP191112193	−9.3	−9.3	−9.6
1048	UMHMNP473256223	−8.7	−9.9	−9.6	1092	UMHMNP155233311	−8.5	−9.8	−9.6
1049	UMHMNP6758710	−9.5	−10.7	−9.6					

[a]Data sorted according to stage III docking score.

Table S3. Estimated fast, moderate, and expensive docking scores and MM-GBSA binding energies (in kcal/mol) over 1 ns implicit water solvent MD simulations for XF7 and the top 111 potent MNPs compounds within the 3CL^{pro} binding pocket.[a]

No.	Compound Name/Code	Docking Score (kcal/mol)			MM-GBSA Binding Energy (kcal/mol)
		Fast	Moderate	Expensive	
	XF7	−8.1	−9.2	−9.5	−40.0
1	UMHMNP26195584	−11.1	−11.5	−11.5	−45.3
2	UMHMNP133056094	−11.2	−11.5	−11.5	−44.9
3	UMHMNP14984668	−11.3	−11.7	−12.2	−44.8
4	UMHMNP148839036	−11.3	−11.6	−11.8	−44.0
5	UMHMNP1403367	−11.7	−12.2	−13.4	−42.5
6	UMHMNP101691127	−11.4	−12.1	−12.3	−42.2
7	UMHMNP791849666	−11.4	−11.9	−12.3	−41.8
8	UMHMNP143621754	−11.3	−11.7	−12.2	−41.5
9	UMHMNP133056072	−11.3	−11.6	−11.7	−41.3
10	UMHMNP386274857	−11.3	−11.6	−11.7	−40.6
11	UMHMNP874383707	−11.1	−11.5	−11.4	−40.5
12	UMHMNP221163300	−11.1	−11.4	−11.4	−40.2
13	UMHMNP109152387	−11.1	−11.4	−11.4	−40.2
14	UMHMNP156953912	−11.1	−11.4	−11.4	−39.7
15	UMHMNP706784727	−10.9	−11.3	−11.4	−39.6
16	UMHMNP5035303	−11.0	−11.4	−11.4	−39.6
17	UMHMNP77982800	−10.9	−11.2	−10.5	−39.5
18	UMHMNP73622238	−9.9	−10.1	−10.4	−39.4
19	UMHMNP64421201	−8.8	−9.5	−10.7	−39.3
20	UMHMNP745075278	−9.9	−10.0	−10.6	−39.1
21	UMHMNP147362376	−8.3	−9.3	−11.3	−38.7
22	UMHMNP852872925	−8.4	−9.7	−10.7	−38.3
23	UMHMNP552839802	−8.3	−9.3	−10.5	−38.1
24	UMHMNP151078921	−8.8	−10.7	−10.8	−38.0
25	UMHMNP64548169	−9.3	−9.5	−10.7	−37.7
26	UMHMNP70329281	−9.1	−10.1	−10.5	−37.5
27	UMHMNP157207915	−9.2	−10.8	−11.3	−37.3
28	UMHMNP151606405	−9.4	−9.8	−10.8	−36.6
29	UMHMNP86708329	−10.6	−10.7	−10.8	−36.5
30	UMHMNP664992272	−9.0	−10.1	−11.0	−36.4
31	UMHMNP106231258	−9.7	−9.7	−11.0	−36.4
32	UMHMNP474395	−8.2	−9.9	−10.5	−36.3
33	UMHMNP244157957	−8.7	−11.2	−10.5	−36.3
34	UMHMNP741259858	−9.1	−9.3	−10.5	−35.9
35	UMHMNP244157946	−8.6	−9.7	−10.7	−35.5
36	UMHMNP70139569	−8.2	−9.3	−10.7	−35.4
37	UMHMNP139765370	−8.1	−10.7	−10.8	−35.4
38	UMHMNP54369118	−9.0	−9.4	−11.0	−35.2
39	UMHMNP116407160	−8.7	−9.5	−11.3	−35.0
40	UMHMNP351443759	−8.8	−9.9	−10.7	−34.9
41	UMHMNP153212863	−8.2	−11.2	−11.2	−34.9
42	UMHMNP221163311	−9.6	−10.5	−10.8	−34.7
43	UMHMNP153212852	−8.4	−9.7	−11.3	−34.4
44	UMHMNP176447964	−9.0	−9.4	−11.2	−34.4
45	UMHMNP868395884	−9.1	−9.6	−10.5	−34.2
46	UMHMNP58670636	−9.5	−11.0	−11.0	−34.2
47	UMHMNP116302386	−9.4	−9.5	−10.7	−34.1
48	UMHMNP6673661	−9.6	−10.9	−10.9	−34.1
49	UMHMNP41410586	−8.4	−9.8	−10.7	−33.9

Table S3. *Continued.*

No.	Compound Name/Code	Docking Score (kcal/mol)			MM-GBSA Binding Energy (kcal/mol)
		Fast	Moderate	Expensive	
50	UMHMNP862286702	−9.3	−9.7	−10.5	−33.9
51	UMHMNP148010406	−8.9	−10.6	−11.3	−33.8
52	UMHMNP107503093	−8.2	−9.7	−11.2	−33.6
53	UMHMNP124689652	−8.9	−9.4	−10.7	−33.3
54	UMHMNP150079951	−8.7	−9.3	−10.5	−33.2
55	UMHMNP131467000	−10.7	−10.8	−10.8	−33.0
56	UMHMNP393870332	−8.5	−9.3	−11.0	−32.9
57	UMHMNP253195403	−9.4	−9.5	−10.5	−32.9
58	UMHMNP184885890	−8.2	−9.3	−11.3	−32.8
59	UMHMNP142796314	−8.1	−10.7	−10.5	−32.7
60	UMHMNP174630070	−10.9	−11.0	−10.9	−32.5
61	UMHMNP272458341	−10.0	−10.3	−10.7	−32.5
62	UMHMNP125329091	−10.9	−9.9	−10.9	−32.2
63	UMHMNP86748314	−8.1	−9.3	−11.0	−32.2
64	UMHMNP171674923	−8.9	−10.8	−10.9	−32.1
65	UMHMNP260436291	−9.5	−9.7	−11.0	−32.0
66	UMHMNP757976673	−9.7	−9.8	−11.0	−31.9
67	UMHMNP827348072	−9.7	−9.7	−10.5	−31.9
68	UMHMNP100942743	−8.8	−11.2	−11.3	−31.9
69	UMHMNP142755133	−8.5	−9.3	−10.4	−31.8
70	UMHMNP54028465	−9.9	−9.8	−11.0	−31.7
71	UMHMNP162830264	−9.5	−10.4	−11.3	−31.6
72	UMHMNP745075245	−8.2	−9.3	−10.4	−31.3
73	UMHMNP116303	−9.5	−9.4	−11.2	−31.3
74	UMHMNP74185049	−10.0	−10.1	−10.6	−31.1
75	UMHMNP129350181	−8.1	−9.3	−10.5	−30.8
76	UMHMNP823808555	−8.4	−10.1	−11.3	−30.6
77	UMHMNP4030926	−8.8	−9.3	−10.7	−30.5
78	UMHMNP374819086	−8.9	−9.6	−10.5	−30.3
79	UMHMNP22033870	−8.6	−9.7	−11.3	−30.2
80	UMHMNP152110093	−10.2	−9.3	−10.6	−29.9
81	UMHMNP6673683	−9.5	−9.7	−10.6	−29.8
82	UMHMNP131466972	−8.2	−9.3	−11.3	−29.7
83	UMHMNP150050138	−10.7	−10.7	−11.3	−29.7
84	UMHMNP444987637	−8.3	−9.9	−10.6	−29.6
85	UMHMNP53296836	−9.9	−9.3	−11.2	−29.2
86	UMHMNP890041722	−10.8	−10.9	−10.9	−29.1
87	UMHMNP149764341	−8.9	−9.6	−11.3	−29.1
88	UMHMNP165815816	−8.6	−9.8	−10.6	−28.6
89	UMHMNP272458330	−9.2	−9.3	−10.7	−27.8
90	UMHMNP154205182	−9.9	−11.2	−11.3	−27.3
91	UMHMNP80981379	−9.2	−9.4	−10.4	−27.3
92	UMHMNP290824474	−8.9	−9.5	−10.5	−27.2
93	UMHMNP386274868	−8.8	−10.3	−10.4	−27.1
94	UMHMNP105404840	−10.0	−10.1	−11.3	−26.9
95	UMHMNP119108360	−8.9	−9.7	−11.1	−26.2
96	UMHMNP61369275	−8.7	−10.0	−11.0	−26.0
97	UMHMNP76915247	−9.6	−9.7	−11.3	−25.6
98	UMHMNP221169740	−8.4	−9.6	−11.3	−25.4
99	UMHMNP100942754	−9.1	−10.7	−11.3	−25.2
100	UMHMNP161470231	−8.9	−10.6	−10.9	−24.3
101	UMHMNP99528877	−10.4	−10.4	−10.4	−24.0
102	UMHMNP162830231	−8.8	−9.3	−10.7	−23.0
103	UMHMNP94203539	−9.9	−10.0	−11.0	−21.8
104	UMHMNP149444928	−9.1	−9.4	−11.3	−21.6
105	UMHMNP167172987	−8.4	−9.5	−11.3	−21.5

Table S3. *Continued.*

No.	Compound Name/Code	Docking Score (kcal/mol)			MM-GBSA Binding Energy (kcal/mol)
		Fast	Moderate	Expensive	
106	UMHMNP143049143	−8.4	−9.3	−11.0	−21.4
107	UMHMNP157622601	−8.5	−9.8	−11.2	−16.5
108	UMHMNP143049165	−8.9	−10.6	−11.0	−12.2
109	UMHMNP2122987	−9.4	−9.4	−10.4	−11.9
110	UMHMNP2497747	−9.3	−9.3	−10.7	−11.8

[a]Data sorted according to MM-GBSA binding energy over 1 ns implicit water solvent MD simulation.

Table S4. Estimated fast, moderate, and expensive docking scores and MM-GBSA binding energies (in kcal/mol) over 1 ns implicit water solvent and 5 ns explicit water solvent MD simulations for XF7 and the top 13 potent MNPs compounds within the $3CL^{pro}$ binding pocket.[a]

No.	Compound Name/Code	Docking Score (kcal/mol)			MM−GBSA Binding Energy (kcal/mol)	
		Fast	Moderate	Expensive	1 ns	5 ns
	XF7	−8.1	−9.2	−9.5	−40.0	−43.0
1	UMHMNP1403367	−11.7	−12.2	−13.4	−42.5	−55.2
2	UMHMNP101691127	−11.4	−12.1	−12.3	−42.2	−45.7
3	UMHMNP791849666	−11.4	−11.9	−12.3	−41.8	−45.1
4	UMHMNP14984668	−11.3	−11.7	−12.2	−44.8	−44.4
5	UMHMNP143621754	−11.3	−11.7	−12.2	−41.5	−42.4
6	UMHMNP148839036	−11.3	−11.6	−11.8	−44.0	−39.6
7	UMHMNP133056072	−11.3	−11.6	−11.7	−41.3	−38.4
8	UMHMNP386274857	−11.3	−11.6	−11.7	−40.6	−36.3
9	UMHMNP133056094	−11.2	−11.5	−11.5	−44.9	−35.4
10	UMHMNP26195584	−11.1	−11.5	−11.5	−45.3	−32.8
11	UMHMNP874383707	−11.1	−11.5	−11.4	−40.5	−32.5
12	UMHMNP221163300	−11.1	−11.4	−11.4	−40.2	−28.0
13	UMHMNP109152387	−11.1	−11.4	−11.4	−40.2	−27.9

Table S5. The estimated MM-GBSA binding energies (in kcal/mol) and relative binding energies ($\Delta\Delta G_{binding}$) over 25 ns explicit water solvent MD simulations for XF7 and the top 5 potent MNPs compounds within the $3CL^{pro}$ binding pocket.

Compound Name/Code	$\Delta G_{binding}$ (kcal/mol)	$\Delta\Delta G_{binding}$ (kcal/mol)[a]
XF7	−45.0	---
UMHMNP1403367	−57.5	−12.5
UMHMNP143621754	−46.8	−1.8
UMHMNP14984668	−45.9	−0.9
UMHMNP791849666	−45.3	−0.3
UMHMNP101691127	−45.2	−0.2

[a] $\Delta\Delta G_{binding} = \Delta G_{MNP} - \Delta G_{XF7}$
